# Multiple local PfDHFR I164L haplotype expansions drive *Plasmodium falciparum* antifolate resistance in Uganda

**DOI:** 10.1038/s41467-026-76826-4

**Published:** 2026-09-04

**Authors:** Victor Asua, Karamoko Niaré, Shreeya Garg, Jenny Legac, Stephen Tukwasibwe, Thomas Katairo, Francis E. Bohissou, Rebecca M. Crudale, Alfred Simkin, Jerry Mulondo, Sam L. Nsobya, Isaac Ssewanyana, Grant Dorsey, Jeffrey A. Bailey, Moses R. Kamya, Adoke Yeka, Philip J. Rosenthal, Steffen Borrmann, Melissa D. Conrad

**Affiliations:** 1 Institute for Tropical Medicine, University of Tübingen, Tübingen, Germany; 2 Infectious Diseases Research Collaboration, Kampala, Uganda; 3 German Centre for Infection Research, Partner site Tübingen, Tübingen, Germany; 4 Department of Pathology and Laboratory Medicine, Brown University, Providence, RI USA; 5 Pathogens Genomics Diversity Network Africa, Bamako, Mali; 6 University of California San Francisco, San Francisco, CA USA; 7 Makerere University, Kampala, Uganda; 8 Institut de Recherche en Sciences de la Santé /Clinical Research Unit of Nanoro, Nanoro, Burkina Faso; 9 Centre de Recherche Entomologique de Cotonou, Cotonou, Benin; 10 Centre for Computational Molecular Biology, Brown University, Providence, RI USA; 11 Johns Hopkins Malaria Research Institute, Department of Molecular Microbiology and Immunology, Bloomberg School of Public Health, Johns Hopkins University, Baltimore, MD USA

**Keywords:** Parasite genomics, Parasite evolution, Malaria, Epidemiology, Antimicrobial resistance

## Abstract

Mutations in the *Plasmodium falciparum* genes, *pfdhfr* and *pfdhps*, drive antifolate resistance and threaten malaria control in regions where sulfadoxine-pyrimethamine (SP) is the primary chemoprevention strategy. The spatial patterns and evolutionary dynamics of these mutations in high-transmission settings remain incompletely understood. Here we genotyped 11 resistance-associated mutations in *pfdhfr* and *pfdhps* in 4,725 *P. falciparum* isolates collected from 16 Ugandan health facilities as part of annual surveillance between 2016 and 2022. Notably, we show that the frequency of PfDHFR I164L, which confers higher pyrimethamine resistance, increased over time from 19.4% to 32.4%. Using identity-by-descent, haplotype structure, and extended haplotype homozygosity analyses, we show that PfDHFR I164L is present on multiple haplotype backgrounds and undergoes localised expansions, without detectable signatures of recent positive selection at all but one site. Our results suggest that the evolution of antifolate resistance, driven by PfDHFR I164L, is spatially heterogeneous and complex in regions that primarily use SP chemoprevention programmes.

## Abstract (web summary)

PfDHFR haplotypes involving I164L are known to strongly influence antifolate resistance in Plasmodium falciparum. In this study, Asua and colleagues examine the frequency of antifolate-resistance conferring mutations in over 4,700 parasite samples collected in Uganda over a 6-year period. They report that markers conferring antifolate resistance during this time, which may impact chemotherapeutic strategies that are reliant on sulfadoxine-pyrimethamine (SP).

## Introduction

Malaria remains a critical public health challenge in sub-Saharan Africa, where the vast majority of the estimated 282 million cases and 610,000 deaths occurred in 2024^1^. Despite substantial gains in its control over the past two decades^2,3^, the emergence and spread of antimalarial drug resistance threatens to reverse progress, particularly in high-burden regions of Africa.

The antifolate sulfadoxine-pyrimethamine (SP) has been central for malaria prevention, including intermittent preventive treatment in pregnancy (IPTp-SP), perennial malaria chemoprevention, and seasonal malaria chemoprevention (SMC, in combination with amodiaquine) in areas with seasonal malaria transmission^1,4–6^. In Uganda, SP is widely used, particularly as IPTp-SP, and more recently for SMC in the Karamoja region^1,5,7,8^. The continued SP use may promote the selection and transmission of antifolate-resistant parasites, complicating malaria control efforts.

Sulfadoxine and pyrimethamine target *P. falciparum* dihydropteroate synthase (PfDHPS) and dihydrofolate reductase (PfDHFR), respectively, thereby blocking DNA synthesis and parasite multiplication^9,10^. Resistance arises through the stepwise accumulation of point mutations in these genes^11–13^, including the well-characterised PfDHFR triple (N51I, C59R, S108N) and PfDHPS double (A437G, K540E) mutation haplotypes, which together form a mutant genotype strongly associated with reduced SP efficacy ^14–18^.

In East Africa, the quintuple mutant genotype described above is now widespread^19–22^ and provides a background on which additional resistance-conferring mutations can emerge. Notably, the PfDHFR I164L and PfDHPS A581G mutations, which were previously uncommon across most of Africa, have been reported in Uganda and neighbouring regions, including the Democratic Republic of the Congo, Rwanda, and Tanzania^22–25^. These mutations are of particular concern due to their association with high-level resistance to pyrimethamine and sulfadoxine, thereby diminishing SP effectiveness^16–19,26,27^. Despite their importance, the current distribution, evolutionary trajectories and local adaptation of these alleles remain poorly characterised.

Previous surveillance efforts have focused primarily on the prevalence of resistance-associated mutations^21,22,28,29^, providing limited insight into the broader evolutionary dynamics underlying their continued transmission. In contrast, population genomic approaches such as the study of identity-by-descent (IBD) and haplotype structure enable detailed characterisation of the evolutionary trajectory of these mutations^30–33^. However, these methods have been applied sparingly, particularly for examining the evolution of antifolate resistance in large-scale, longitudinal studies across malaria-endemic regions ^34–36^.

In this study, we report the spatiotemporal prevalence of antifolate resistance-associated alleles and the genetic signatures underlying their evolution, with a particular emphasis on the PfDHFR I164L mutation whose frequency remains heterogeneous in Uganda.

## Results

### Sampling and genotyping of field isolates

**Fig. 1:**
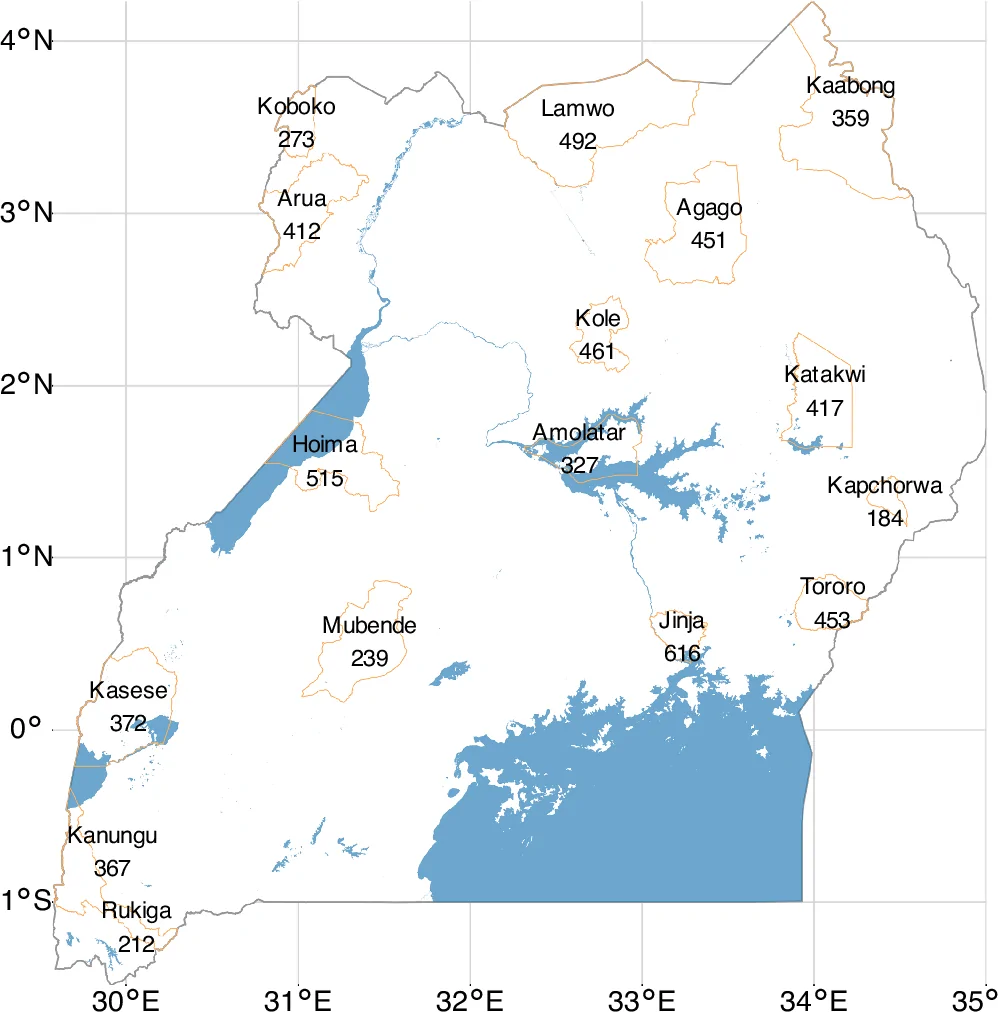
Map of Uganda with sampled districts. Districts are labelled with the district name and the total number of samples collected (in brackets).

We collected dried blood spots annually from 10 (2016–2017) to 16 (2018–2022) sites across Uganda (Fig. 1). Samples were subjected to targeted amplicon deep sequencing using molecular inversion probes (MIPs) designed to capture (i) polymorphic genomic regions associated with antimalarial drug resistance, (ii) 100-kb regions flanking these genes, and (iii) genome-wide putatively neutral single nucleotide polymorphisms (SNPs). From this sequencing, we previously reported the spatiotemporal prevalence of mutations associated with artemisinin partial resistance^37,38^. Here, we focus on the spatial and temporal evolutionary dynamics of antifolate resistance-associated mutations, including five in PfDHFR and six in PfDHPS. Prevalence estimates using a different approach from 2016–2019 were previously reported^25,39^, but are included in this analysis for assessment of temporal pattern. Of the 11 targeted drug resistance loci, at least one was successfully genotyped in 4725 samples. Among these, 3322 (70.3%) were successfully genotyped at all 11 loci, 904 (19.1%) at 5–10 loci, and 499 (10.6%) at fewer than five loci.

### Spatial and temporal prevalence of antifolate resistance-associated mutations

**Fig. 2:**
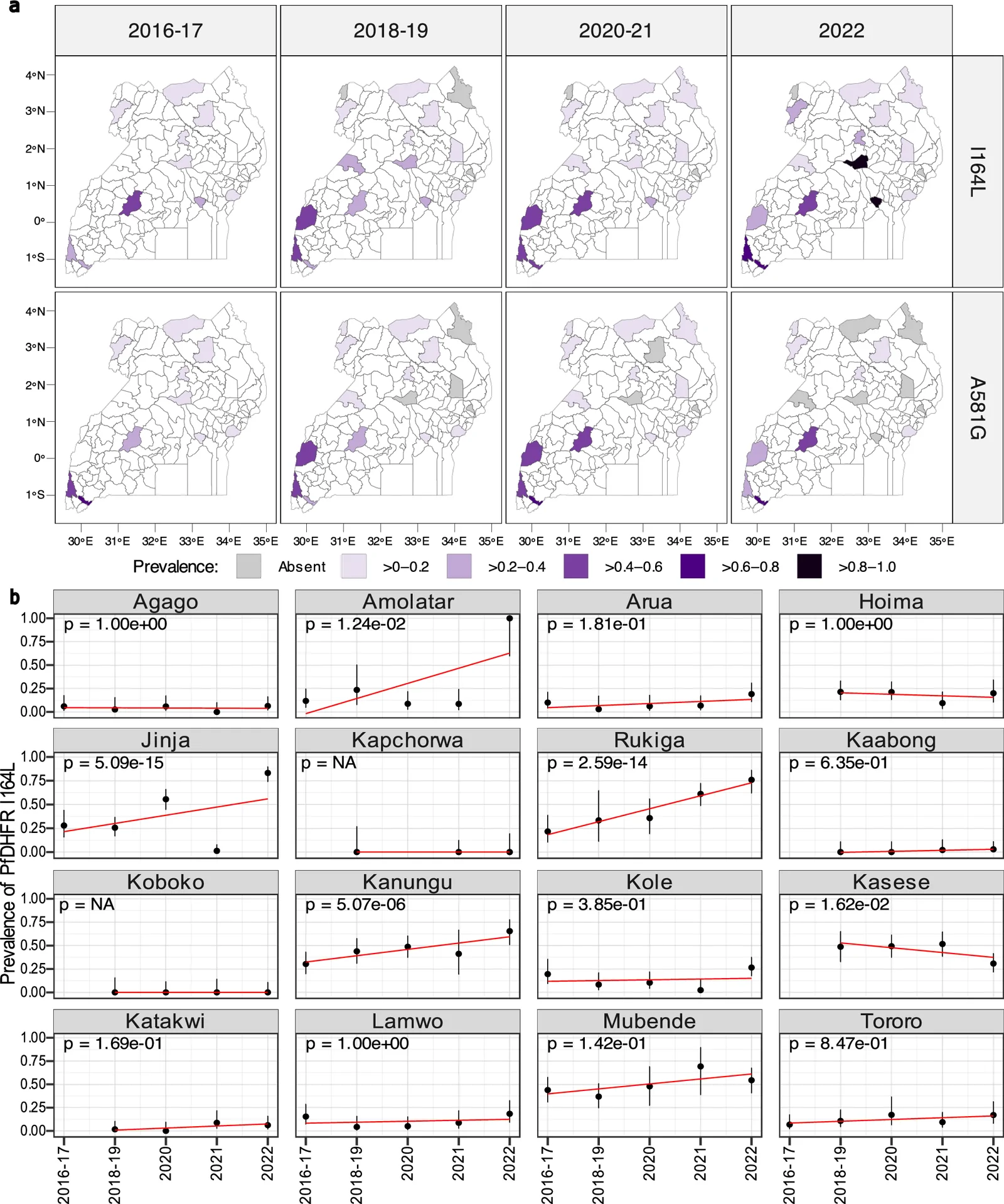
Spatial and temporal prevalence of PfDHFR I164L and PfDHPS A581G mutations. **a** Geographic distribution of binned mutation prevalences across sampling years at each site. Sampling districts are coloured according to the proportion of mutant alleles. **b** Temporal prevalence of the PfDHFR I164L mutation over time. The y-axis shows the proportion of samples carrying mutant or mixed genotypes, based on 4725 samples. Points represent annual prevalence estimates with 95% confidence intervals. Red lines indicate trends from linear models, with Bonferroni-adjusted *p*-values. The number of isolates sequenced per site can be found in Supplementary Table 1. Source data are provided as a Source Data file.

Among the antifolate resistance-associated mutations analysed, five (PfDHFR N51I, C59R, S108N, and PfDHPS A437G, K540E) were fixed or nearly fixed (prevalence > 90%) across all sites and time points. Mutations mainly observed outside East Africa (PfDHFR A16V and PfDHPS A431V and A613S) were absent. The PfDHFR I164L and PfDHPS A581G mutations that are associated with even greater antifolate resistance exhibited marked spatial and temporal variation (Supplementary Table 1 and Fig. 2).

The overall prevalence of the PfDHFR I164L mutation varied over the study period, from 19.4% (95% CI: 16.1–32.1) in 2016-17 to 32.4% (95% CI: 29.4–35.6) in 2022, with short term fluctuations, but an upward trend (odds ratio = 1.11, 95% CI: 1.07–1.16, *p* value < 0.001). In contrast, PfDHPS A581G prevalence ranged from 21.3% (95% CI: 18.2–24.8) in 2016-17 to 16.1% (95% CI: 14.0–18.5) in 2022, showing no temporal trend (odds ratio = 0.97, 95% CI: 0.94–1.0, *p* value = 0.161). Both PfDHFR I164L and PfDHPS A581G were detected at heterogeneous frequencies across sites and time points (Fig. 2a).

To assess site-specific trends of PfDHFR I164L mutation prevalence, we fitted linear regression models for each site to obtain a summary of the overall temporal trends, although it should be noted that not all trajectories, particularly that of Jinja, were monotonic. Significant increases were observed at Jinja (*p* value < 0.001), Rukiga (*p* value < 0.001), and Kanungu (*p* value = 0.005), while moderate but non-significant increases were seen at Amolatar (*p* value = 0.2) and Mubende (*p* value = 0.9). At other sites, the mutation was absent (Kapchorwa, Koboko) or was seen only at very low prevalences (Agago, Kaabong, Katakwi) (Fig. 2b).

These results indicate substantial spatial and temporal heterogeneity in PfDHFR I164L prevalence. To characterise the genetic background of its evolution, we conducted additional complementary analyses: (1) assessing genome-wide relatedness by IBD sharing, stratified by PfDHFR I164L mutation status, (2) IBD network analysis to infer clustering of related infections, (3) analysis of haplotype structure to assess local haplotypes carrying the allele, and (4) extended haplotype homozygosity (EHH) decay to detect recent positive selection.

### IBD sharing patterns associated with resistance alleles

**Fig. 3:**
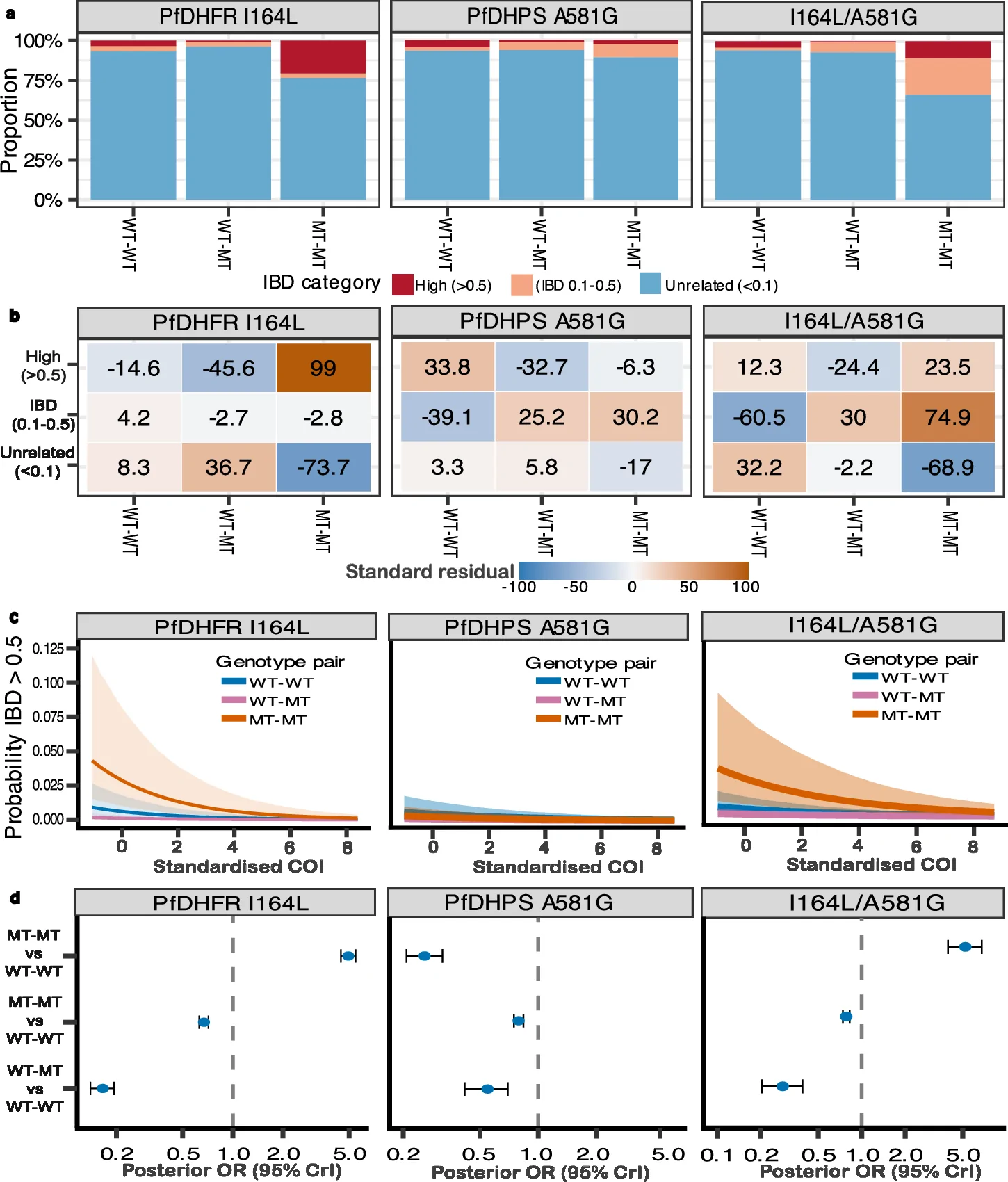
Patterns of IBD sharing. Analysis was performed on sample pairs with PfDHFR I164L and PfDHPS A581G genotype data, categorised as WT-WT (both parasites carrying the wild-type allele), MT-MT (both parasites carrying mutant allele), and WT-MT (discordant genotype pairs). **a** Distributions of highly related (IBD > 0.5) and unrelated (IBD < 0.1) parasite pairs are shown. Chi-square tests of independence were used to compare the distributions as descriptive differences rather than formal hypothesis tests of independent observations. **b** Heatmaps show standardised residuals from the Chi-square test, with dark orange tiles indicating observed counts higher than expected and blue tiles indicating lower counts. **c**, **d** Results from Bayesian hierarchical logistic regression models of high relatedness (IBD > 0.5) incorporating genotype pair and standardised average complexity of infection as fixed effects, with sampling site included as a random intercept. **c** Shows predicted probability of high identity-by-descent (IBD > 0.5) as a function of standardised average complexity of infection for PfDHFR I164L, PfDHPS A581G and I164L/A581G; and **d** shows posterior odds ratios (points) with corresponding 95% credible intervals (horizontal lines) for PfDHFR I164L, PfDHPS A581G and I164L/A581G. Source data are provided as a Source Data file.

To determine whether resistance-associated alleles were enriched among genetically related parasite pairs, we analysed pairwise IBD between samples. Pairs were classified as MT-MT (both parasites carrying the mutant allele), WT-MT (discordant genotype pairs), or WT-WT (both parasites carrying the wild-type allele). For ~3500 samples and ~1500 SNPs, we obtained ~6.24 million pairwise IBD comparisons, of which over 3 million pairs had complete PfDHFR I164L and PfDHPS A581G genotype data. We classified pairs into highly related (IBD > 0.5), intermediate (IBD 0.1–0.5), and unrelated (IBD < 0.1) categories and tested associations between genotype-pair class and IBD category using chi-square tests of independence. Intermediate IBD values (0.1–0.5) were not explicitly interpreted because the sparse SNP density reduces confidence in resolving the IBD estimates, whereas extreme IBD categories provide more robust inference^33^. Considering PfDHFR I164L, PfDHPS A581G, and their combination, we observed a significant association between genotype and parasite relatedness. The strongest deviation from independence was observed for PfDHFR I164L (*χ*² = 10,242, *p* value < 0.001), followed by the combined I164L/A581G genotype (*χ*² = 1349, *p* value < 0.001) and PfDHPS A581G alone (*χ*² = 1125, *p* value < 0.001) (Fig. 3a, b).

For PfDHFR I164L, mutant parasites were enriched for high IBD (IBD > 0.5; standard residuals [Std. resid] = 99), with reciprocal depletion of wild-type–mutant ( std. resid = −45.6). A converse pattern was observed for unrelated parasites (IBD < 0.1). PfDHPS A581G exhibited a different pattern. Although the association between genotype and relatedness was significant (*χ*² = 1125, *p* value < 0.001), mutant pairs were not enriched for relatedness (Std. resid = −6.3), but instead wild-type pairs were moderately enriched for relatedness (Std. resid = 33.8) (Fig. 3a, b). These observations indicate that PfDHFR I164L mutant parasites are more likely to be closely related than parasites carrying PfDHPS A581G without I164L mutation. Parasites carrying both the I164L and A581G mutations showed an intermediate pattern of genetic relatedness, with enrichment of mutant pairs for relatedness (Std. resid = 23.5), depletion of mutant–wild-type pairs (Std. resid = −24.4), and modest enrichment among wild-type pairs (Std. resid = 12.3) (Fig. 3a, b).

Bayesian hierarchical logistic regression models incorporating site-level variation and average complexity of infection (AvgCOI) identified PfDHFR I164L as the primary genetic correlate of high relatedness (Fig. 3c, d). Across models, substantial among-site heterogeneity was observed (site-level SD of intercepts: 2.14–2.43), indicating strong spatial variation in baseline high IBD probability. Variation among PfDHFR I164L pairs showed strong genotype-specific effects: the WT-MT genotype was associated with markedly reduced high IBD odds (OR = 0.17, 95% CrI: 0.14–0.19), whereas the MT-MT genotype was associated with increased odds (OR = 4.95, 95% CrI: 4.48–5.47). In contrast, PfDHPS A581G pairs showed weaker and consistently negative associations, with reduced odds of high IBD for both WT-MT (OR = 0.26, 95% CrI: 0.21–0.32) and MT-MT (OR = 0.55, 95% CrI: 0.42–0.71). The combined I164L/A581G genotype pair recapitulated the PfDHFR I164L pattern, with MT-MT combination showing elevated IBD odds (OR = 5.21, 95% CrI: 3.94–6.75) and WT-MT combinations showing reduced odds (OR = 0.28, 95% CrI: 0.20–0.39) (Fig. 3c, d). The similarity between PfDHFR I164L and I164L/A581G models suggests that the observed association is largely driven by PfDHFR I164L, with limited contribution from PfDHPS A581G. Across all models, higher AvgCOI was negatively associated with high IBD probability (OR = 0.67–0.79), indicating a consistent inverse relationship after accounting for genotype and site effects (Fig. 3c).

### IBD sharing patterns at individual geographic sites

**Fig. 4:**
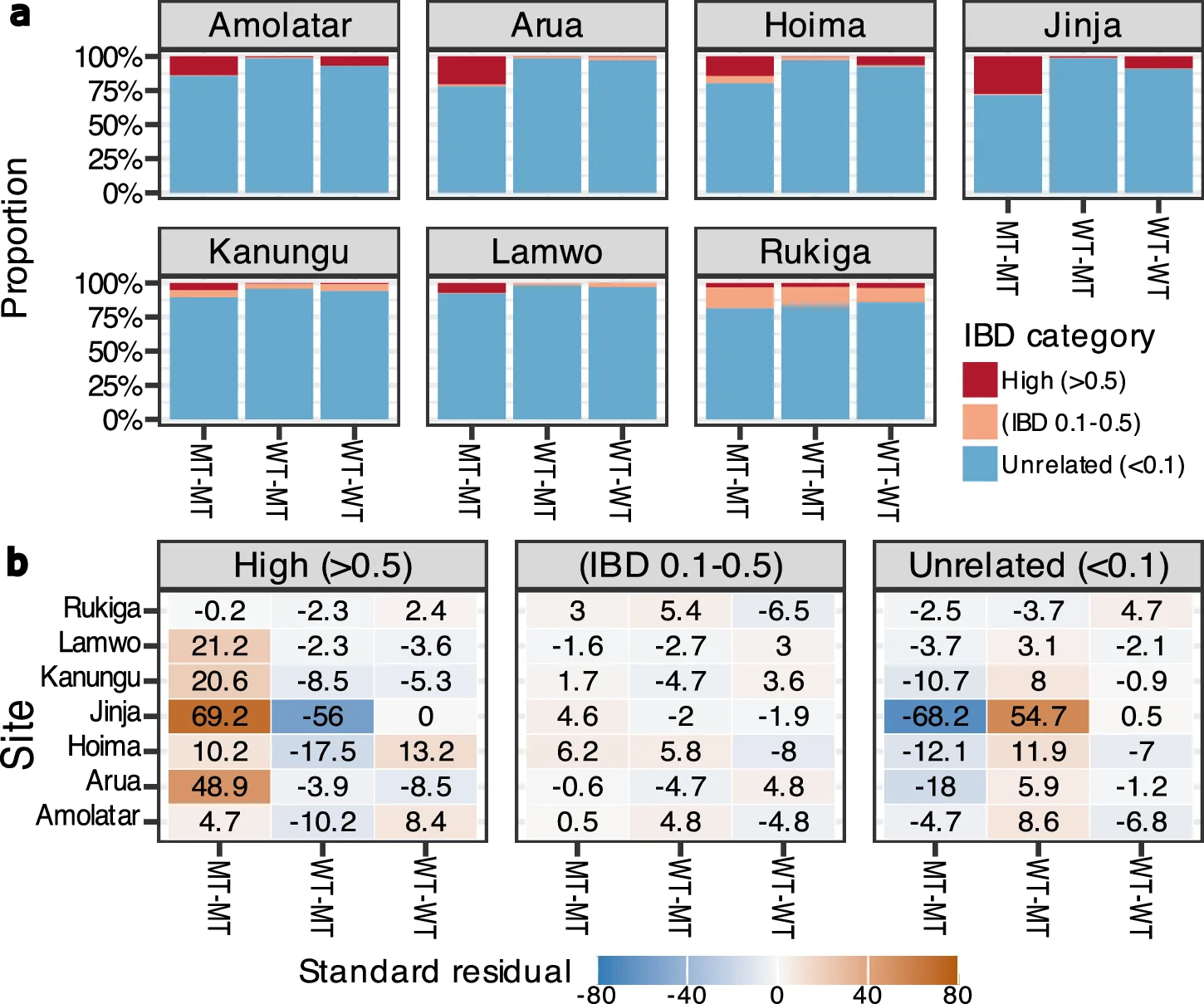
Site-specific patterns of IBD sharing among PfDHFR I164L genotype pairs. **a** Distributions of highly related (IBD > 0.5) and unrelated (IBD < 0.1) parasite pairs among WT-WT, MT-MT, and WT-MT PfDHFR I164L genotype combinations across sampling sites. **b** Heatmaps showing standardised residuals from Chi-square tests, with dark orange indicating observed counts greater than expected and blue indicating counts lower than expected. Analyses include only sites in which mutant PfDHFR I164L pairs were detected. Source data are provided as a Source Data file.

To determine whether these patterns were consistent across sites, we repeated chi-square tests of independence between genotype-pair class and IBD category within each sampling site. The proportions of PfDHFR I164L genotypes (WT-WT, WT-MT and MT-MT) between highly related (IBD > 0.5) and unrelated (IBD < 0.1) categories revealed patterns consistent with overall analysis (Fig. 4a). Enrichment of PfDHFR I164L mutant pairs for high relatedness (IBD > 0.5) was greatest in Jinja (std. resid = 69.2), followed by Arua (std. resid = 48.9), Lamwo (std. resid = 21.2) and Kanungu (std. resid = 20.6) (Fig. 4b).

The enrichment of PfDHFR I164L mutant pairs for high relatedness at selected sites is consistent with recent common ancestry and local expansion. In contrast, the absence of comparable enrichment, or the presence of intermediate patterns, among PfDHPS A581G mutants and wild-type parasites may indicate weaker or more heterogeneous selection. However, demographic structure, transmission, recombination, and sampling effects may also contribute.

### IBD cluster and network analysis

**Fig. 5:**
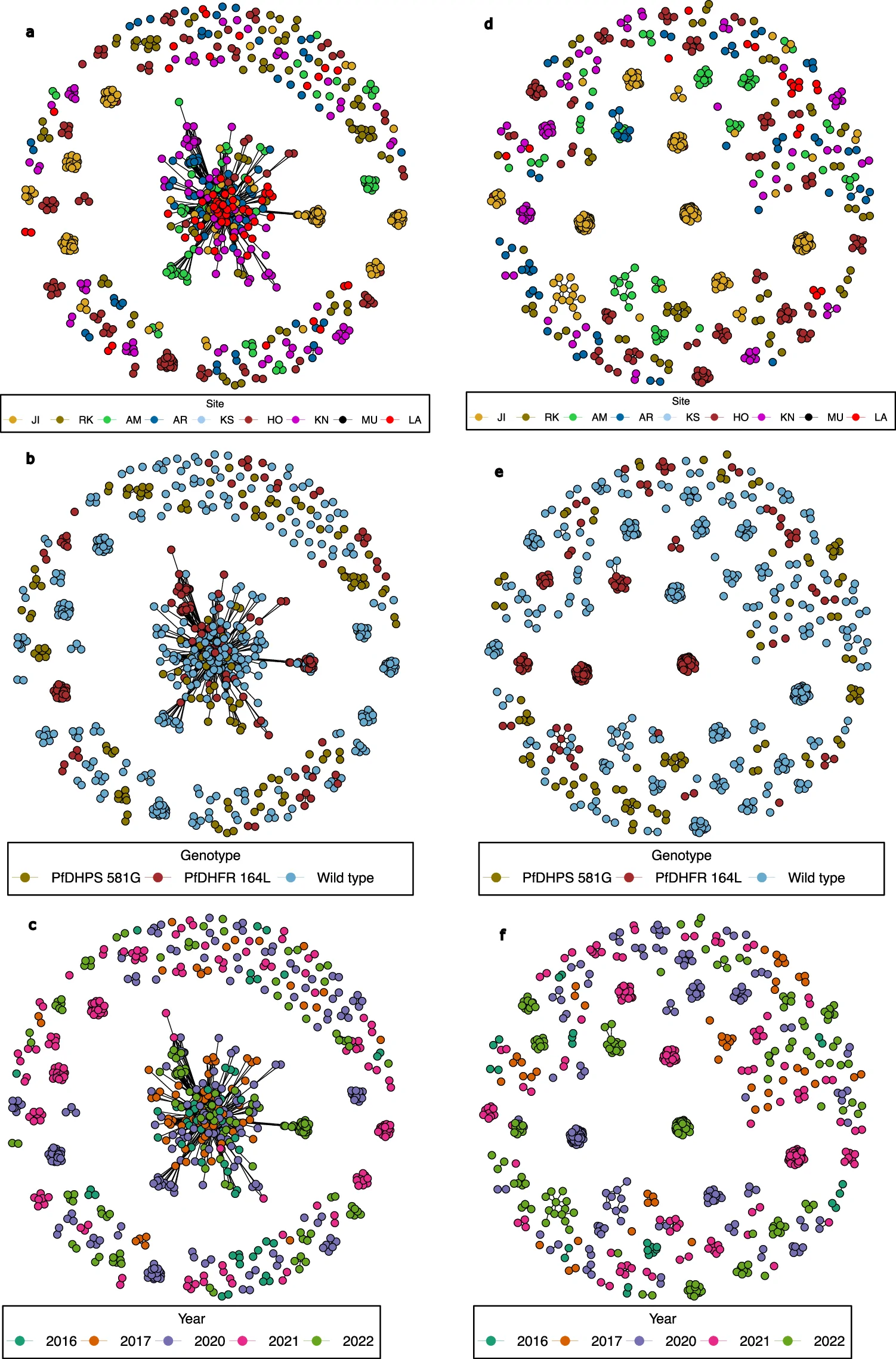
IBD networks. IBD networks constructed using thresholds of ≥ 0.5 (**a**–**c**) and ≥ 0.9 (**d**–**f**). Each node represents a unique parasite isolate, and edges indicate IBD sharing between isolates. Unconnected nodes represent isolates with no IBD above the specified thresholds. Node colours indicate sample collection site (**a**, **d**), genotype (**b**, **e**), and year (**c**, **f**). Site names: JI = Jinja, RK = Rukiga, AM = Amolatar, AR = Arua, KS = Kasese, HO = Hoima, KN = Kanungu, MU=Mubende, LA=Lamwo. Source data are provided as a Source Data file.

Building on the pairwise IBD, we applied IBD network analysis to assess for clusters of relatedness across the parasite population. Considering a relatively low threshold of relatedness (IBD ≥ 0.5), IBD network plots showed extensive connectivity among parasites from different sites (Fig. 5a), genotypes (Fig. 5b) and years (Fig. 5c). In contrast, a more stringent threshold (IBD ≥ 0.9) revealed several independent clusters with high within-cluster connectivity (Fig. 5d–f). Using the IBD threshold of 0.90, we identified 24 large clusters comprising 10–51 isolates with high within-cluster connectivity. Isolates in these clusters were mostly from a single site, with three large clusters composed almost entirely of Jinja isolates, reflecting strong geographic structure. A few clusters spanned multiple sites, indicating inter-site connectivity. Clusters homogeneous for PfDHFR I164L genotypes were rare, and most contained both resistant and wild-type parasites (Supplementary Data 1). Most clusters were also largely confined to a single collection year, consistent with temporal structure in the parasite population (Fig. 5c, f).

These results demonstrate both localised expansions and spatial connectivity between sites, with no clear dominance of a single clonal transmission. Consistent with this interpretation, parasites within clusters were largely confined to single sites and collection years, although some clusters spanned multiple sites, indicating inter-site connectivity. Spatial connectivity is further reflected in the weak correlation between IBD sharing and geographic distance (Spearman’s ρ = −0.06) and the presence of highly related parasite pairs (IBD > 0.5) separated by >500 km (Supplementary Fig. 1).

### Haplotypes flanking the pfdhfr gene and the corresponding EHH decay

**Fig. 6:**
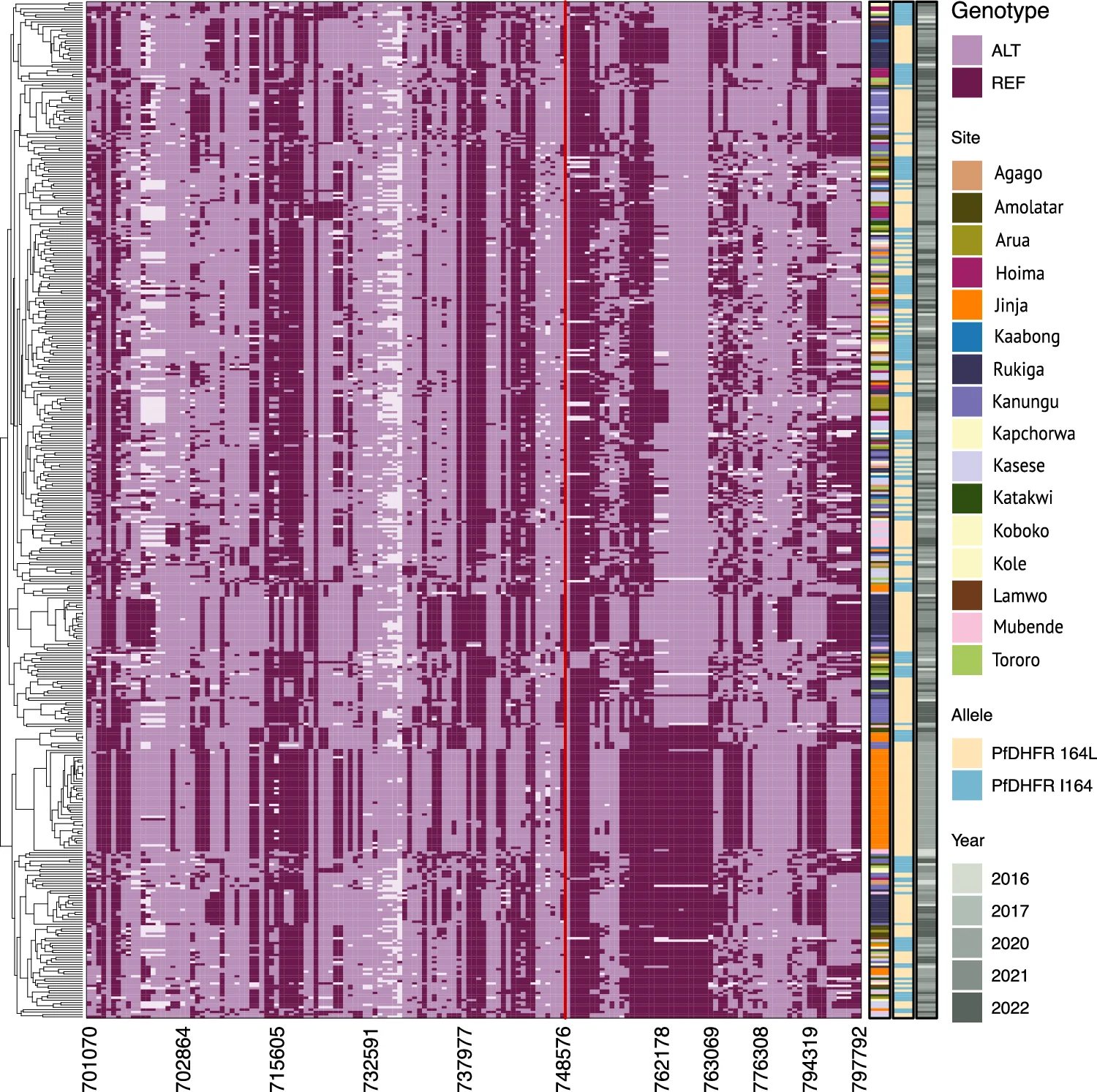
Haplotypes flanking the *pfdhfr* gene. Haplotype structure flanking a 50-kb region around the PfDHFR I164L locus. The heatmap shows haplotypes from 277 samples, hierarchically clustered based on Euclidean distance (y-axis), with chromosomal positions shown on the x-axis. The PfDHFR I164L locus is highlighted in red. Source data are provided as a Source Data file.

In recombining organisms, non-random locus-restricted extended haplotype sharing may reflect recent expansion and selection. Accordingly, to determine whether PfDHFR I164L increases were driven by positive selection, we analysed haplotype structure in the 50-kb region flanking the *pfdhfr* gene. We found that haplotypes were highly diverse, with no single haplotype predominating (Fig. 6). Contiguous clusters were observed for both mutant and wild-type parasites; however, haplotype patterns were largely mosaic, consistent with extensive recombination. In southwestern Uganda (Rukiga and Kanungu), multiple haplotypes were shared across sites, whereas in Jinja, PfDHFR I164L mutants exhibited several distinct haplotype blocks. Haplotype blocks were less apparent in Amolatar and Arua, likely reflecting smaller mutant sample sizes.

**Fig. 7:**
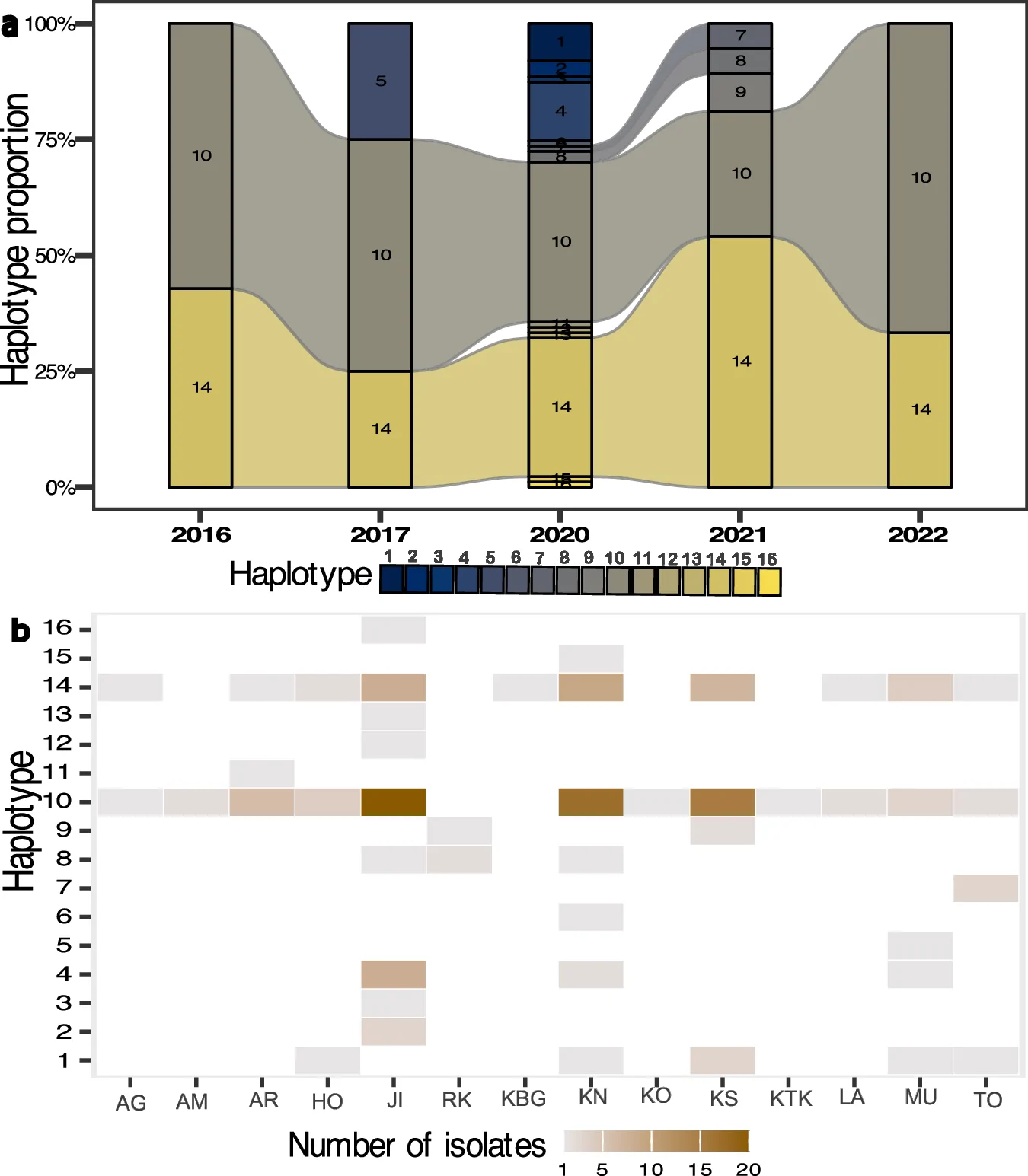
Temporal and geographic distribution of 10 kb flanking haplotypes around PfDHFR I164L. **a** Sankey plot illustrating temporal changes in haplotype frequencies from 2016 to 2022. Stream widths represent the relative proportion of each haplotype within each sampling year, and numbers indicate haplotype identities. **b** Heatmap showing the geographic distribution across sampling sites. Analyses included 177 samples harbouring the PfDHFR I164L mutation. Site names: AG = Agago, AM = Amolatar; AR = Arua, HO = Hoima, JI = Jinja, RK = Rukiga,  KBG = Kaabong, KN = Kanungu, KO = Kole, KS = Kasese, KTK = Katakwi, LA = Lamwo, MU = Mubende, TO = Tororo. Source data are provided as a Source Data file.

Narrowing the analysis window to 10-kb, we identified 16 haplotypes among 177 PfDHFR I164L mutant isolates (Fig. 7a, b). Two haplotypes dominated across years. In contrast, minor haplotypes fluctuated over time, consistent with ongoing diversification (Fig. 7a). Haplotype frequencies also varied geographically, with some widely distributed across Uganda and others restricted to specific sites (Fig. 7b). Together, these findings indicate that the *PfDHFR* I164L mutation is maintained on limited established haplotype backgrounds while exhibiting localised diversification.

EHH decay analysis across eight study sites (Supplementary Fig. 2) showed that, in three sites, mutant and wild-type parasites exhibited fast EHH decay, suggesting no recent selection of the resistant allele. In three sites (Rukiga, Kanungu and Lamwo), PfDHFR I164L mutants showed slightly slower EHH decay than wild-type, indicative of weak or historical local selection. In Jinja, PfDHFR I164L mutants exhibited pronounced slower EHH decay than wild-type parasites. Together, these findings are consistent with spatially heterogeneous selection acting on PfDHFR I164L, with no evidence of uniform recent selective sweep across Uganda.

## Discussion

While our findings mostly align with previous studies and underscore widespread antifolate resistance through near fixation of key PfDHFR (N51I, C59R, S108N) and PfDHPS (A437G, K540E) mutations^21,22,24,40^, the PfDHFR I164L mutation remains a remarkable exception, exhibiting variable spatio-temporal prevalences. Notably, this mutation, which is associated with higher levels of pyrimethamine resistance^12,13,41^, shows substantial heterogeneity, with genomic signatures consistent with spatially variable evolutionary dynamics.

Across sampling sites and time points, PfDHFR I164L allele frequencies varied, with multiple genetic backgrounds and spatial clustering of strains within potential localized transmission networks. The increasing prevalence of PfDHFR I164L at some, but not all, sites is consistent with spatially heterogeneous selection acting across different geographical settings, although other evolutionary factors also likely contribute. Although EHH decay analysis did not provide definitive evidence of strong recent selection, differences between mutant and wildtype haplotype selection were detected in Jinja, with more subtle signals in southwestern sites (Kanungu, Rukiga) and Lamwo, consistent with spatially heterogeneous evolutionary processes, including ongoing low-level selection.

The transmission of PfDHFR I164L mutants involved multiple parasite lineages, mostly within spatially structured populations, rather than expansion of a single or a few dominant backgrounds, as indicated by multiple IBD clusters and mosaic haplotype patterns from a 50-kb haplotype window. These evolutionary patterns are consistent with the high and heterogeneous malaria transmission in Uganda^42,43^, and the high recombination rate in *P. falciparum*^44^. However, analysis of the 10-kb flanking region identified two dominant PfDHFR I164L-associated haplotype backgrounds, consistent with multiple origins of the mutation. This finding corroborates previous evidence for multiple independent emergences of PfDHFR I164L in Kenya ^45^.

Drug selection pressure is likely an important contributor to the observed evolutionary dynamics, although its intensity may vary across space and time. Despite discontinuation of SP for treatment of uncomplicated malaria due to widespread resistance to sulfadoxine and pyrimethamine^14,46,47^, continued exposure to antifolate agents through chemoprevention during pregnancy, seasonal malaria chemoprevention^5,46^ and trimethoprim-sulfamethoxazole prophylaxis in HIV care^48,49^ may sustain low-level drug pressure on folate pathway genes. Importantly, the implementation and coverage for these interventions are often not uniform across space and time due to operational and supply chain challenges^50^. Such operational dynamics may contribute to spatial heterogeneity in drug pressure, leading to variable patterns of drug resistance evolution.

In high-transmission settings such as Uganda^42^, recombination plays a fundamental role in shaping patterns of *P. falciparum* genetic variation^44^. Frequent outcrossing erodes linkage disequilibrium arising from positive selection and redistributes alleles across multiple genetic backgrounds. The Bayesian models showed substantial site-level variation in baseline relatedness, highlighting strong effects of geographical structure. Higher AvgCOI reduced the probability of high IBD (OR = 0.67–0.79), consistent with recombination-driven breakdown in shared haplotype. These findings indicate that local transmission intensity and parasite diversity shape the selection of resistant lineages and contribute to heterogeneous genomic patterns across Uganda. By reshuffling resistant alleles, recombination can partly mask classical signatures of recent positive selection, such as observed in our EHH decay analysis^44,51^. In line with this expectation, the PfDHFR I164L allele was found in multiple haplotype backgrounds, and EHH analysis did not show strong evidence of recent positive selection^18^, even at sites where prevalence increased significantly. This pattern is compatible with the long period of historical circulation of the PfDHFR I164L allele in a recombining population; though it does not exclude alternative scenarios such as multiple emergences, repeated migration or low-level selection, which could also contribute to the observed evolutionary heterogeneity.

In natural *Plasmodium* populations, repeated bottlenecks, including those occurring during host-to-vector and vector-to-host transmission, can contribute to unusual allele frequency patterns^52^. A notable example of this observation was in Jinja where the PfDHFR I164L allele underwent a rapid increase followed by a major decline, and then rose again. This fluctuation coincided with IBD network clusters composed predominantly of either PfDHFR I164L mutant or wild-type parasites from the same sampling period, suggesting that resistance evolution at some sites is influenced by both drug pressure and random genetic drift resulting from transmission-related bottlenecks^53^. In malaria-endemic settings, spatial and temporal variations in malaria-transmission intensity^8,43^, driven by control interventions and natural environmental factors, can further create opportunities for bottleneck events. Under such circumstances, stochastic expansion of parasite lineages following founder events may alter the frequency of PfDHFR I164L in unpredictable ways.

Differences in the evolutionary trajectories of folate pathway mutations, PfDHPS A581G and PfDHFR I164L, further highlight a heterogeneous adaptive landscape. While PfDHPS A581G showed weaker and predominantly negative association with high relatedness, PfDHFR I164L displayed a strong association with genetically related parasite clusters, suggesting distinct population dynamics for these resistance determinants. The limited contribution of PfDHPS A581G in the combined genotype model indicates that the observed expansion of highly related resistant parasites is largely attributable to PfDHFR I164L rather than linked PfDHPS variation. This contrast is consistent with differential evolution within the folate biosynthesis pathway, where PfDHFR variants may remain more sensitive to residual antifolate exposure, whereas PfDHPS-associated resistance may be shaped by different fitness constraints and compensatory mechanisms^54,55^. In such scenarios, low-level drug exposure may suffice to maintain and gradually select for the PfDHFR I164L mutation. Additionally, compensatory mutations have been shown to reduce fitness costs associated with PfDHFR variants^8^, and thus may also facilitate its persistence and spread.

In a broader context, these findings are consistent with contrasts observed between low and high malaria-transmission settings. In low transmission regions, such as Southeast Asia, drug resistance is often associated with hard selective sweeps and reduced genetic diversity^56,57^. By contrast, in high-transmission settings such as most of Africa, frequent recombination and gene flow between parasite populations may interact with spatially variable drug selection pressure to generate more complex patterns of evolution^45,58^. Under these conditions, when drug pressure is minimal, selection may be masked by recombination and remain difficult to detect. Our results extend this understanding by highlighting substantial within-country heterogeneity in the evolutionary dynamics of the PfDHFR I164L mutation.

Overall, the data are consistent with a model in which antifolate resistance evolution in Uganda reflects more than a single emergency, which are under a spatially heterogeneous evolutionary process, including selection acting on standing and recombined PfDHFR I164L variants, with evidence of multiple local expansions across diverse genetic backgrounds. These processes are likely influenced by recombination, spatial variation in transmission intensity, and heterogeneous drug exposure, with genetic drift and local demographic processes contributing to short-term fluctuations. Together, these findings highlight the importance of considering the depth of spatial scale when interpreting genomic signatures of antimalarial drug resistance evolution, and underscore the need for sustained, fine-scale genomic surveillance.

This study is limited by uneven spatiotemporal sampling, which may underrepresent certain population substructures. Nonetheless, the large dataset from 4725 samples over six years, including over 3,500 analysed for genetic background, offers important insights into antifolate resistance dynamics in Uganda. Collectively, the results demonstrate continued persistence and spatially heterogeneous expansion of highly antifolate-resistant parasites despite reduced SP use, underlining the importance of continued genomic monitoring to inform malaria control strategies.

## Methods

### Study samples

Patients diagnosed with *P. falciparum* malaria by rapid diagnostic test or microscopy at 10 Ugandan public health facilities in 2016–2017 and 16 facilities in 2018–2022 were enrolled. Approximately 40 µL of blood was spotted onto 3MM Whatman filter paper and transported to the Infectious Diseases Research Collaboration molecular laboratory for processing. Samples were collected annually from each healthcare facility during the peak malaria season (June-July). In 2016–2017, up to 50 participants per facility were enrolled, increasing to up to 100 participants per facility in 2018–2022. During each sampling period, two to five participants were randomly sampled per day until the target sample size for that facility was reached. Informed consent was obtained from all participants prior to their inclusion in the study. Participants did not receive financial compensation for their participation.

### Targeted deep sequencing and variant calling

Genomic DNA was isolated using a Tween-Chelex-100 protocol^59^. Samples were genotyped using MIP capture and next-generation Illumina deep sequencing^24^. We used three MIP panels targeting (i) genes associated with antimalarial drug resistance, (ii) 100-kb regions flanking *pfdhfr* and *pfdhps*, and (iii) single-nucleotide polymorphisms (SNPs) distributed throughout the *P. falciparum* genome^24,60^. The captured 100-kb *pfdhfr* flanking region was used as the sequencing design window, while a 50-kb region flanking the *pfdhfr* gene was defined as a haplotype analysis window. Genome-wide SNPs were used to estimate pairwise IBD between parasite pairs.

The library construction steps include hybridisation of probes to target regions, polymerase-driven extension, gap ligation, and exonuclease digestion to enrich for circular captures, as previously described^24^. Circular captures were then amplified using Illumina-specific indexing primers and pooled for sequencing on the Illumina platform. The SNPs were selected to provide comprehensive coverage of drug resistance loci and genetic variation across the *P. falciparum* genome^24,60,61^. In addition to known resistance-associated SNPs, we included SNPs from highly polymorphic regions of the genome, which could contribute to understanding the genetic diversity and population structure of *P. falciparum*.

### Genotyping for SNPs associated with antimalarial drug resistance

Processing of demultiplexed FASTQ files was performed using the MIPTools package (https://github.com/bailey-lab/MIPTools, v0.4.0), as previously described^24,60^. For drug resistance analysis, genotypes were called per locus and sample using unique molecular identifier (UMI)-based allele depths, which provide a robust measure of independent DNA molecules and reduce false positives from PCR or sequencing errors. Reference and alternative alleles were evaluated independently and considered present if supported by at least five UMIs. Genotypes were classified as reference-only or alternative-only when a single allele met the predefined thresholds, and as mixed when both alleles met these criteria. Loci failing to meet coverage thresholds were recorded as missing.

### Testing for longitudinal prevalence trends for resistance-associated alleles

To assess temporal trends in the prevalence of the PfDHFR I164L and PfDHPS A581G mutations, we fitted site-specific linear regression models, with year as the independent variable and prevalence as the outcome. Models were fitted separately for each site to allow for heterogeneous temporal patterns across locations. For each site, a *p*-value corresponding to the slope term (year) was extracted to test for evidence of a linear temporal trend. To account for multiple comparisons across sites, *p*-values were adjusted using the Bonferroni correction. Adjusted *p*-values were reported and displayed for each site.

### Major allele inference across samples

The Variant Call File (VCF) was first processed using bcftools (v1.17) to obtain allele read depths (AD). Sample identifiers were extracted directly from the VCF header, with AD fields for all sites. For each sample and allele, within-sample allele frequencies (WSAFs) were calculated by normalising allele-specific read depths to total read depth. Genotypes were assigned as reference or alternate when WSAF was greater than or equal to 0.75 (major allele). Genomic positions without a clear major allele (maximum WSAF ≤ 0.75) were classified as ambiguous and coded as missing. The major-allele matrix was exported as a tab-delimited file, filtered for missingness before using it for downstream population genetic and IBD analyses.

### Pair-wise IBD calculation

IBD was inferred using hmmIBD-rs^33,62^, a Rust-based implementation of the hmmIBD algorithm optimised for large-scale genomic datasets. The filtered major allele matrix was provided as input, and analysis was performed with default transition model parameters, including the assumed time to most recent common ancestor (TMRCA) distribution and a recombination rate of 6.67 × 10⁻⁷ per base per generation, the estimated *P. falciparum* genome-wide recombination rate^44^. Pairwise IBD inference was parallelised using parameterised execution mode, with the allele matrix partitioned into genomic chunks of 10 SNPs to improve computational efficiency while preserving hidden Markov model continuity. Output consisted of pairwise IBD segment calls and genome-wide IBD fractions, which were subsequently used for IBD statistics and IBD network analysis.

### IBD statistics and network analysis

To investigate resistance allele-associated relatedness, pairwise IBD was estimated for ~3500 parasite samples genotyped at ~1500 SNPs. Analyses were restricted to pairs with complete PfDHFR I164L and PfDHPS A581G genotype data (>3 million pairs). Parasite pairs were classified as highly related (IBD > 0.5) or unrelated (IBD < 0.1), and their distributions (mutant-mutant, wildtype-wildtype, mutant-wildtype) were compared using chi-square tests of independence. Moderate IBD values (0.1–0.5) were excluded due to increased estimation uncertainty from sparse SNP density. Spatial heterogeneity in genotype-associated IBD patterns was assessed through site-specific analyses of highly related and unrelated pair distributions. Spatial connectivity was further evaluated by correlating pairwise IBD with geographic distance.

Bayesian generalised linear mixed-effects models were fitted in R (v4.3.1) using *brms* (v2.22.0) with the *cmdstanr* backend (v0.8.0). High identity-by-descent (IBD > 0.5) was modelled as a binary response with a Bernoulli distribution and logit link. Fixed effects included PfDHFR I164L and PfDHPS A581G genotype pairing and standardised average complexity of infection, with sampling site modelled as a random intercept. Weakly informative priors were specified: Normal(0, 2) for regression coefficients, Normal(−6, 2) for the intercept to reflect the low baseline probability of high IBD (~0.2%), and Student-t(3, 0, 2) for the site-level random-effect standard deviation. Posterior distributions were estimated using Hamiltonian Monte Carlo with the No-U-Turn Sampler (NUTS). Four chains were run for 1,000 iterations each, including 500 warm-up iterations, yielding 2000 post-warm-up samples. Sampling used adapt_delta = 0.95 and max_treedepth = 10 to reduce divergent transitions. Convergence was assessed using R̂, effective sample sizes (ESS), and trace plots (Supplementary Fig. 3). Posterior estimates are reported as medians with 95% credible intervals.

Structure was examined using IBD network analyses, applying a stringent threshold (IBD ≥ 0.9) to define highly connected clusters and a lower threshold (IBD ≥ 0.5) to assess broader connectivity across sites, collection years, and genotypes. Networks were identified using community detection algorithms implemented in igraph (version 2.1.4), with each isolate assigned to a cluster. For each cluster, network metrics, including size, average degree and strength, internal and external edge counts, and internal edge density, were calculated.

### Analysis for haplotypes flanking the pfdhfr gene

Genotype data was extracted for chromosome 4 from the VCF file obtained from MIPs targeting regions flanking the *pfdhfr* gene and recoded into a numeric format, where 0 indicates homozygous reference^2^, homozygous, 1 any heterozygous genotype, and −1 missing data; Reference and alternative allele definitions were preserved throughout. A 50-kb and 10-kb windows flanking *pfdhfr* gene, centred on the core position at 748,576 bp of chromosome 4, were used to investigate haplotype sharing and structure. Haplotype structure was visualised using heatmaps generated with the complexHeatmap (v2.18.0) R package (v4.3.1), with hierarchical clustering of samples and discrete colour schemes for metadata categories.

### Signals of recent positive selection

We used the R package *rehh* (v3.2.2) to scan for signals of recent selection in genomic regions flanking the *pfdhfr* gene using EHH statistics^51,63^. Allele-specific EHH decay was calculated for isolates from eight sites with PfDHFR I164L prevalence greater than 10% and at least four isolates carrying the allele. Haplotype homozygosity statistics were calculated with PfDHFR I164L as the core allele, and the patterns of EHH decay were visualised in an EHH decay plot.

### Ethics statement

This study was approved by the Makerere University School of Biomedical Sciences Research Ethics Committee, the Uganda National Council for Science and Technology and the University of California, San Francisco, Human Research Protection Program. Written Informed consent was obtained from all study participants before enrolment.

### Reporting summary

Further information on research design is available in the Nature Portfolio Reporting Summary linked to this article.

## Supplementary information

Supplementary Information

Description of Additional Supplementary Files

Supplementary Data 1

Reporting Summary

Transparent Peer Review file

## Source data

Source Data

## Data Availability

The raw sequence reads generated in this study have been deposited in the National Center for Biotechnology Information (NCBI) Sequence Read Archive (SRA) under the following accession numbers: PRJNA880926, PRJNA880930, PRJNA655702, PRJNA880932, and PRJNA880933. The processed data are available at https://github.com/victorasua/Antifolate_Resistance_Evolution/tree/main/Raw_Data. Source data are provided with this paper.

## Code availability

Analyses and figure generation were performed using standard R packages. Details are provided in the Reporting Summary. No custom code was used. The codes used to generate figures are available at https://github.com/victorasua/Antifolate_Resistance_Evolution/tree/main/codes.
