# Supplementary material for: Multiple local PfDHFR I164L haplotype expansions drive Plasmodium falciparum antifolate resistance in Uganda
Source: Nat Commun. 2026 Sep 4;17:9552. doi: 10.1038/s41467-026-76826-4 (PMC13550557; doi:10.1038/s41467-026-76826-4)
Supplement: Supplementary file 1 [file 41467_2026_76826_MOESM1_ESM.pdf]

Multiple local PfDHFR I164L haplotype expansions drive *Plasmodium falciparum* antifolate resistance in Uganda

Victor Asua[1,2,3]*, Karamoko Niaré[4,5], Shreeya Garg[6], Jenny Legac[6], Stephen Tukwasibwe[2,7], Thomas Katairo[2], Francis E. Bohissou[1,8,9], Rebecca M. Crudale[4], Alfred Simkin[4], Jerry Mulondo[2], Sam L. Nsobya[2,7], Isaac Ssewanyana[2], Grant Dorsey[6], Jeffrey A. Bailey[4,10], Moses R. Kamya[2,7], Adoke Yeka[2,7], Philip J. Rosenthal[6], Steffen Borrmann[1,3 †], Melissa D. Conrad[6,11 †]

Institutions and affiliations

[1] Institute for Tropical Medicine, University of Tübingen, Tübingen, Germany

[2] Infectious Diseases Research Collaboration, Kampala, Uganda

[3] German Centre for Infection Research, Partner site Tübingen, Germany

[4] Department of Pathology and Laboratory Medicine, Brown University, Providence, RI, USA

[5] Pathogens Genomics Diversity Network Africa, Imm. Gwancoura, Sotuba, Bamako, Mali

[6] University of California San Francisco, San Francisco, CA, USA

[7] Makerere University, Kampala, Uganda

[8] Institut de Recherche en Sciences de la Santé /Clinical Research Unit of Nanoro, Nanoro, Burkina Faso

[9] Centre de Recherche Entomologique de Cotonou, Cotonou, Bénin

[10] Centre for Computational Molecular Biology, Brown University, Providence, RI, USA

[11] Bloomberg School of Public Health, Johns Hopkins University, Baltimore, MD, USA

† **Joint supervision**

***Corresponding author**

Victor Asua
Institute for Tropical Medicine, University of Tübingen, Wilhelmstraße 27, 72074, Tübingen, Germany
Email: victorasua@yahoo.co.uk

Keywords: *Plasmodium falciparum*, drug, antifolate, sulfadoxine, pyrimethamine, resistance, evolution, Uganda

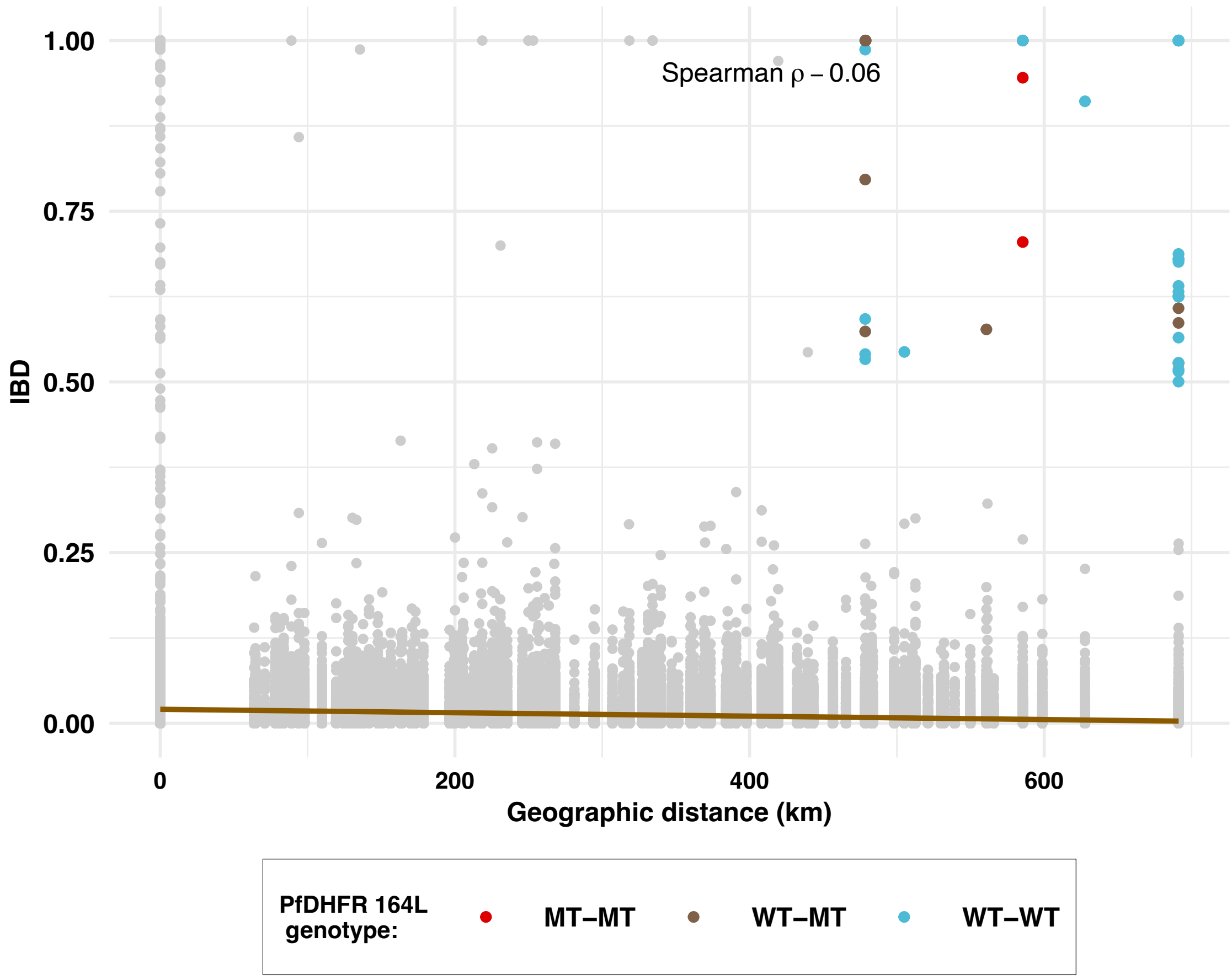

Supplementary Figure 1. Relationship between IBD and geographic distance. A subset of 50,000 parasite pairs (grey points) was used to assess the relationship between IBD and geographic distance, with a linear regression (orange line) showing the trend. High-IBD long-distance outliers (IBD > 0.5, distance > 450 km) are coloured by PfDHFR I164L genotype (WT-WT, MT-MT, WT-MT). The Spearman correlation (rho = -0.06) shows a weak relationship between IBD and geographic distance. Outliers indicate long-range transmission and shared ancestry despite geographic separation

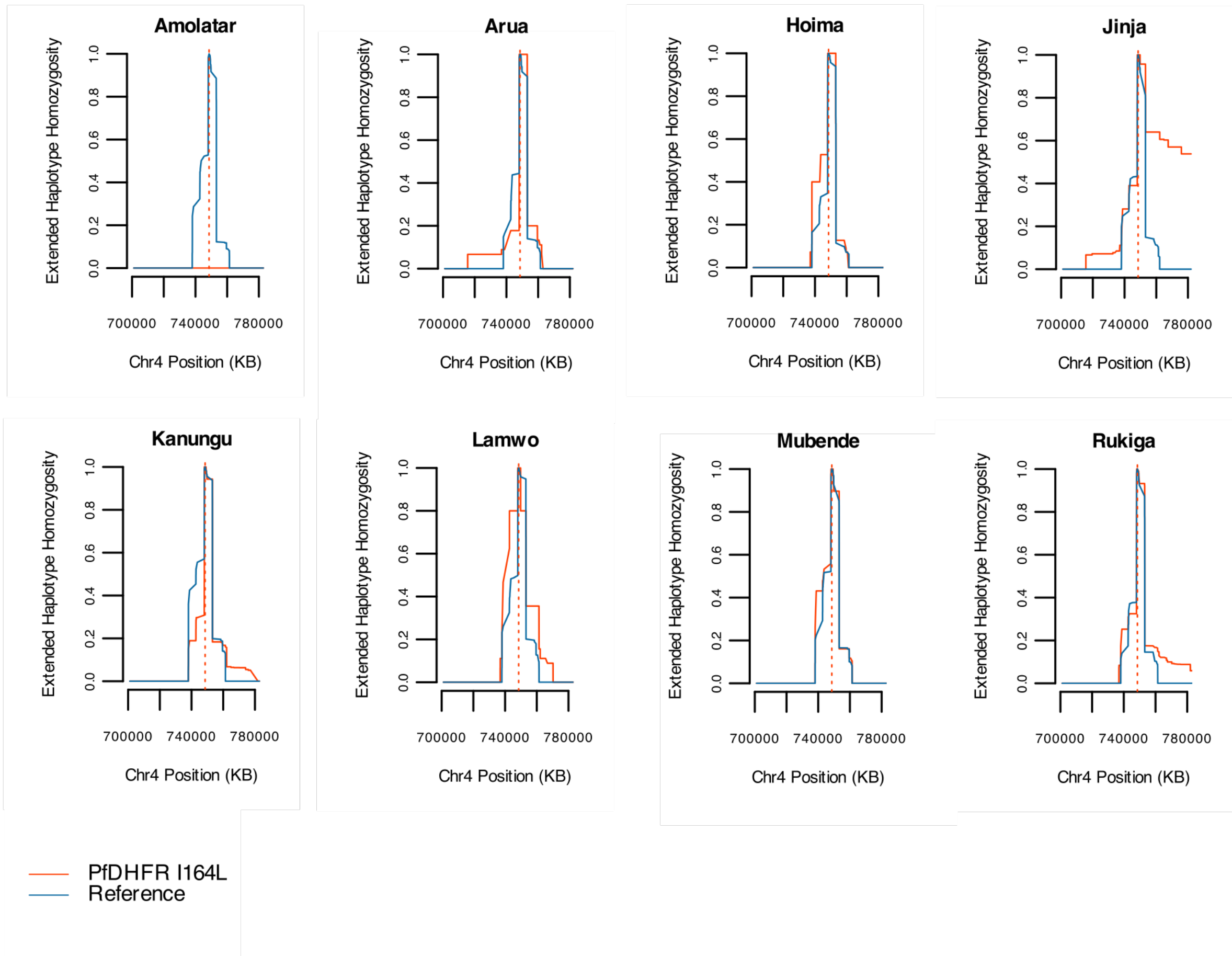

Supplementary Figure 2. Extended haplotype homozygosity (EHH) decay. Extended haplotype homozygosity (EHH) decay with PfDHFR I164L as the core allele. The x-axis represents increasing genetic distance from the core locus. The vertical dotted line indicates the position of the core allele.

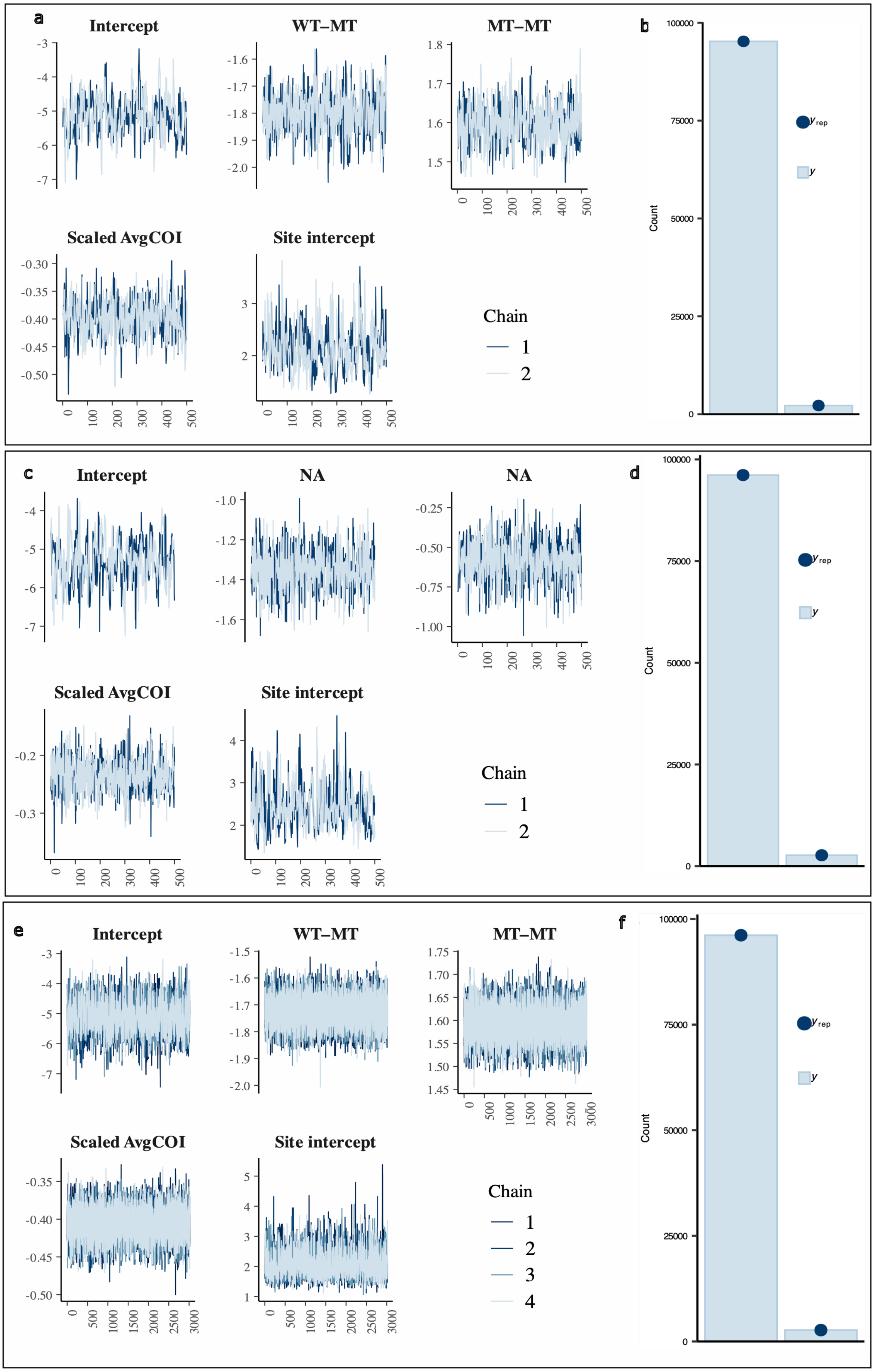

a
Intercept
WT–MT
MT–MT
Scaled AvgCOI
Site intercept
Chain
1
2
b
Count
100000
75000
50000
25000
0
y_{rep}
y
c
Intercept
NA
NA
Scaled AvgCOI
Site intercept
Chain
1
2
d
Count
100000
75000
50000
25000
0
y_{rep}
y
e
Intercept
WT–MT
MT–MT
Scaled AvgCOI
Site intercept
Chain
1
2
3
4
f
Count
100000
75000
50000
25000
0
y_{rep}
y

Supplementary Figure 3. Bayesian model validation and convergence diagnostics. (a, c, e) Markov chain Monte Carlo (MCMC) trace plots. Two Hamiltonian Monte Carlo chains were run for 1,000 iterations each, including 500 warm-up iterations, yielding 1,000 post-warm-up draws. All parameters demonstrated satisfactory convergence ($\hat{R}$ = 1.00–1.02). Bulk effective sample sizes (ESS) exceeded 350 for all fixed-effect parameters, while Tail ESS values were greater than 150, indicating adequate estimation of the posterior distributions. (b, d, f) Posterior predictive checks comparing the observed distribution of high identity-by-descent (IBD) with replicated datasets generated from the fitted Bayesian generalized linear mixed-effects models showed good agreement between the observed and simulated data, indicating good model fit. Models were fitted separately for PfDHFR I164L pairs (a, b), PfDHPS A581G pairs (c, d), and combined I164L/A581G pairs (e, f).

Supplementary Table 1. Spatial and temporal prevalence of antifolate resistance-associated mutations across Uganda

Site	Gene	Loci	Proportion mutant (95% CI), N				
			2016-17	2018-19	2020	2021	2022
Agago	PfDHFR	A16V	0.00 (0.00 - 0.08), 53	0.00 (0.00 - 0.12), 36	0.00 (0.00 - 0.09), 50	0.00 (0.00 - 0.09), 52	0.00 (0.00 - 0.06), 75
		N51I	1.00 (0.92 - 1.00), 58	1.00 (0.89 - 1.00), 38	1.00 (0.92 - 1.00), 54	1.00 (0.92 - 1.00), 54	0.98 (0.91 - 1.00), 81
		C59R	0.93 (0.82 - 0.98), 58	0.97 (0.85 - 1.00), 38	0.94 (0.84 - 0.99), 54	0.96 (0.86 - 0.99), 54	0.94 (0.86 - 0.98), 81
		S108N	1.00 (0.91 - 1.00), 51	1.00 (0.89 - 1.00), 39	1.00 (0.91 - 1.00), 52	1.00 (0.91 - 1.00), 49	0.97 (0.90 - 1.00), 74
		I164L	0.06 (0.02 - 0.18), 50	0.03 (0.00 - 0.15), 38	0.06 (0.02 - 0.17), 51	0.00 (0.00 - 0.10), 44	0.06 (0.02 - 0.15), 71
	PfDHPS	I431V	0.00 (0.00 - 0.09), 50	0.00 (0.00 - 0.12), 36	0.00 (0.00 - 0.08), 54	0.00 (0.00 - 0.08), 53	0.00 (0.00 - 0.06), 75
		S436A	0.02 (0.00 - 0.13), 46	0.00 (0.00 - 0.12), 36	0.00 (0.00 - 0.09), 51	0.00 (0.00 - 0.08), 53	0.00 (0.00 - 0.06), 70
		A437G	0.92 (0.80 - 0.97), 50	0.97 (0.84 - 1.00), 36	0.94 (0.84 - 0.99), 54	0.96 (0.86 - 0.99), 53	0.93 (0.84 - 0.98), 75
		K540E	0.96 (0.87 - 0.99), 56	0.97 (0.84 - 1.00), 36	0.93 (0.81 - 0.98), 54	0.98 (0.89 - 1.00), 57	0.94 (0.85 - 0.98), 79
		A581G	0.07 (0.02 - 0.17), 59	0.03 (0.00 - 0.15), 39	0.00 (0.00 - 0.08), 53	0.00 (0.00 - 0.07), 63	0.04 (0.01 - 0.12), 78
		A613S	0.00 (0.00 - 0.08), 54	0.00 (0.00 - 0.11), 39	0.00 (0.00 - 0.09), 52	0.00 (0.00 - 0.07), 60	0.00 (0.00 - 0.06), 76
Amolatar	PfDHFR	A16V	0.00 (0.00 - 0.08), 55	0.00 (0.00 - 0.28), 13	0.00 (0.00 - 0.10), 45	0.00 (0.00 - 0.09), 52	0.00 (0.00 - 0.30), 12
		N51I	1.00 (0.92 - 1.00), 58	1.00 (0.79 - 1.00), 19	1.00 (0.91 - 1.00), 52	1.00 (0.92 - 1.00), 53	1.00 (0.72 - 1.00), 13
		C59R	1.00 (0.92 - 1.00), 58	1.00 (0.79 - 1.00), 19	1.00 (0.91 - 1.00), 52	0.98 (0.89 - 1.00), 53	1.00 (0.72 - 1.00), 13
		S108N	1.00 (0.92 - 1.00), 53	1.00 (0.76 - 1.00), 16	1.00 (0.90 - 1.00), 44	1.00 (0.91 - 1.00), 47	1.00 (0.72 - 1.00), 13
		I164L	0.12 (0.05 - 0.25), 51	0.24 (0.08 - 0.50), 17	0.09 (0.03 - 0.22), 46	0.09 (0.02 - 0.24), 35	1.00 (0.60 - 1.00), 8
	PfDHPS	I431V	0.00 (0.00 - 0.09), 52	0.00 (0.00 - 0.32), 11	0.00 (0.00 - 0.10), 44	0.00 (0.00 - 0.08), 54	0.00 (0.00 - 0.30), 12
		S436A	0.00 (0.00 - 0.09), 51	0.00 (0.00 - 0.32), 11	0.00 (0.00 - 0.10), 44	0.00 (0.00 - 0.08), 54	0.00 (0.00 - 0.30), 12
		A437G	0.98 (0.88 - 1.00), 52	1.00 (0.68 - 1.00), 11	1.00 (0.90 - 1.00), 44	1.00 (0.92 - 1.00), 54	1.00 (0.70 - 1.00), 12
		K540E	0.98 (0.89 - 1.00), 56	1.00 (0.56 - 1.00), 7	1.00 (0.91 - 1.00), 48	1.00 (0.92 - 1.00), 54	1.00 (0.72 - 1.00), 13
		A581G	0.08 (0.03 - 0.19), 60	0.00 (0.00 - 0.27), 14	0.00 (0.00 - 0.09), 52	0.00 (0.00 - 0.08), 58	0.00 (0.00 - 0.25), 15
		A613S	0.00 (0.00 - 0.08), 54	0.00 (0.00 - 0.28), 13	0.00 (0.00 - 0.10), 46	0.00 (0.00 - 0.08), 55	0.00 (0.00 - 0.25), 15
Arua	PfDHFR	A16V	0.00 (0.00 - 0.08), 59	0.00 (0.00 - 0.14), 30	0.00 (0.00 - 0.09), 47	0.00 (0.00 - 0.07), 67	0.00 (0.00 - 0.06), 78
		N51I	1.00 (0.93 - 1.00), 62	1.00 (0.87 - 1.00), 33	1.00 (0.91 - 1.00), 50	1.00 (0.94 - 1.00), 70	1.00 (0.94 - 1.00), 79
		C59R	0.92 (0.81 - 0.97), 62	0.91 (0.75 - 0.98), 33	0.86 (0.73 - 0.94), 50	0.93 (0.83 - 0.97), 70	0.92 (0.84 - 0.97), 79
		S108N	1.00 (0.93 - 1.00), 62	1.00 (0.87 - 1.00), 33	1.00 (0.91 - 1.00), 49	1.00 (0.93 - 1.00), 61	1.00 (0.94 - 1.00), 77
		I164L	0.10 (0.04 - 0.21), 60	0.03 (0.00 - 0.17), 34	0.06 (0.02 - 0.18), 49	0.07 (0.02 - 0.17), 59	0.20 (0.12 - 0.32), 69
	PfDHPS	I431V	0.00 (0.00 - 0.07), 62	0.00 (0.00 - 0.15), 29	0.00 (0.00 - 0.09), 49	0.00 (0.00 - 0.07), 69	0.00 (0.00 - 0.06), 76
		S436A	0.08 (0.03 - 0.19), 61	0.10 (0.03 - 0.28), 29	0.06 (0.02 - 0.18), 49	0.06 (0.02 - 0.15), 69	0.00 (0.00 - 0.06), 76
		A437G	0.98 (0.90 - 1.00), 62	0.97 (0.80 - 1.00), 29	1.00 (0.91 - 1.00), 49	0.97 (0.89 - 0.99), 69	0.99 (0.92 - 1.00), 76
		K540E	0.98 (0.91 - 1.00), 65	0.94 (0.77 - 0.99), 31	1.00 (0.91 - 1.00), 50	0.97 (0.90 - 1.00), 73	0.97 (0.90 - 1.00), 78
		A581G	0.15 (0.08 - 0.27), 65	0.09 (0.02 - 0.25), 33	0.14 (0.06 - 0.27), 50	0.20 (0.12 - 0.32), 74	0.15 (0.08 - 0.25), 81
		A613S	0.00 (0.00 - 0.07), 64	0.00 (0.00 - 0.13), 33	0.00 (0.00 - 0.09), 48	0.00 (0.00 - 0.06), 73	0.00 (0.00 - 0.06), 78
Hoima	PfDHFR	A16V	-	0.00 (0.00 - 0.06), 72	0.00 (0.00 - 0.06), 81	0.00 (0.00 - 0.06), 82	0.00 (0.00 - 0.07), 61
		N51I	-	1.00 (0.94 - 1.00), 75	1.00 (0.94 - 1.00), 83	1.00 (0.94 - 1.00), 83	1.00 (0.93 - 1.00), 66
		C59R	-	0.96 (0.88 - 0.99), 75	0.92 (0.83 - 0.96), 83	1.00 (0.94 - 1.00), 83	1.00 (0.93 - 1.00), 66

		S108N	-	1.00 (0.94 - 1.00), 73	1.00 (0.94 - 1.00), 81	1.00 (0.94 - 1.00), 70	1.00 (0.92 - 1.00), 58
		I164L	-	0.21 (0.13 - 0.33), 70	0.21 (0.13 - 0.32), 80	0.09 (0.04 - 0.21), 53	0.20 (0.11 - 0.34), 50
	PfDHPS	I431V	-	0.00 (0.00 - 0.06), 72	0.00 (0.00 - 0.06), 82	0.00 (0.00 - 0.06), 82	0.00 (0.00 - 0.08), 59
		S436A	-	0.03 (0.00 - 0.11), 72	0.00 (0.00 - 0.06), 82	0.00 (0.00 - 0.06), 78	0.00 (0.00 - 0.08), 59
		A437G	-	1.00 (0.94 - 1.00), 72	1.00 (0.94 - 1.00), 82	0.95 (0.87 - 0.98), 82	1.00 (0.92 - 1.00), 59
		K540E	-	1.00 (0.93 - 1.00), 64	1.00 (0.94 - 1.00), 81	0.96 (0.88 - 0.99), 89	1.00 (0.93 - 1.00), 63
		A581G	-	0.14 (0.08 - 0.25), 76	0.10 (0.05 - 0.19), 81	0.22 (0.14 - 0.32), 92	0.00 (0.00 - 0.07), 68
		A613S	-	0.00 (0.00 - 0.06), 75	0.00 (0.00 - 0.06), 81	0.00 (0.00 - 0.05), 88	0.00 (0.00 - 0.07), 64
Jinja	PfDHFR	A16V	0.00 (0.00 - 0.08), 54	0.00 (0.00 - 0.06), 79	0.00 (0.00 - 0.05), 86	0.00 (0.00 - 0.05), 92	0.00 (0.00 - 0.03), 156
		N51I	1.00 (0.92 - 1.00), 56	1.00 (0.94 - 1.00), 82	1.00 (0.95 - 1.00), 92	1.00 (0.95 - 1.00), 95	1.00 (0.97 - 1.00), 157
		C59R	0.98 (0.89 - 1.00), 56	0.99 (0.92 - 1.00), 82	1.00 (0.95 - 1.00), 92	1.00 (0.95 - 1.00), 95	1.00 (0.97 - 1.00), 157
		S108N	1.00 (0.91 - 1.00), 47	1.00 (0.94 - 1.00), 81	1.00 (0.95 - 1.00), 88	1.00 (0.95 - 1.00), 87	1.00 (0.97 - 1.00), 152
		I164L	0.28 (0.16 - 0.44), 43	0.26 (0.17 - 0.37), 82	0.56 (0.45 - 0.66), 90	0.01 (0.00 - 0.08), 80	0.84 (0.77 - 0.89), 144
	PfDHPS	I431V	0.00 (0.00 - 0.08), 59	0.00 (0.00 - 0.06), 74	0.00 (0.00 - 0.05), 85	0.00 (0.00 - 0.05), 93	0.00 (0.00 - 0.03), 160
		S436A	0.00 (0.00 - 0.08), 58	0.04 (0.01 - 0.12), 74	0.00 (0.00 - 0.05), 85	0.00 (0.00 - 0.05), 93	0.00 (0.00 - 0.03), 160
		A437G	1.00 (0.92 - 1.00), 58	1.00 (0.94 - 1.00), 74	1.00 (0.95 - 1.00), 85	1.00 (0.95 - 1.00), 93	1.00 (0.97 - 1.00), 160
		K540E	1.00 (0.92 - 1.00), 56	1.00 (0.94 - 1.00), 73	1.00 (0.95 - 1.00), 93	1.00 (0.95 - 1.00), 96	1.00 (0.97 - 1.00), 159
		A581G	0.05 (0.01 - 0.15), 61	0.02 (0.00 - 0.09), 82	0.17 (0.08 - 0.31), 47	0.01 (0.00 - 0.07), 95	0.00 (0.00 - 0.03), 166
		A613S	0.00 (0.00 - 0.07), 60	0.00 (0.00 - 0.06), 82	0.00 (0.00 - 0.05), 86	0.00 (0.00 - 0.05), 94	0.00 (0.00 - 0.03), 164
Kaabong	PfDHFR	A16V	-	0.00 (0.00 - 0.11), 39	0.00 (0.00 - 0.11), 41	0.00 (0.00 - 0.08), 54	0.00 (0.00 - 0.06), 73
		N51I	-	1.00 (0.89 - 1.00), 39	0.98 (0.86 - 1.00), 42	0.97 (0.88 - 0.99), 61	1.00 (0.94 - 1.00), 83
		C59R	-	0.90 (0.75 - 0.97), 39	0.93 (0.79 - 0.98), 42	0.90 (0.79 - 0.96), 61	0.96 (0.89 - 0.99), 83
		S108N	-	1.00 (0.89 - 1.00), 39	0.97 (0.85 - 1.00), 38	0.98 (0.88 - 1.00), 50	0.99 (0.92 - 1.00), 74
		I164L	-	0.00 (0.00 - 0.11), 40	0.00 (0.00 - 0.11), 40	0.02 (0.00 - 0.13), 46	0.03 (0.00 - 0.11), 70
	PfDHPS	I431V	-	0.00 (0.00 - 0.12), 36	0.00 (0.00 - 0.11), 40	0.00 (0.00 - 0.08), 58	0.00 (0.00 - 0.06), 70
		S436A	-	0.03 (0.00 - 0.18), 32	0.05 (0.01 - 0.19), 38	0.04 (0.01 - 0.14), 52	0.00 (0.00 - 0.07), 66
		A437G	-	0.89 (0.73 - 0.96), 36	0.95 (0.82 - 0.99), 40	0.90 (0.78 - 0.96), 58	0.91 (0.82 - 0.96), 70
		K540E	-	0.89 (0.74 - 0.96), 37	0.93 (0.80 - 0.98), 43	0.89 (0.78 - 0.95), 63	0.92 (0.83 - 0.97), 77
		A581G	-	0.00 (0.00 - 0.11), 39	0.00 (0.00 - 0.10), 44	0.08 (0.03 - 0.18), 66	0.00 (0.00 - 0.06), 78
		A613S	-	0.00 (0.00 - 0.11), 39	0.00 (0.00 - 0.11), 40	0.00 (0.00 - 0.07), 64	0.00 (0.00 - 0.06), 74
Kabale	PfDHFR	A16V	0.00 (0.00 - 0.11), 39	0.00 (0.00 - 0.37), 9	0.00 (0.00 - 0.14), 30	0.00 (0.00 - 0.06), 80	0.00 (0.00 - 0.08), 58
		N51I	1.00 (0.89 - 1.00), 40	1.00 (0.66 - 1.00), 10	1.00 (0.86 - 1.00), 31	1.00 (0.95 - 1.00), 85	1.00 (0.93 - 1.00), 63
		C59R	0.95 (0.82 - 0.99), 40	1.00 (0.66 - 1.00), 10	0.90 (0.73 - 0.97), 31	0.96 (0.89 - 0.99), 85	0.97 (0.88 - 0.99), 63
		S108N	1.00 (0.89 - 1.00), 38	1.00 (0.68 - 1.00), 11	1.00 (0.84 - 1.00), 27	1.00 (0.94 - 1.00), 80	1.00 (0.92 - 1.00), 59
		I164L	0.22 (0.10 - 0.39), 37	0.33 (0.11 - 0.65), 12	0.36 (0.19 - 0.56), 28	0.61 (0.49 - 0.72), 72	0.76 (0.62 - 0.86), 54
	PfDHPS	I431V	0.00 (0.00 - 0.11), 40	0.00 (0.00 - 0.44), 7	0.00 (0.00 - 0.15), 29	0.00 (0.00 - 0.06), 81	0.00 (0.00 - 0.08), 57
		S436A	0.00 (0.00 - 0.11), 40	0.00 (0.00 - 0.44), 7	0.00 (0.00 - 0.16), 27	0.00 (0.00 - 0.06), 81	0.00 (0.00 - 0.08), 57
		A437G	1.00 (0.89 - 1.00), 40	1.00 (0.56 - 1.00), 7	0.93 (0.76 - 0.99), 29	1.00 (0.94 - 1.00), 81	1.00 (0.92 - 1.00), 57
		K540E	1.00 (0.89 - 1.00), 39	0.86 (0.42 - 0.99), 7	0.91 (0.74 - 0.98), 32	0.98 (0.91 - 1.00), 86	1.00 (0.93 - 1.00), 62

		A581G	0.73 (0.57 - 0.85), 41	0.40 (0.14 - 0.73), 10	0.42 (0.26 - 0.61), 33	0.82 (0.72 - 0.89), 85	0.80 (0.68 - 0.89), 65
		A613S	0.00 (0.00 - 0.11), 41	0.00 (0.00 - 0.34), 10	0.00 (0.00 - 0.15), 28	0.00 (0.00 - 0.06), 82	0.00 (0.00 - 0.07), 60
Kanungu	PfDHFR	A16V	0.00 (0.00 - 0.06), 71	0.00 (0.00 - 0.09), 52	0.00 (0.00 - 0.06), 77	0.00 (0.00 - 0.10), 46	0.00 (0.00 - 0.07), 66
		N51I	0.99 (0.92 - 1.00), 78	1.00 (0.92 - 1.00), 53	1.00 (0.94 - 1.00), 81	1.00 (0.91 - 1.00), 48	1.00 (0.94 - 1.00), 71
		C59R	0.99 (0.92 - 1.00), 78	0.98 (0.89 - 1.00), 53	0.94 (0.86 - 0.98), 81	0.98 (0.88 - 1.00), 48	0.99 (0.91 - 1.00), 71
		S108N	1.00 (0.94 - 1.00), 71	1.00 (0.92 - 1.00), 56	1.00 (0.94 - 1.00), 80	1.00 (0.86 - 1.00), 31	1.00 (0.93 - 1.00), 64
		I164L	0.30 (0.20 - 0.43), 66	0.44 (0.31 - 0.58), 57	0.49 (0.37 - 0.60), 78	0.41 (0.19 - 0.67), 17	0.64 (0.50 - 0.77), 53
	PfDHPS	I431V	0.00 (0.00 - 0.06), 76	0.00 (0.00 - 0.08), 53	0.00 (0.00 - 0.06), 79	0.00 (0.00 - 0.08), 53	0.00 (0.00 - 0.07), 66
		S436A	0.00 (0.00 - 0.06), 76	0.00 (0.00 - 0.09), 52	0.00 (0.00 - 0.06), 79	0.00 (0.00 - 0.08), 53	0.00 (0.00 - 0.08), 57
		A437G	1.00 (0.94 - 1.00), 76	0.98 (0.89 - 1.00), 53	1.00 (0.94 - 1.00), 79	1.00 (0.92 - 1.00), 53	0.86 (0.75 - 0.93), 66
		K540E	1.00 (0.94 - 1.00), 78	0.92 (0.81 - 0.98), 52	1.00 (0.94 - 1.00), 80	1.00 (0.92 - 1.00), 59	0.86 (0.75 - 0.93), 72
		A581G	0.57 (0.45 - 0.67), 83	0.43 (0.31 - 0.57), 60	0.38 (0.28 - 0.50), 81	0.47 (0.34 - 0.60), 62	0.36 (0.25 - 0.48), 75
		A613S	0.00 (0.00 - 0.06), 79	0.00 (0.00 - 0.07), 60	0.00 (0.00 - 0.06), 81	0.00 (0.00 - 0.07), 61	0.00 (0.00 - 0.07), 68
Kapchorwa	PfDHFR	A16V	-	0.00 (0.00 - 0.34), 10	-	0.00 (0.00 - 0.06), 73	0.00 (0.00 - 0.17), 25
		N51I	-	1.00 (0.70 - 1.00), 12	-	1.00 (0.94 - 1.00), 75	1.00 (0.85 - 1.00), 29
		C59R	-	1.00 (0.70 - 1.00), 12	-	0.97 (0.90 - 1.00), 75	0.38 (0.21 - 0.58), 29
		S108N	-	1.00 (0.68 - 1.00), 11	-	1.00 (0.93 - 1.00), 62	1.00 (0.84 - 1.00), 27
		I164L	-	0.00 (0.00 - 0.27), 14	-	0.00 (0.00 - 0.12), 35	0.00 (0.00 - 0.18), 22
	PfDHPS	I431V	-	0.00 (0.00 - 0.37), 9	-	0.00 (0.00 - 0.06), 79	0.00 (0.00 - 0.17), 24
		S436A	-	0.00 (0.00 - 0.37), 9	-	0.01 (0.00 - 0.08), 79	0.00 (0.00 - 0.18), 23
		A437G	-	1.00 (0.63 - 1.00), 9	-	1.00 (0.94 - 1.00), 79	0.96 (0.77 - 1.00), 24
		K540E	-	1.00 (0.52 - 1.00), 6	-	0.99 (0.93 - 1.00), 83	0.97 (0.81 - 1.00), 30
		A581G	-	0.00 (0.00 - 0.28), 13	-	0.00 (0.00 - 0.05), 85	0.00 (0.00 - 0.15), 29
		A613S	-	0.00 (0.00 - 0.28), 13	-	0.00 (0.00 - 0.05), 84	0.00 (0.00 - 0.18), 23
Kasese	PfDHFR	A16V	-	0.00 (0.00 - 0.12), 37	0.00 (0.00 - 0.06), 73	0.00 (0.00 - 0.06), 76	0.00 (0.00 - 0.04), 108
		N51I	-	1.00 (0.89 - 1.00), 39	1.00 (0.94 - 1.00), 74	1.00 (0.94 - 1.00), 76	1.00 (0.96 - 1.00), 111
		C59R	-	0.95 (0.81 - 0.99), 39	0.92 (0.83 - 0.97), 74	0.99 (0.92 - 1.00), 76	0.95 (0.89 - 0.98), 111
		S108N	-	1.00 (0.89 - 1.00), 38	1.00 (0.93 - 1.00), 69	1.00 (0.93 - 1.00), 65	1.00 (0.96 - 1.00), 105
		I164L	-	0.49 (0.33 - 0.65), 39	0.49 (0.37 - 0.61), 71	0.52 (0.39 - 0.65), 60	0.30 (0.21 - 0.40), 98
	PfDHPS	I431V	-	0.00 (0.00 - 0.12), 36	0.00 (0.00 - 0.06), 74	0.00 (0.00 - 0.06), 74	0.00 (0.00 - 0.04), 105
		S436A	-	0.00 (0.00 - 0.12), 36	0.00 (0.00 - 0.06), 73	0.00 (0.00 - 0.06), 74	0.01 (0.00 - 0.06), 103
		A437G	-	1.00 (0.88 - 1.00), 36	0.99 (0.92 - 1.00), 74	1.00 (0.94 - 1.00), 74	0.96 (0.90 - 0.99), 105
		K540E	-	0.97 (0.83 - 1.00), 34	0.95 (0.86 - 0.98), 73	1.00 (0.94 - 1.00), 81	0.98 (0.93 - 1.00), 110
		A581G	-	0.56 (0.40 - 0.71), 41	0.49 (0.38 - 0.61), 73	0.43 (0.32 - 0.54), 82	0.36 (0.27 - 0.46), 111
		A613S	-	0.00 (0.00 - 0.11), 40	0.00 (0.00 - 0.06), 70	0.00 (0.00 - 0.06), 79	0.00 (0.00 - 0.04), 107
Katakwi	PfDHFR	A16V	-	0.00 (0.00 - 0.08), 56	0.00 (0.00 - 0.09), 49	0.00 (0.00 - 0.08), 55	0.00 (0.00 - 0.06), 78
		N51I	-	1.00 (0.93 - 1.00), 60	1.00 (0.91 - 1.00), 52	0.98 (0.90 - 1.00), 59	0.99 (0.92 - 1.00), 81
		C59R	-	0.98 (0.90 - 1.00), 60	0.96 (0.86 - 0.99), 52	0.98 (0.90 - 1.00), 59	0.95 (0.87 - 0.98), 81
		S108N	-	1.00 (0.92 - 1.00), 58	1.00 (0.90 - 1.00), 44	0.98 (0.89 - 1.00), 54	0.99 (0.92 - 1.00), 74

		I164L	-	0.02 (0.00 - 0.10), 59	0.00 (0.00 - 0.09), 47	0.09 (0.03 - 0.22), 46	0.06 (0.02 - 0.16), 66
	PfDHPS	I431V	-	0.00 (0.00 - 0.09), 51	0.00 (0.00 - 0.09), 49	0.00 (0.00 - 0.08), 55	0.00 (0.00 - 0.06), 76
		S436A	-	0.02 (0.00 - 0.12), 51	0.00 (0.00 - 0.10), 45	0.00 (0.00 - 0.08), 53	0.00 (0.00 - 0.06), 71
		A437G	-	0.98 (0.88 - 1.00), 51	0.90 (0.77 - 0.96), 49	0.96 (0.86 - 0.99), 55	0.93 (0.85 - 0.98), 76
		K540E	-	0.98 (0.89 - 1.00), 53	0.93 (0.81 - 0.98), 54	0.95 (0.86 - 0.99), 62	0.95 (0.87 - 0.98), 81
		A581G	-	0.00 (0.00 - 0.07), 61	0.00 (0.00 - 0.09), 51	0.02 (0.00 - 0.10), 64	0.00 (0.00 - 0.05), 85
		A613S	-	0.00 (0.00 - 0.07), 61	0.00 (0.00 - 0.10), 44	0.00 (0.00 - 0.08), 59	0.00 (0.00 - 0.06), 81
Koboko	PfDHFR	A16V	-	0.00 (0.00 - 0.17), 25	0.00 (0.00 - 0.12), 36	0.00 (0.00 - 0.11), 38	0.00 (0.00 - 0.10), 46
		N51I	-	1.00 (0.84 - 1.00), 27	1.00 (0.89 - 1.00), 41	1.00 (0.90 - 1.00), 43	1.00 (0.92 - 1.00), 55
		C59R	-	0.85 (0.65 - 0.95), 27	0.93 (0.79 - 0.98), 41	0.84 (0.69 - 0.93), 43	0.95 (0.84 - 0.99), 55
		S108N	-	1.00 (0.83 - 1.00), 24	1.00 (0.88 - 1.00), 36	1.00 (0.88 - 1.00), 35	1.00 (0.90 - 1.00), 44
		I164L	-	0.00 (0.00 - 0.16), 27	0.00 (0.00 - 0.11), 38	0.00 (0.00 - 0.14), 30	0.00 (0.00 - 0.11), 41
	PfDHPS	I431V	-	0.00 (0.00 - 0.17), 24	0.00 (0.00 - 0.13), 34	0.00 (0.00 - 0.11), 38	0.00 (0.00 - 0.10), 46
		S436A	-	0.04 (0.00 - 0.23), 24	0.12 (0.04 - 0.28), 34	0.05 (0.01 - 0.19), 38	0.04 (0.01 - 0.16), 45
		A437G	-	1.00 (0.83 - 1.00), 24	1.00 (0.87 - 1.00), 34	1.00 (0.89 - 1.00), 38	0.96 (0.84 - 0.99), 46
		K540E	-	1.00 (0.82 - 1.00), 23	0.95 (0.83 - 0.99), 42	0.98 (0.88 - 1.00), 48	0.96 (0.85 - 0.99), 50
		A581G	-	0.15 (0.05 - 0.36), 26	0.14 (0.06 - 0.29), 43	0.10 (0.04 - 0.23), 49	0.04 (0.01 - 0.14), 52
		A613S	-	0.00 (0.00 - 0.16), 27	0.00 (0.00 - 0.12), 35	0.00 (0.00 - 0.10), 45	0.00 (0.00 - 0.09), 47
Kole	PfDHFR	A16V	0.00 (0.00 - 0.10), 46	0.00 (0.00 - 0.09), 47	0.00 (0.00 - 0.08), 59	0.00 (0.00 - 0.09), 50	0.00 (0.00 - 0.04), 109
		N51I	1.00 (0.91 - 1.00), 48	1.00 (0.91 - 1.00), 48	1.00 (0.93 - 1.00), 63	1.00 (0.92 - 1.00), 55	1.00 (0.96 - 1.00), 122
		C59R	0.96 (0.85 - 0.99), 48	0.94 (0.82 - 0.98), 48	0.97 (0.88 - 0.99), 63	0.98 (0.89 - 1.00), 55	0.98 (0.92 - 0.99), 122
		S108N	1.00 (0.90 - 1.00), 44	1.00 (0.91 - 1.00), 47	1.00 (0.92 - 1.00), 58	1.00 (0.91 - 1.00), 49	1.00 (0.96 - 1.00), 119
		I164L	0.20 (0.09 - 0.35), 41	0.08 (0.03 - 0.21), 48	0.10 (0.04 - 0.22), 58	0.02 (0.00 - 0.14), 42	0.22 (0.15 - 0.31), 109
	PfDHPS	I431V	0.00 (0.00 - 0.09), 48	0.00 (0.00 - 0.11), 40	0.00 (0.00 - 0.07), 60	0.00 (0.00 - 0.08), 55	0.00 (0.00 - 0.04), 109
		S436A	0.00 (0.00 - 0.09), 48	0.03 (0.00 - 0.15), 40	0.03 (0.01 - 0.13), 58	0.00 (0.00 - 0.08), 55	0.00 (0.00 - 0.04), 109
		A437G	1.00 (0.91 - 1.00), 48	0.98 (0.85 - 1.00), 40	0.97 (0.87 - 0.99), 60	1.00 (0.92 - 1.00), 55	1.00 (0.96 - 1.00), 109
		K540E	1.00 (0.90 - 1.00), 45	0.97 (0.85 - 1.00), 39	0.98 (0.90 - 1.00), 61	0.98 (0.90 - 1.00), 59	0.99 (0.95 - 1.00), 117
		A581G	0.04 (0.01 - 0.15), 51	0.02 (0.00 - 0.12), 49	0.02 (0.00 - 0.09), 65	0.02 (0.00 - 0.10), 62	0.03 (0.01 - 0.09), 120
		A613S	0.00 (0.00 - 0.09), 49	0.00 (0.00 - 0.09), 49	0.00 (0.00 - 0.07), 61	0.00 (0.00 - 0.07), 61	0.00 (0.00 - 0.04), 113
Lamwo	PfDHFR	A16V	0.00 (0.00 - 0.09), 51	0.00 (0.00 - 0.10), 42	0.00 (0.00 - 0.08), 58	0.00 (0.00 - 0.08), 55	0.00 (0.00 - 0.09), 49
		N51I	1.00 (0.92 - 1.00), 55	1.00 (0.90 - 1.00), 46	1.00 (0.93 - 1.00), 62	0.96 (0.87 - 0.99), 57	1.00 (0.92 - 1.00), 57
		C59R	0.91 (0.79 - 0.97), 55	1.00 (0.90 - 1.00), 46	0.94 (0.84 - 0.98), 62	0.91 (0.80 - 0.97), 57	0.95 (0.84 - 0.99), 57
		S108N	1.00 (0.92 - 1.00), 53	1.00 (0.90 - 1.00), 46	1.00 (0.93 - 1.00), 62	0.98 (0.89 - 1.00), 53	1.00 (0.92 - 1.00), 56
		I164L	0.15 (0.07 - 0.29), 52	0.04 (0.01 - 0.16), 47	0.05 (0.01 - 0.15), 59	0.09 (0.03 - 0.22), 46	0.18 (0.09 - 0.33), 49
	PfDHPS	I431V	0.00 (0.00 - 0.08), 57	0.00 (0.00 - 0.10), 44	0.00 (0.00 - 0.07), 60	0.00 (0.00 - 0.08), 57	0.00 (0.00 - 0.09), 50
		S436A	0.02 (0.00 - 0.12), 52	0.02 (0.00 - 0.14), 44	0.00 (0.00 - 0.08), 59	0.00 (0.00 - 0.09), 50	0.00 (0.00 - 0.09), 48
		A437G	0.91 (0.80 - 0.97), 57	0.98 (0.86 - 1.00), 44	0.98 (0.90 - 1.00), 60	0.88 (0.76 - 0.95), 57	0.96 (0.85 - 0.99), 50
		K540E	0.93 (0.82 - 0.98), 56	0.95 (0.83 - 0.99), 44	0.98 (0.90 - 1.00), 63	0.89 (0.78 - 0.96), 57	0.91 (0.80 - 0.97), 58
		A581G	0.07 (0.02 - 0.17), 61	0.04 (0.01 - 0.15), 48	0.05 (0.01 - 0.15), 61	0.03 (0.01 - 0.12), 61	0.00 (0.00 - 0.08), 55

		A613S	0.00 (0.00 - 0.08), 57	0.00 (0.00 - 0.10), 46	0.00 (0.00 - 0.08), 57	0.00 (0.00 - 0.07), 61	0.00 (0.00 - 0.09), 51
Mubende	PfDHFR	A16V	0.00 (0.00 - 0.08), 59	0.00 (0.00 - 0.08), 54	0.00 (0.00 - 0.15), 29	0.00 (0.00 - 0.24), 16	0.00 (0.00 - 0.05), 88
		N51I	1.00 (0.93 - 1.00), 61	1.00 (0.92 - 1.00), 54	1.00 (0.85 - 1.00), 29	1.00 (0.77 - 1.00), 17	1.00 (0.95 - 1.00), 94
		C59R	0.95 (0.85 - 0.99), 61	1.00 (0.92 - 1.00), 54	1.00 (0.85 - 1.00), 29	1.00 (0.77 - 1.00), 17	0.99 (0.93 - 1.00), 94
		S108N	1.00 (0.92 - 1.00), 58	1.00 (0.92 - 1.00), 55	1.00 (0.82 - 1.00), 22	1.00 (0.75 - 1.00), 15	1.00 (0.95 - 1.00), 88
		I164L	0.44 (0.31 - 0.58), 57	0.37 (0.25 - 0.51), 57	0.48 (0.27 - 0.69), 23	0.69 (0.39 - 0.90), 13	0.53 (0.42 - 0.64), 81
	PfDHPS	I431V	0.00 (0.00 - 0.07), 60	0.00 (0.00 - 0.09), 49	0.00 (0.00 - 0.16), 27	0.00 (0.00 - 0.22), 18	0.00 (0.00 - 0.05), 92
		S436A	0.02 (0.00 - 0.10), 60	0.00 (0.00 - 0.09), 49	0.00 (0.00 - 0.16), 27	0.00 (0.00 - 0.22), 18	0.00 (0.00 - 0.05), 91
		A437G	1.00 (0.93 - 1.00), 60	1.00 (0.91 - 1.00), 49	1.00 (0.84 - 1.00), 27	1.00 (0.78 - 1.00), 18	0.99 (0.93 - 1.00), 92
		K540E	1.00 (0.93 - 1.00), 62	1.00 (0.90 - 1.00), 46	1.00 (0.85 - 1.00), 29	1.00 (0.79 - 1.00), 19	0.99 (0.93 - 1.00), 95
		A581G	0.34 (0.23 - 0.47), 64	0.30 (0.19 - 0.44), 57	0.44 (0.27 - 0.62), 32	0.38 (0.19 - 0.61), 21	0.49 (0.39 - 0.59), 100
		A613S	0.00 (0.00 - 0.07), 63	0.00 (0.00 - 0.08), 56	0.00 (0.00 - 0.16), 27	0.00 (0.00 - 0.19), 21	0.00 (0.00 - 0.05), 94
Tororo	PfDHFR	A16V	0.00 (0.00 - 0.07), 67	0.00 (0.00 - 0.08), 54	0.00 (0.00 - 0.14), 30	0.00 (0.00 - 0.06), 71	0.00 (0.00 - 0.08), 54
		N51I	1.00 (0.93 - 1.00), 67	1.00 (0.92 - 1.00), 57	1.00 (0.86 - 1.00), 30	1.00 (0.94 - 1.00), 72	1.00 (0.93 - 1.00), 64
		C59R	0.96 (0.87 - 0.99), 67	0.98 (0.89 - 1.00), 57	1.00 (0.86 - 1.00), 30	0.97 (0.89 - 1.00), 72	1.00 (0.93 - 1.00), 64
		S108N	1.00 (0.93 - 1.00), 61	1.00 (0.92 - 1.00), 56	1.00 (0.86 - 1.00), 30	1.00 (0.93 - 1.00), 68	1.00 (0.91 - 1.00), 50
		I164L	0.07 (0.02 - 0.17), 59	0.11 (0.04 - 0.23), 56	0.17 (0.07 - 0.36), 29	0.09 (0.04 - 0.20), 64	0.17 (0.08 - 0.31), 47
	PfDHPS	I431V	0.00 (0.00 - 0.06), 71	0.00 (0.00 - 0.08), 55	0.00 (0.00 - 0.14), 31	0.00 (0.00 - 0.06), 74	0.00 (0.00 - 0.09), 52
		S436A	0.01 (0.00 - 0.09), 71	0.00 (0.00 - 0.09), 51	0.00 (0.00 - 0.14), 30	0.00 (0.00 - 0.06), 74	0.02 (0.00 - 0.12), 51
		A437G	1.00 (0.94 - 1.00), 71	1.00 (0.91 - 1.00), 51	1.00 (0.86 - 1.00), 30	1.00 (0.94 - 1.00), 74	1.00 (0.91 - 1.00), 51
		K540E	1.00 (0.93 - 1.00), 67	1.00 (0.91 - 1.00), 52	1.00 (0.86 - 1.00), 31	1.00 (0.94 - 1.00), 76	1.00 (0.93 - 1.00), 60
		A581G	0.06 (0.02 - 0.15), 70	0.03 (0.01 - 0.13), 58	0.03 (0.00 - 0.18), 33	0.01 (0.00 - 0.08), 77	0.06 (0.02 - 0.16), 64
		A613S	0.00 (0.00 - 0.06), 70	0.00 (0.00 - 0.08), 56	0.00 (0.00 - 0.13), 32	0.00 (0.00 - 0.06), 75	0.00 (0.00 - 0.08), 57

Supplementary Table 2. Spatial and temporal prevalence of PfDHFR I164L and PfDHPS A581G

Site	Genotype	Proportion Mutant (95% CI), N				
		2016-17	2018-19	2020	2021	2022
Agago	PfDHFR I164L	0.06 (0.02 - 0.18); 50	0.03 (0.00 - 0.15); 38	0.06 (0.02 - 0.17); 51	0.00 (0.00 - 0.10); 44	0.06 (0.02 - 0.16); 63
Agago	PfDHPS A581G	0.07 (0.02 - 0.17); 59	0.03 (0.00 - 0.15); 39	0.00 (0.00 - 0.08); 53	0.00 (0.00 - 0.07); 63	0.04 (0.01 - 0.13); 68
Amolatar	PfDHFR I164L	0.12 (0.05 - 0.25); 51	0.24 (0.08 - 0.50); 17	0.09 (0.03 - 0.22); 46	0.09 (0.02 - 0.24); 35	1.00 (0.60 - 1.00); 8
Amolatar	PfDHPS A581G	0.08 (0.03 - 0.19); 60	0.00 (0.00 - 0.27); 14	0.00 (0.00 - 0.09); 52	0.00 (0.00 - 0.08); 58	0.00 (0.00 - 0.25); 15
Arua	PfDHFR I164L	0.10 (0.04 - 0.21); 60	0.03 (0.00 - 0.17); 34	0.06 (0.02 - 0.18); 49	0.07 (0.02 - 0.17); 59	0.19 (0.11 - 0.31); 68
Arua	PfDHPS A581G	0.15 (0.08 - 0.27); 65	0.09 (0.02 - 0.25); 33	0.14 (0.06 - 0.27); 50	0.20 (0.12 - 0.32); 74	0.15 (0.08 - 0.25); 80
Hoima	PfDHFR I164L	-	0.21 (0.13 - 0.33); 70	0.21 (0.13 - 0.32); 80	0.09 (0.04 - 0.21); 53	0.20 (0.11 - 0.34); 50
Hoima	PfDHPS A581G	-	0.14 (0.08 - 0.25); 76	0.10 (0.05 - 0.19); 81	0.22 (0.14 - 0.32); 92	0.00 (0.00 - 0.07); 68
Jinja	PfDHFR I164L	0.28 (0.16 - 0.44); 43	0.26 (0.17 - 0.37); 82	0.56 (0.45 - 0.66); 90	0.01 (0.00 - 0.08); 80	0.83 (0.74 - 0.90); 101
Jinja	PfDHPS A581G	0.05 (0.01 - 0.15); 61	0.02 (0.00 - 0.09); 82	0.17 (0.08 - 0.31); 47	0.01 (0.00 - 0.07); 95	0.00 (0.00 - 0.05); 99
Kapchorwa	PfDHFR I164L	-	0.00 (0.00 - 0.27); 14	-	0.00 (0.00 - 0.12); 35	0.00 (0.00 - 0.19); 21
Kapchorwa	PfDHPS A581G	-	0.00 (0.00 - 0.28); 13	-	0.00 (0.00 - 0.05); 85	0.00 (0.00 - 0.15); 28
Kabale	PfDHFR I164L	0.22 (0.10 - 0.39); 37	0.33 (0.11 - 0.65); 12	0.36 (0.19 - 0.56); 28	0.61 (0.49 - 0.72); 72	0.76 (0.62 - 0.86); 54
Kabale	PfDHPS A581G	0.73 (0.57 - 0.85); 41	0.40 (0.14 - 0.73); 10	0.42 (0.26 - 0.61); 33	0.82 (0.72 - 0.89); 85	0.80 (0.68 - 0.89); 65
Kaabong	PfDHFR I164L	-	0.00 (0.00 - 0.11); 40	0.00 (0.00 - 0.11); 40	0.02 (0.00 - 0.13); 46	0.03 (0.01 - 0.11); 69
Kaabong	PfDHPS A581G	-	0.00 (0.00 - 0.11); 39	0.00 (0.00 - 0.10); 44	0.08 (0.03 - 0.18); 66	0.00 (0.00 - 0.06); 76
Koboko	PfDHFR I164L	-	0.00 (0.00 - 0.16); 27	0.00 (0.00 - 0.11); 38	0.00 (0.00 - 0.14); 30	0.00 (0.00 - 0.11); 41
Koboko	PfDHPS A581G	-	0.15 (0.05 - 0.36); 26	0.14 (0.06 - 0.29); 43	0.10 (0.04 - 0.23); 49	0.04 (0.01 - 0.14); 52
Kanungu	PfDHFR I164L	0.30 (0.20 - 0.43); 66	0.44 (0.31 - 0.58); 57	0.49 (0.37 - 0.60); 78	0.41 (0.19 - 0.67); 17	0.65 (0.51 - 0.78); 52
Kanungu	PfDHPS A581G	0.57 (0.45 - 0.67); 83	0.43 (0.31 - 0.57); 60	0.38 (0.28 - 0.50); 81	0.47 (0.34 - 0.60); 62	0.37 (0.26 - 0.49); 73
Kole	PfDHFR I164L	0.20 (0.09 - 0.35); 41	0.08 (0.03 - 0.21); 48	0.10 (0.04 - 0.22); 58	0.02 (0.00 - 0.14); 42	0.27 (0.18 - 0.38); 83
Kole	PfDHPS A581G	0.04 (0.01 - 0.15); 51	0.02 (0.00 - 0.12); 49	0.02 (0.00 - 0.09); 65	0.02 (0.00 - 0.10); 62	0.04 (0.01 - 0.11); 84
Kasese	PfDHFR I164L	-	0.49 (0.33 - 0.65); 39	0.49 (0.37 - 0.61); 71	0.52 (0.39 - 0.65); 60	0.31 (0.22 - 0.41); 91

Kasese	PfDHPS A581G	-	0.56 (0.40 - 0.71); 41	0.49 (0.38 - 0.61); 73	0.43 (0.32 - 0.54); 82	0.38 (0.29 - 0.49); 99
Katakwi	PfDHFR I164L	-	0.02 (0.00 - 0.10); 59	0.00 (0.00 - 0.09); 47	0.09 (0.03 - 0.22); 46	0.06 (0.02 - 0.16); 65
Katakwi	PfDHPS A581G	-	0.00 (0.00 - 0.07); 61	0.00 (0.00 - 0.09); 51	0.02 (0.00 - 0.10); 64	0.00 (0.00 - 0.06); 83
Lamwo	PfDHFR I164L	0.15 (0.07 - 0.29); 52	0.04 (0.01 - 0.16); 47	0.05 (0.01 - 0.15); 59	0.09 (0.03 - 0.22); 46	0.18 (0.09 - 0.33); 49
Lamwo	PfDHPS A581G	0.07 (0.02 - 0.17); 61	0.04 (0.01 - 0.15); 48	0.05 (0.01 - 0.15); 61	0.03 (0.01 - 0.12); 61	0.00 (0.00 - 0.08); 55
Mubende	PfDHFR I164L	0.44 (0.31 - 0.58); 57	0.37 (0.25 - 0.51); 57	0.48 (0.27 - 0.69); 23	0.69 (0.39 - 0.90); 13	0.54 (0.41 - 0.67); 57
Mubende	PfDHPS A581G	0.34 (0.23 - 0.47); 64	0.30 (0.19 - 0.44); 57	0.44 (0.27 - 0.62); 32	0.38 (0.19 - 0.61); 21	0.50 (0.38 - 0.62); 64
Tororo	PfDHFR I164L	0.07 (0.02 - 0.17); 59	0.11 (0.04 - 0.23); 56	0.17 (0.07 - 0.36); 29	0.09 (0.04 - 0.20); 64	0.17 (0.08 - 0.31); 47
Tororo	PfDHPS A581G	0.06 (0.02 - 0.15); 70	0.03 (0.01 - 0.13); 58	0.03 (0.00 - 0.18); 33	0.01 (0.00 - 0.08); 77	0.06 (0.02 - 0.16); 63
