# Supplementary material for: Multiple local PfDHFR I164L haplotype expansions drive Plasmodium falciparum antifolate resistance in Uganda
Source: Nat Commun. 2026 Sep 4;17:9552. doi: 10.1038/s41467-026-76826-4 (PMC13550557; doi:10.1038/s41467-026-76826-4)
Supplement: Supplementary file 2 [file 41467_2026_76826_MOESM2_ESM.pdf]

Description of Additional Supplementary Files

Supplementary Data 1. IBD clusters and their characteristics.
