# Supplementary material for: Multiple local PfDHFR I164L haplotype expansions drive Plasmodium falciparum antifolate resistance in Uganda
Source: Nat Commun. 2026 Sep 4;17:9552. doi: 10.1038/s41467-026-76826-4 (PMC13550557; doi:10.1038/s41467-026-76826-4)
Supplement: Supplementary file 3 [file 41467_2026_76826_MOESM4_ESM.pdf]

nature portfolio

Corresponding author(s): NCOMMS-25-72553A-Z

Last updated by author(s): Jul 22, 2026

Reporting Summary

Nature Portfolio wishes to improve the reproducibility of the work that we publish. This form provides structure for consistency and transparency in reporting. For further information on Nature Portfolio policies, see our Editorial Policies and the Editorial Policy Checklist.

Statistics

For all statistical analyses, confirm that the following items are present in the figure legend, table legend, main text, or Methods section.

n/a	Confirmed	
☐	☒	The exact sample size (*n*) for each experimental group/condition, given as a discrete number and unit of measurement
☐	☒	A statement on whether measurements were taken from distinct samples or whether the same sample was measured repeatedly
☐	☒	The statistical test(s) used AND whether they are one- or two-sided<br>*Only common tests should be described solely by name; describe more complex techniques in the Methods section.*
☐	☒	A description of all covariates tested
☐	☒	A description of any assumptions or corrections, such as tests of normality and adjustment for multiple comparisons
☐	☒	A full description of the statistical parameters including central tendency (e.g. means) or other basic estimates (e.g. regression coefficient) AND variation (e.g. standard deviation) or associated estimates of uncertainty (e.g. confidence intervals)
☐	☒	For null hypothesis testing, the test statistic (e.g. *F*, *t*, *r*) with confidence intervals, effect sizes, degrees of freedom and *P* value noted<br>*Give P values as exact values whenever suitable.*
☒	☐	For Bayesian analysis, information on the choice of priors and Markov chain Monte Carlo settings
☒	☐	For hierarchical and complex designs, identification of the appropriate level for tests and full reporting of outcomes
☒	☐	Estimates of effect sizes (e.g. Cohen's *d*, Pearson's *r*), indicating how they were calculated

Our web collection on statistics for biologists contains articles on many of the points above.

Software and code

Policy information about availability of computer code

Data collection	Software and tools used to collect genotype data.<br>• Analysis of sequence data was performed using MIPTools (https://github.com/bailey-lab/MIPTools), with variant calls for drug-resistant alleles performed with Freebayes software (v1.3.5).
Data analysis	Software and codes used to analyse the data reported<br>• Complexity of infection was calculated using the THEREALMcCOIL tool (https://github.com/EPPIcenter/THEREALMcCOI).<br>• Identity by Descent (IBD) was performed using Hidden Markov Model-based IBD detection algorithm implemented in hmmIBD-rs<br>• Bayesian regression model was fitted using probabilistic programming language Stan in brms R package<br>• Scans for signals of positive selection was performed using the R package rehh (v3.2.2).<br>• Codes used for data wrangling, generations of summary statistics and figures, and statistical tests are available, together with data sets in the GitHub repository: https://github.com/victorasua/Antifolate_Analysis/tree/main/AF-Evol-20260401

For manuscripts utilizing custom algorithms or software that are central to the research but not yet described in published literature, software must be made available to editors and reviewers. We strongly encourage code deposition in a community repository (e.g. GitHub). See the Nature Portfolio guidelines for submitting code & software for further information.

Data

Policy information about availability of data

All manuscripts must include a data availability statement. This statement should provide the following information, where applicable:

- Accession codes, unique identifiers, or web links for publicly available datasets
- A description of any restrictions on data availability
- For clinical datasets or third party data, please ensure that the statement adheres to our policy

Provide your data availability statement here.

Research involving human participants, their data, or biological material

Policy information about studies with human participants or human data. See also policy information about sex, gender (identity/presentation), and sexual orientation and race, ethnicity and racism.

Reporting on sex and gender	Sex and gender were not included in our analysis, therefore not reported
Reporting on race, ethnicity, or other socially relevant groupings	No data collected on race, ethnicity or other socially relevant groupings.
Population characteristics	No human subject specific characteristics were not included. Our manuscript focused on the parasite isolates. However, for samples collected in 2016-2017, only children under 10 years were included. But this age restriction was eliminated in the collections of later years, though not reported.
Recruitment	Participants were recruited between June-August of each year, except the second collection which was done from December - January. During each sampling period, two to five participants were randomly sampled per day until the target sample size for that facility was reached
Ethics oversight	Ethical oversight was sort from Makerere University School of Biomedical Sciences Research Ethics Committee

Note that full information on the approval of the study protocol must also be provided in the manuscript.

Field-specific reporting

Please select the one below that is the best fit for your research. If you are not sure, read the appropriate sections before making your selection.

☒ Life sciences ☐ Behavioural & social sciences ☐ Ecological, evolutionary & environmental sciences

For a reference copy of the document with all sections, see nature.com/documents/nr-reporting-summary-flat.pdf

Life sciences study design

All studies must disclose on these points even when the disclosure is negative.

Sample size	Sample size calculations were based on the need to sample multiple sites. Samples were collected from MRCs, representing all regions and malaria endemicities of Uganda. Our primary analysis compared the prevalence of P. falciparum genetic polymorphisms between sites and over time in Uganda. Using a two-sided t-test to identify a 30% difference in prevalence, we required 38 participants at each site to afford 80% power with a confidence level of 95%. Considering 20% loss of data due to failed assays, up to 100 samples were collected from each site every 6-12 months
Data exclusions	Attempt was made to sequence every sample. However, those that failed sequencing were excluded.
Replication	Our study was population-based spatial-temporal genomic surveillance. Therefore, experimental replication was not possible.
Randomization	The study had no interventions, therefore randomisation was not required
Blinding	This was not application to our study design

Reporting for specific materials, systems and methods

We require information from authors about some types of materials, experimental systems and methods used in many studies. Here, indicate whether each material, system or method listed is relevant to your study. If you are not sure if a list item applies to your research, read the appropriate section before selecting a response.

Materials & experimental systems

n/a	Involved in the study
☒	☐ Antibodies
☒	☐ Eukaryotic cell lines
☒	☐ Palaeontology and archaeology
☒	☐ Animals and other organisms
☒	☐ Clinical data
☒	☐ Dual use research of concern
☒	☐ Plants

Methods

n/a	Involved in the study
☒	☐ ChIP-seq
☒	☐ Flow cytometry
☒	☐ MRI-based neuroimaging

Plants

Seed stocks

Not applicable

Novel plant genotypes

Not applicable

Authentication

Not applicable
