# Supplementary material for: Multiple local PfDHFR I164L haplotype expansions drive Plasmodium falciparum antifolate resistance in Uganda
Source: Nat Commun. 2026 Sep 4;17:9552. doi: 10.1038/s41467-026-76826-4 (PMC13550557; doi:10.1038/s41467-026-76826-4)
Supplement: Supplementary file 4 [file 41467_2026_76826_MOESM5_ESM.pdf]

nature portfolio

Peer Review File

Multiple local PfDHFR I164L haplotype expansions drive Plasmodium falciparum antifolate resistance in Uganda

Corresponding Author: Mr Victor Asua

This file contains all reviewer reports in order by version, followed by all author rebuttals in order by version.

Version 0:

Reviewer comments:

Reviewer #1

(Remarks to the Author)
Asua et al. use genomics to explore the spatiotemporal landscape of P. falciparum antifolate resistance across Uganda over an 8yr period. They focus on changes in PfDHFR 164L prevalence, which confers high-level pyrimethamine resistance. The emergence and changing prevalence of PfDHFR 164L in Uganda has been reported by the same authors in several previous publications (e.g. Tumwebaze et al. [2015] AAC; Tumwebaze et al. [2017] JID; Asua et al. [2019] AAC; Asua et al. [2021] JID); and the dataset used here has been published previously (I believe, the authors should clarify), with a focus on kelch13 markers (Conrad et al. [2023] NEJM). Here, the primary novelty is the presentation of additional genomic analyzes, performed to gain a deeper insight into signals of parasite relatedness and selection that may explain prevalence changes in PfDHFR 164L. Unfortunately, I found the main claims of the paper – i.e. a rapid clonal expansion of I164L – are not clearly and convincingly supported by the data; and there seem to be errors in the figures / text which have made reviewing the manuscript difficult.

Major Comments
1. A main claim of the manuscript is that PfDHFR I164L has increased in prevalence across Uganda. This is highlighted in the Abstract, lines 41-42: "PfDHFR I164L increased from 19% in 2016-17 to 34% in 2022". However, these years used different sample collection sites (10 vs 16, Methods, line 323) and so the trend is confounded by possible spatial heterogeneity. Was this controlled for in some way? If so, please describe this in the manuscript. If not, this should be controlled as, looking at the presented data, there does seem to be substantial spatial heterogeneity which could impact the magnitude / conclusion.
2. In addition to claiming that PfDHFR 164L is expanding in Uganda, the authors claim that this expansion is rapid (e.g. in the title). But the data do not seem to support this and, moreover, there are errors in data reporting that complicate interpretation. For example, in the Discussion lines 236-40: "The prevalence of PfDHFR 164L increased from 2016-17 to 2022 (Fig. 3), with particularly pronounced increases (more than threefold) at four sites outside southwestern Uganda (Arua, Amolatar, Jinja, and Lamwo; Supplementary Table 2)." First, Lamwo does not have increasing 164L in Fig 3 or in STable 1 (note STable 2 is also the wrong reference); this seems to be an error. Second, in STable 1, Aura's prevalence change was 10% (2016) to 20% (2022), which is only a two-fold change, and moreover the confidence intervals overlap. Third, for Amolatar, the small sample size in 2022 (8 samples) means the results should be interpreted with caution, as they could be extremely sensitive to sampling bias. In addition, in Results, line 116: "Amolator 12% (6/50) to 100% (8/8)"; but in Fig. 3, this is not the case: the site is missing in 2022, and in 2021 the prevalence was <20%. For Jinja, the prevalence of PfDHFR 164L seems to fluctuate dramatically, e.g. 56% (2020), 1% (2021), 84% (2022) – there are major decreases and increases; not a consistent rapid increase. Moreover, later in the Discussion, lines 251-253, the authors state: "PfDHFR 164L and PfDHPS 581G have been at high prevalence in southwestern Uganda since the late 1990s35–37, and now show relatively stable prevalence…" Yet two sites with arguably the most steady and convincing increases in PfDHFR 164L are both in SW Uganda (Kabale and Kanungu). Given these points, I am not convinced that PfDHFR 164L is rapidly expanding outside of SW Uganda.
3. To help address 2., the longitudinal data of PfDHFR I164L across Uganda needs to be much more clearly presented, given its centrality to the paper. Presently data is either in the map in Fig. 3 (which is quite hard to read, uses arbitrary prevalence bins, and doesn't show CIs) or buried in STable 1. To fix this, a plot of longitudinal trends of PfDHFR I164L for different sites and overall should be included in Fig. 3. It would also help to have supplementary tables specifically looking at PfDHFR I164L across years and sites (e.g. columns years, rows sites, values prevalence), since it is the main focus of the paper.

4. In trying to look into comment 1. further, I found several inconsistencies / ambiguities in the presentations of the 164L maps. From Methods, line 323, there should be 10 collection sites in 2016-17, and 16 thereafter. Yet for I164L, Figure 3 displays 14 sites, which are shown across all years (except 2022, which has 12). Perhaps 'Not detected' means 'No data collected this year'? But if so: (i) why is there no bin for zero prevalence and (ii) why do sites disappear from 2021 to 2022? Maybe the Methods are wrong? Fig. 1 does show 16 sampling sites, but the administrative boundaries seem to differ from Fig. 3. This makes it hard to follow some of the Results text, which highlight specific sites (requiring finding the sites in Fig. 1, and then matching them to Fig. 3). These issues should be resolved, and more information should be included in figure legends to add clarity.

5. In Results line 112, the claim is also made that the PfDHFR I164L mutation spread across sites from 2016-17 to 2022. This is important and interesting if true. However, looking at Fig. 3 the number of sites with >20% PfDHFR I164L varies, with no clear evidence of spread as far as I can tell. Similar to comment 2., the argument would benefit from numerical analysis supporting that PfDHFR I164L is spreading across Uganda (as distinct from increasing at specific sites).

6. I am not sure that the analysis in Results 'Clonal transmission of the PfDHFR 164L allele' and in Fig. 4 provides evidence of clonal transmission. Given that the 164L mutation seems more geographically restricted than wild-type allele, simple isolation by distance (e.g. more distant populations are more genetically diverse) would be sufficient to explain these observations. For example, the average distance between two random wild-type pairs will be farther than 164L pairs (I expect). The fact that most of the signal comes from Jinja seems to support this. To disentangle the effects of spatial distribution and clonal transmission, you would need to sample an equal number of wild-type pairs from the same sites as were sampled for PfDHFR 164L.

7. I am having some issues linking data in Fig. 5 with results. In particular, lines 154-156 state 100% of 164L pairs in Hoima have >0.5 IBD, but I see no 164L pairs in the figure. Similarly, I think there should be pairs for Jinja in 2020, and for Kabale, but none are visible. I am not sure if this is due to overplotting or something else, but it makes it hard for readers to independently assess the data. This data should be visualized more clearly, perhaps by binning IBD intervals and showing proportion of pairs falling within a given IBD bin.

8. In the results section 'Differential patterns of PfDHFR 164L …' The high levels of pairwise IBD between I164L samples is used to suggest possible clonal expansion of I164L, with the implication being (in my reading) that it is under selection (e.g. lines 157-159, 168-169). But in general, genome-wide relatedness signals like these are more commonly indicators of demographic changes or structure, rather than selection, which acts on a specific locus. Moreover with P.f. malaria, low transmission alone can lead to clonal propagation of parasites due to a paucity of polyclonal infections, which are required for outcrossing in the mosquito; all in the absence of any sort of selection. Given that (i) several wild-type clusters with high-relatedness were observed, often in the same sites where 164L had high relatedness; and (ii) over half of sites had a decline in COI over the study period (suggesting low / reducing transmission) -- is this not much more likely than selection? On lines 267 the authors state that wild-type clonal propagation is 'paradoxical', but it is completely expected (and has been observed before, e.g. in Senegal), in cases where transmission is low or populations have decreased in size.

Minor Comments

• Are the samples and sequencing data used here the same as in Conrad et al. (2023) NEJM? If so, this needs to be clearly stated in the 'Study samples' section of the methods.
• Abstract, lines 43-44: "haplotype structure suggested multiple origins of PfDHFR I164L" and then Results, lines 186-187, in reference to 164L: "This pattern suggests a single origin several generations ago, …". These two interpretations of the data seem to contradict.
• Abstract 43-44 : Why 'Although', aren't these findings consistent with each other?
• I am struggling to understand how the genotypes were called. In the Methods lines 341-342, it says mixed genotypes were called if WSAF > 0.05. But in Fig. 2b, it looks by eye that there are mixed genotypes called with <0.05 (some points, esp. for 581G, are very close to 0.0 or 1.0), which would be inconsistent with the methods. Moreover, in Fig. 2b why are there 'mutant' genotypes with such low WSAF (e.g. < 0.9); should these not be mixed? How were homozygous mutant genotypes called? Are you naively using the genotype calls from FreeBayes, and then using WSAF as a post hoc filter? Or are you calling variant sites with FreeBayes, and then genotypes using the WSAF and depth? Overall, it is important the genotyping procedure is described in more detail in the Methods and Fig. 2 figure legend, as it impacts all downstream analyses.
• Fig. 2 is just sequencing QC and should not be a main results figure; especially given there are already 8 main figures. Moreover, previous work from overlapping authors has shown that WSAF from MIPs are quite unreliable, I am not sure why it is being presented in a main figure (https://www.researchsquare.com/article/rs-5743980/v1).
• Note that lines 103-104 needs rewording, as homozygous reference is also a genotype.
• The COI tool used (TheRealMcoil) assumes no within-host IBD, yet it seems a lot of these samples have IBD. The authors should remark on this in the manuscript, as the COI estimates may be biased downwards.
• In Fig. 5, why does the proportion of IBD seem to hit a maximum at 0.8? What is being used to define the denominator of these proportions? This is confusing because presumably the same data is used to create the IBD network in Fig. 7 which requires IBD > 0.9.
• For many of the IBD analyses, samples are divided into I164L, 581G, and WT; but these categories are not mutually exclusive. Were there 164L and 581G double mutants? How many, and how were they handled for the IBD and haplotype analyses?
• In Fig. 7, for many clusters (e.g. Jinja 2016) it is very hard to see the genotype because the points overlap. Maybe either it is better to use only color, and have three networks, one displaying genotypes? Or can you replot so the points don't overlap?
• Line 182: the section is called 'Expansion of multiple haplotypes of PfDHFR 164L', but no evidence is presented that multiple haplotypes have expanded. Evidence of 164L increase in prevalence was presented previously, but was this driven by frequency increase of specific haplotypes identified here? If so, can you present that data? If not, I would suggest renaming 'Multiple distinct haplotypes of PfDHFR 164L' or similar.
• In Fig. 8 and SFig. 3, can I confirm that the REF and ALT alleles are labelled correctly? As currently labelled, there are a

large number of fixed ALT sites, which is atypical.
• Fig 8a: Please provide some labelling of the x-axis to give readers a sense of scale of the conserved region.
• Lines 128-130: Do you have malaria prevalence or incidence data to support the claim that COI decline is evidence of reduced transmission in these sites? In theory COI correlates with transmission, but often this correlation is weak and confounded by other phenomenon (e.g. migration), and so can't be interpreted in this way so easily.
• Lines 184-188: The authors claim that a shared core haplotype flanked by diversity is indicative of 'a single origin several generations ago, followed by recombination events that broke down the shared haplotype over time'. Can they clarify how big the core haplotype is in the text or figure? This claim can only be made if there was initially sufficient diversity present across this core haplotype. Otherwise, it is also possible that 164L emerged multiple times on the same core haplotype background. Given that PfDHFR has been under selection for a long time and resistance haplotypes have spread across Africa, this wouldn't be too surprising, no?
• Line 278: 'sulfamethoxazole-trimethoprim' and then line 295 'Trimethoprim-Sulfamethoxazole', please use consistent nomenclature.
• For the authors awareness, in the PDF version of the manuscript the figures were very low resolution, and the word document was quite large (21Mb) and sometimes laggy.

Reviewer #2

(Remarks to the Author)
Summary
Overall, this is an interesting and novel research paper, looking into variants in pfdhfr and pfdhps in >4000 field isolates from 16 sites in Uganda taken during the period of 2016-2022. Through genotyping of 11 SNP positions in pfdhfr/pfdhps, flanking regions surrounding the genes and SNPs from a genome wide panel, the manuscript investigates the prevalence of variants over time in addition to the selective pressures on these variants.
Both pfdhfr 164L and pfdhps 581G have been present in Uganda for a while, with some clonal transmission of pfdhfr 164L in distinct foci and the possibility of this variant arising from multiple origins, and a less clear picture for pfdhps 581G. The manuscript specifically shows clonal expansion of pfdhfr 164L geographically restricted to the area of Jinja, with signals of recent positive selection, which is important when considering malaria interventions in this region.

Overall comments:

1. Whilst documenting the presence of these variants in Uganda is not novel, there has not been a previous study looking into selection dynamics of these alleles at these sites, and this study uses time-series data and a large dataset to enable this investigation. It would be interesting to compare this data to other global sequence data of these genes to determine where variant alleles have likely been spread from.
Previous pfdhfr mutations have been shown to spread to Africa from Southeast Asia (https://www.science.org/doi/10.1126/science.1098876). Could the authors compare sequence data from a pool of Southeast Asian isolates around this locus to also determine geographical spread? Or if this already has been shown, could the authors please refer to this in their manuscript?

2. The sequence data used was generated using MIPs. The MIPs targeted drug resistance genes, regions flanking dhfr/dhps and "single-nucleotide polymorphisms (SNPs) distributed throughout the P. falciparum genome"
Please can you expand on what these SNPs are? How many additional SNPs are covered? What regions are targeted to sequence these? Why are these chosen? Is it a previously published SNP panel?
I am also struggling to see the importance of these SNPs in the manuscript, and analysis of them specifically, were they used in popgen analysis? Where?

3. There are instances in the text when italics are not used for genus-species names. Please can you correct this? P. falciparum, not P. falciparum.

4. Sulfadoxine resistance, due to mutations in pfdhps, is known to have emerged multiple different times in Africa (https://pmc.ncbi.nlm.nih.gov/articles/PMC2661256/). Which is different to the triple dhfr mutant in Africa leading to pyrimethamine resistance (https://www.science.org/doi/10.1126/science.1098876) which originated in Southeast Asia and spread to Africa.
Your results have shown that isolates with the pfdhfr 146L mutation share more relatedness than isolates with the pfdhps 581G mutation, indicative of a clonal expansion of the 164L mutation in some regions, and multiple origins of the 581G mutation.
Please can you comment on this to contextualise your results in comparison to previous work looking at the spread of pfdhfr/pfdhps mutations? Do you think that these variants show a similar pattern to the spread of other resistant alleles in these genes?

Results:
Figure 1:
- It is difficult to read some district names in the map. I would suggest changing the district name to a darker colour, and for small districts such as Kabale or Jinja, it may be useful to annotate the name and number outside of the map, and to label with an arrow to the district?

- It could also be useful to shade the colour of the filled in districts on the map in a gradient based on how many isolates were obtained (this is similar though across most districts so may not change much).

Figure 2:
- The labelling is mixed up on the figure and the legend. Panel a shows the proportion of alternative alleles and the total number sequenced, panel b shows the WSAF at 146L and 581G. You should rewrite the figure legend.

- "The proportion of samples genotypes at each locus" is also incorrect – I think it is the number (not proportion) of samples genotyped with the proportion of variants in that set. Please correct if this is incorrect.

- "Genotypes were assigned to samples carrying alleles with a within-sample allele frequency (WSAF) > 0.05%" does not align with "Mutant alleles with WSAF < 1.0 and supported by <5 reads were excluded." - What was the WSAF cut off for identifying a genotype? WSAF =1? Or WSAF > 0.05? If the WSAF cut off was 1, you will be missing all alternative alleles in multiclonal samples. Can you explain why this was used as your cut off?

Figure 3:
- For each district, at each time point, it would be good to know what number of isolates there is data for whilst interpreting this map. - For example, many districts in 2016-2017 are grey due to 'not detected' – if there are 50 samples here instead of 200 samples in the year later, this could just be due to sampling size.
Perhaps only sites with > X amount of data are included in the map, but this needs to be stated in the figure legend if so.
- Data for Kabale is not included in the map for 581G in years 2018-19 and 2022 – why is this?

COI analysis:
"These spatial and temporal trends in poly-genomic infections reflect fluctuating transmission, with evidence of reduced malaria transmission in later years in some sites."
Is this published evidence? If so can you site it, or state if this is observations from individuals at the site in health clinics for example?

Figure 5/6:
In Figure 5, the colouring system for variants is: 164L – red, 581G – yellow, Wild-type – blue. Yet in Figure 6, the colouring system changes to 164L – yellow, 581G – grey, Wild-type – blue. Please can you keep a uniform colouring system where possible?

Figure 7:
Due to the clustering of the dhfr 164L isolates, it is difficult to decipher the symbol where there are lots of isolates. Could the symbols of this plot be changed to look more distinctive between genotypes? Or could the network maps be larger, with one below the other, to allow for the points in the network to be more spaced apart?

Methods:
For IBD analysis, a heavily filtered dataset is used, please state the number of samples remaining in this filtered dataset that are included in IBD analysis.
Additionally, a recent preprint compared IBD softwares on P. falciparum data demonstrated that hmmIBD has a lower false positive rate than isoRelate (https://elifesciences.org/reviewed-preprints/101924).
Could you also run hmmIBD on this dataset to determine if the same patterns are seen with IBD inferred from this software?

Reviewer #3

(Remarks to the Author)
Asua et al. present a large-scale genomic surveillance analysis of Plasmodium falciparum antifolate resistance in Uganda from 2016–2022, focusing on the dynamics of pfdhfr 164L and pfdhps 581G on a near-fixed quintuple-mutant background. Using targeted MIP-based deep sequencing, the authors generate 4,725 field isolates and analyse antifolate-resistance haplotypes with identity-by-descent (IBD), haplotype networks, and extended haplotype homozygosity (EHH). The manuscript is clearly written, well structured, and supported by detailed visualizations.

The dataset is substantial, geographically and temporally rich, and the laboratory and sequencing components are technically sound. Integrating IBD, haplotype structure, and temporal allele-frequency trajectories provides valuable insight into antifolate-resistance evolution. The study documents regional persistence of pfdhps 581G and increasing prevalence of pfdhfr 164L, and identifies clusters of highly related 164L parasites.

However, several aspects of the interpretation require clearer explanation and additional analysis. The distinction between selection and demographic effects needs further clarification, especially regarding claims of clonal spread that are not fully supported by the IBD values shown. The absence of WT-mutant relatedness comparisons, the consequences of dominant-clone filtering in the context of high polygenicity, and the limited integration of COI and regional transmission patterns leave key conclusions only partially supported. In addition, prevalence trends are tested repeatedly without addressing multiplicity, and some policy statements extend beyond the evidence. These issues require clarification before firm conclusions can be drawn.

Major comments

1) The authors repeatedly interpret the elevated relatedness among pfdhfr 164L parasites as evidence of "clonal spread".

The underlying relatedness signal is indeed strong; pfdhfr 164L pairs show substantially higher IBD than wild-type pairs, and this is clearly visible in the relatedness distributions presented in the Results. The authors use IBD ≥0.9 as the operational definition of clonality. However, IBD ≥0.9 indicates very close relatedness but is not, on its own, proof of single-clone transmission across space and time. Can the authors comment on this?
Figure 5 shows no pair with IBD = 1 (or very close to 1). Moreover, the proportion IBD data provided in the linked data file (Prop_IBD.csv; https://github.com/victorasua/Antifolate_Analysis/blob/main/AF_Evol/data/Prop_IBD.csv) indicate that the maximum IBD is 0.855419282552218, meaning that no sample pair in the dataset reaches the ≥0.9 threshold. So, I am a bit puzzled as to how Figures 4a/b and 7 were generated if this should show only pairs with IBD ≥0.9? If the dataset contains no pairs in this range, the logic behind the network shown in Figure 7 requires clarification, specifically the implicit claim of clonal transmission across space and time. As the authors mention, selection scans limited evidence of recent selection, maybe except for Jinja.

2) Adding to the above point, Figure 5 shows IBD sharing only for 164L pairs, 581G pairs and for wild-type pairs, but it does not display the IBD sharing for wild-type-mutant pairs. Why was this comparison omitted? Without the WT-mutant IBD sharing, it is difficult to determine whether the elevated relatedness reported among 164L parasites reflects locus-specific structuring driven by selection or instead mirrors the broader demographic background of the parasite population. Supplementary Table 3 shows that several regions with high 164L–164L IBD ≥0.5 also exhibit high WT-WT relatedness, consistent with demographic effects such as lower transmission, reduced recombination, or low COI. WT–mutant relatedness should be reported explicitly, either in Figure 5 or a separate figure/table.

3) IBD and haplotype analyses are restricted to samples meeting the “dominant clone” threshold (WSAF ≥0.9 across 95% of loci), yet polygenomic infections comprise a substantial proportion of samples at many sites, meaning that a large share of the dataset is potentially excluded from population-genomic analysis. This approach risks biasing relatedness and haplotype-diversity estimates toward patterns typical of low-transmission, low-COI settings. Figure 2 shows that a proportion of pfdhfr 164L samples have WSAF values below 0.9, implying that some 164L infections are polyclonal and therefore omitted from downstream analyses, potentially rendering the analysed subset unrepresentative of the full 164L-carrying population. The authors should report COI distributions by genotype (164L vs wild type), specify how many infections are strictly monoclonal (COI = 1), and assess whether elevated relatedness persists when analyses are limited to monoclonal infections. High-relatedness regions appear to overlap with low-polyclonality regions (Supplementary Figs. 1–2), reinforcing the need for this assessment. Providing transmission-intensity context for each region would further strengthen interpretation.

4) Prevalence changes are tested separately for sites and years without accounting for multiple comparisons. Several Results paragraphs refer to “significant increases” but do they also hold under correctionThe manuscript also does not specify whether comparisons were only between 2016 and 2022 or across all years; in some regions, 164L prevalence decreases mid-series (especially 2021). Given the high proportion of polygenomic infections, the authors may also need to consider COI as a covariate when interpreting prevalence changes.

Minor comments

Line 4: Affiliations are not listed in increasing numerical order.

Line 42: I would suggest replacing “IBD revealed clonal transmission” with something like “IBD analysis identified closely related parasite clusters.” To avoid over-interpretation (clonal would be an IBD of 1).

Line 43: The Abstract states that the authors find “limited evidence of recent selection,” while the Results section later claims “evidence of recent positive selection” in Jinja based on slower EHH decay around 164L compared to wild type. At the same time, the Results paragraphs describing COI and IBD patterns emphasize that high IBD is widespread among wild-type parasites due to declining transmission, which can mimic signatures of selection. Maybe this can be re-written into a single coherent interpretation or otherwise more clearly separated?

Line 77: Missing “and” after “Rwanda,”.

Line 104: Clarify whether the WSAF threshold is 0.05% or 0.05 (5%); it is inconsistent with Methods line 346.

Line 127: I think it would be interesting to provide some prevalence estimates across the years and regions if available to see if the decline in COI could be, at least in part, due to overall decline in prevalence? Do you have some info on MOI estimates, rather than just binary info on polyclonality?

Line 129: Clarify whether prevalence data are available.

Line 132: Italicize P. falciparum.

Line 137: Consider reporting overall relatedness by region.

Line 138: The origin of the number 94,637 pairs is unclear; 3,168 samples would yield (3,168*3,167)/2 = 5,016,528 pairwise comparisons. Please clarify which samples were analysed and provide some more details on those analyses.

Line 186: Here you suggest a single origin of the 164L haplotype, but in the Abstract and other sections you claim multiple origins of the 164L haplotype? So, you suggest its multiple independent emergences or a single emergence that then spread?

Line 191 / Figure 8b: Text describes 72 cluster members; figure shows 73. Harmonize.

Line 237: Any explanation as to why there seems to be a drop 164L prevalence in the year 2021 across multiple sites?

Line 239: The reference appears to be Supplementary Table 1.

Line 271 / 295: Use consistent terminology for trimethoprim–sulfamethoxazole.

Line 326: Some additional info on the sampling strategy would be good. Why those two months? How were the ~50 – 100 participants selected? Is this a random subset or did sampling continue until this number was reached? How representative are those samples?

Line 342: How were polyclonal infections handled for prevalence of resistance-associated alleles? Mixed would count as WT and ALT if WSAF >0.05?

Line 349: tate whether COI estimation used the categorical method; provide number of SNPs and number of samples retained.

Line 353: “a p-value > 0.05 considered as a significant monotonic change in prevalence”. I think this should be < 0.05.

Line 355: Again, provide some more details on how many SNPs and samples were retained after filtering. Why was IBD ≥0.9 chosen for network analysis (or different, why was IBD ≥ 0.9 considered clonal and not “near clonal”)?

Line 357: Verify whether allele-frequency threshold “≥0.01%” is correct.

Line 372: Clarify inconsistency between 200 kb and 50 kb flanking regions. Above you mention 200 kb regions flanking pfdhfr/pfdhps and here 50kb flanking. So, this means ~50 kb up/down stream for each gene?

Line 565 / Figure 1: Could add the specific sites to the map.

Line 605: Figure 2a/2b descriptions appear swapped; include sample counts for Fig. 2b.

Line 635: Provide sample counts for all panels or state them in the text.

Line 652 / Figure 7: I would be curious to see this plot also at lower IBD thresholds? If the spread was purely regional, then I would also expect clustering by region/year at lower thresholds. Do the authors have any demographic data (e.g. household) of the study participants? This could also be an important factor to consider when looking at relatedness, as individuals from the same/nearby households might be more likely to be infected with the same/similar clones.

Line 656: “First siblings”? Suggest using “full siblings” instead?

Line 677: Why are there nodes that are not connected to any other sample? I the legend you state, that the IBD network shows clonal pairs (IBD ≥ 0.9), which should mean that each node is connected to at least one other node, no (otherwise it’s not a pair)?

Line 715: Define iR, which is not introduced earlier.

Version 1:

Reviewer comments:

Reviewer #2

(Remarks to the Author)
Reviewer #2 (Remarks to the Author):

Summary
Overall, this is an interesting and novel research paper, looking into variants in pfdhfr and pfdhps in >4000 field isolates from 16 sites in Uganda taken during the period of 2016-2022. Through genotyping of 11 SNP positions in pfdhfr/pfdhps, flanking regions surrounding the genes and SNPs from a genome wide panel, the manuscript investigates the prevalence of variants over time in addition to the selective pressures on these variants. Both PfDHFR I164L and pfdhps 581G have been present in Uganda for a while, with some clonal transmission of PfDHFR I164L in distinct foci and the possibility of this variant

arising from multiple origins, and a less clear picture for pfdhps 581G. The manuscript specifically shows clonal expansion of PfDHFR I164L geographically restricted to the area of Jinja, with signals of recent positive selection, which is important when considering malaria interventions in this region.

Overall comments:

1. Whilst documenting the presence of these variants in Uganda is not novel, there has not been a previous study looking into selection dynamics of these alleles at these sites, and this study uses time-series data and a large dataset to enable this investigation. It would be interesting to compare this data to other global sequence data of these genes to determine where variant alleles have likely been spread from.
Previous pfdhfr mutations have been shown to spread to Africa from Southeast Asia (https://www.science.org/doi/10.1126/science.1098876). Could the authors compare sequence data from a pool of Southeast Asian isolates around this locus to also determine geographical spread? Or if this already has been shown, could the authors please refer to this in their manuscript?
Response: We thank the reviewer for this valuable suggestion. However, in our haplotype analysis, we identified multiple haplotypes surrounding the PfDHFR I164L variant, with no single haplotype being clearly dominant. Comparing these haplotypes to global sequences to trace their origin would be a complex undertaking, without a clear hypothesis or question to address. For the above reason, we respectively, reframed from performing global analysis.
Reviewer response: Thanks, agreed with the author that comparing the 17 I164L haplotypes described here to Southeast Asian sequence data would be a complex undertaking. However I do think it would be worthwhile to describe these results in the context of where I164L has been seen before e.g. a publication from 2010 (Origins of the Recent Emergence of Plasmodium falciparum Pyrimethamine Resistance Alleles in Madagascar, Andriantsoanirina, V. et al. 2010) has previously described that I164L is likely to have occurred on multiple occasions – has this been shown in southeast asia too? Perhaps this could be expanded in the discussion. I think the discussion hints that in southeast Asia there is one clonal expansion of the I164L allele due to hard selective sweeps, can you directly state if we also see I164L on multiple different genomic backgrounds in southeast asia too, or is there something specific also about this variant that increases the chances of it arising on multiple different genetic backgrounds.

The sequence data used was generated using MIPs. The MIPs targeted drug resistance genes, regions flanking dhfr/dhps and “single-nucleotide polymorphisms (SNPs) distributed throughout the P. falciparum genome” Please can you expand on what these SNPs are? How many additional SNPs are covered? What regions are targeted to sequence these? Why are these chosen? Is it a previously published SNP panel? I am also struggling to see the importance of these SNPs in the manuscript, and analysis of them specifically, were they used in popgen analysis? Where?
Response: We thank the reviewer for raising these important questions. Detailed information on the MIP targets, including their importance, is now provided in the methods section, lines 316-331, including reference to key publications.
Reviewer response: Thank you, this comment has been addressed.

2. There are instances in the text when italics are not used for genus-species names. Please can you correct this? P. falciparum, not P. falciparum.
Response: We apologise for the inconsistencies in P. falciparum formatting. These have now been corrected throughout the manuscript.
Reviewer response: Thank you, this comment has been addressed.

3. Sulfadoxine resistance, due to mutations in pfdhps, is known to have emerged multiple different times in Africa (https://pmc.ncbi.nlm.nih.gov/articles/PMC2661256/). Which is different to the triple dhfr mutant in Africa leading to pyrimethamine resistance (https://www.science.org/doi/10.1126/science.1098876) which originated in Southeast Asia and spread to Africa.
Your results have shown that isolates with the pfdhfr 146L mutation share more relatedness than isolates with the pfdhps 581G mutation, indicative of a clonal expansion of the 164L mutation in some regions, and multiple origins of the 581G mutation.
Please can you comment on this to contextualise your results in comparison to previous work looking at the spread of pfdhfr/pfdhps mutations? Do you think that these variants show a similar pattern to the spread of other resistant alleles in these genes?
Response: We thank the reviewer for this insightful comment. Indeed, previous studies have shown distinct evolutionary histories of antifolate resistance, with sulfadoxine resistance associated with pfdhps mutations arising multiple times independently in Africa, whereas pyrimethamine resistance driven by the pfdhfr triple mutant originated in Southeast Asia and spread to Africa, with possible local emergences. Our results are broadly consistent with these patterns while highlighting local heterogeneity. These dynamics are incorporated in the discussion section.

Reviewer response: Thank you for your response, this has been addressed in the discussion.

Results:
Figure 1:
- It is difficult to read some district names in the map. I would suggest changing the district name to a darker colour, and for small districts such as Kabale or Jinja, it may be useful to annotate the name and number outside of the map, and to label with an arrow to the district?
- It could also be useful to shade the colour of the filled in districts on the map in a gradient based on how many isolates

were obtained (this is similar though across most districts so may not change much).

Response: We thank the reviewer for these helpful suggestions. Figure 1 has been revised to improve readability, including clearer district labelling and adjustments to the map presentation.

Reviewer response: Thanks, this has now been corrected and Figure 1 is much easier to understand. However, Figure 2B onwards are all extremely pixelated and hard to interpret (Figure 2B – Figure 6). Please can these be amended to high resolution images.
Figure 2:
- The labelling is mixed up on the figure and the legend. Panel a shows the proportion of alternative alleles and the total number sequenced, panel b shows the WSAF at 146L and 581G. You should rewrite the figure legend.

- "The proportion of samples genotypes at each locus" is also incorrect – I think it is the number (not proportion) of samples genotyped with the proportion of variants in that set. Please correct if this is incorrect.

- "Genotypes were assigned to samples carrying alleles with a within-sample allele frequency (WSAF) > 0.05%" does not align with "Mutant alleles with WSAF < 1.0 and supported by <5 reads were excluded." - What was the WSAF cut off for identifying a genotype? WSAF =1? Or WSAF > 0.05? If the WSAF cut off was 1, you will be missing all alternative alleles in multiclonal samples. Can you explain why this was used as your cut off?
Response: We apologise for this error. In the revised manuscript, Figure 2 has been removed and replaced with a detailed description in the Methods section explaining how alleles were called. All other figures are carefully checked for errors and omissions.
Reviewer response: Thanks. This figure is now very pixelated, please amend to a high-resolution image.

Figure 3:
- For each district, at each time point, it would be good to know what number of isolates there is data for whilst interpreting this map. - For example, many districts in 2016-2017 are grey due to 'not detected' – if there are 50 samples here instead of 200 samples in the year later, this could just be due to sampling size.
Perhaps only sites with > X amount of data are included in the map, but this needs to be stated in the figure legend if so.
- Data for Kabale is not included in the map for 581G in years 2018-19 and 2022 – why is this?
Response: We thank the reviewer for this comment. To ease the interpretation of Figure 2 (previously Figure 3), we have included supplementary Table 2, which reports the prevalence for each district and survey period, including the total number of samples analysed, the estimated prevalence, and the corresponding 95% confidence intervals. This allows readers to directly assess how sample size may influence the patterns observed in the maps.
Reviewer response: Thanks. This figure is now very pixelated, please amend to a high-resolution image. Please state in the legend that the numbers of isolates sampled in each location can be found in the supplementary table.

COI analysis:
"These spatial and temporal trends in poly-genomic infections reflect fluctuating transmission, with evidence of reduced malaria transmission in later years in some sites."
Is this published evidence? If so, can you site it, or state if this is observations from individuals at the site in health clinics for example?
Response: We thank the reviewer for this suggestion. Although additional data could potentially support this statement, to avoid overinterpretation, we have decided to remove the COI-related analysis from the manuscript. Further justification for removing COI in our analysis can also be found in the response to minor comments from Reviewer #1.

Reviewer response: Thanks, this comment has now been addressed.

Figure 5/6:
In Figure 5, the colouring system for variants is: 164L – red, 581G – yellow, Wild-type – blue. Yet in Figure 6, the colouring system changes to 164L – yellow, 581G – grey, Wild-type – blue. Please can you keep a uniform colouring system where possible?
Response: We thank the reviewer for pointing out the need for proper figure colours and aesthetics. All figures have been regenerated with a consistent colouring scheme.
Reviewer response: Thanks, this comment has now been addressed.

Figure 7:
Due to the clustering of the dhfr 164L isolates, it is difficult to decipher the symbol where there are lots of isolates. Could the symbols of this plot be changed to look more distinctive between genotypes? Or could the network maps be larger, with one below the other, to allow for the points in the network to be more spaced apart?

Response: We thank you for this suggestion. Different IBD network plots have been regenerated to represent sites, genotypes and sampling time, using only colours for representation.

Reviewer response: Thanks, this comment has now been addressed.

Methods:
For IBD analysis, a heavily filtered dataset is used, please state the number of samples remaining in this filtered dataset that

are included in IBD analysis.
Additionally, a recent preprint compared IBD softwares on P. falciparum data demonstrated that hmmIBD has a lower false positive rate than isoRelate (https://elifesciences.org/reviewed-preprints/101924).
Could you also run hmmIBD on this dataset to determine if the same patterns are seen with IBD inferred from this software?

Response: The Results section has been updated to include the number of samples and SNPs used for IBD analysis (lines 137 – 139). We also repeated the IBD analysis using hmmIBD, as recommended.

Reviewer response: Thanks, this comment has now been addressed.

Reviewer #3

(Remarks to the Author)
The authors have made a substantial effort to address the reviews, and the manuscript is clearly improved. In particular, they have moderated the central claim from "clonal transmission and rapid expansion" to a more cautious interpretation centered on multiple local expansions, they have re-run the IBD analysis using hmmIBD-rs, they now include WT-mutant comparisons, and the presentation of PfDHFR I164L prevalence over time is much clearer than in the original submission.

However, I do not think the manuscript is yet ready for publication. The revision is meaningful, but several remaining interpretive issues, incomplete resolution of demographic confounding, and clear residual inconsistencies/errors require further revision. See my comments below.

Major comments
1. The authors say they have moved away from over-interpreting IBD, but the manuscript still leans too strongly toward selection in places where the evidence remains mixed. The revised manuscript is improved in that it no longer equates high IBD with strict clonality, and this is an appropriate correction. However, the text still sometimes moves too quickly from relatedness/local expansion to selection-based interpretation. For example, the site-specific IBD section suggests that the absence of comparable enrichment among PfDHPS A581G mutants and wild-type parasites indicates insufficient selection pressure to influence its evolution. This remains too strong, because the observed patterns may also reflect demographic structure, heterogeneous transmission, recombination, and sampling effects. The Discussion is improved and does now acknowledge recombination, drift, and bottlenecks, but the conclusions should still be framed more cautiously throughout.

2. The demographic/transmission-confounding issue is not fully resolved. A major concern was that elevated relatedness could reflect demographic processes rather than selection. The authors responded by removing COI-related analyses. This simplifies the manuscript, but it does not fully resolve the underlying concern. In effect, an important source of context has been removed rather than convincingly addressed. I therefore do not think the reviewer(s) concerns was fully answered.

3. The addition of WT–mutant IBD comparisons is an improvement and addresses an important omission from the first version. However, the manuscript still does not fully exploit these comparisons to show whether the mutant-mutant enrichment is truly distinct from broader background population structure. How does it look when using the mean/median relatedness to compare between within mutant and between mutant-WT relatedness? Similarly, the revised prevalence analysis is clearer, but the temporal modeling is still fairly simplistic given the visibly non-monotonic trajectories at some sites, especially Jinja.

4. The manuscript reports extremely large chi-square statistics and p-values for pairwise IBD categories; the figure legend then states that these should be interpreted as descriptive differences rather than formal hypothesis tests of independent observations. That caveat is appropriate, but the manuscript should then be more disciplined and consistent in how these results are described in the Results and Discussion.

Minor comments
Line 118: The manuscript reports for PfDHPS A581G an odds ratio of 0.93 with a 95% CI of 0.97–1.02 and $p = 0.161$. The point estimate lies outside the confidence interval, which is impossible and must be corrected.

Lines 147–168: Please define "Std. resid" at first use. I assume these are standardized residuals from the chi-square analysis, but this should be stated explicitly in the text and/or figure legend.

Line 150: "…relatedness was significant ($\chi^2 = 1,125$)…"; the p-value is missing and should be provided for consistency.

Line 159: The rationale for excluding moderate IBD values due to uncertainty is understandable, but then the manuscript should explain more clearly why the higher IBD range is considered sufficiently robust. At present, the thresholding approach appears somewhat arbitrary and discards potentially informative structure in the continuous data.

Line 182: Figures 5D–F are discussed before 5A–C in the text. This should be reordered.

Line 209: "showed slightly slow EHH decay than wild-type" should be "showed slightly slower EHH decay than wild-type."

Lines 216–223: The phrasing remains somewhat stronger than warranted by the evidence. Terms such as "adaptive

evolution" should be used more cautiously unless the authors can more clearly separate selective from demographic explanations.

Line 293: The sentence beginning "Overall, the data are consistent with a model…" is followed by broken text: "process is likely influenced by recombination…" This appears to be a drafting/editing error and should be corrected.

Lines 319 and 390: Please clarify more explicitly that the 100-kb flanking regions refer to the sequencing design, whereas the 50-kb region refers to the haplotype analysis window. This distinction is present but remains easy to misread.

Line 648 / Figure 5 legend: The legend appears incorrect. Panels B and E are by genotype, whereas C and F are by year. Please correct.

Reviewer #4

(Remarks to the Author)
Asua et al. report a genomic surveillance study of antifolate resistance in Plasmodium falciparum in Uganda from 2016–2022, analysing 4,725 isolates using MIPs sequnecing. Focusing on the PfDHFR I164L and pfdhps 581G mutations on a near-fixed quintuple-mutant background, the authors use identity-by-descent, haplotype networks, and extended haplotype homozygosity to characterize resistance dynamics. The study documents persistent circulation of pfdhps 581G and spatial heterogeneity and an increasing frequency of PfDHFR I164L in specific districts, including clusters of highly related parasites, providing insight into the evolution of antifolate resistance in Uganda. The results indicate a epidemic and geographically structured population, with local expansion of clusters of highly related parasites, including clusters with the I164L mutation. The results presented support the conclusion and discussion in the manuscript.

I reviewed only the 2nd version submitted with the rebuttal to the reviewers comments to the first version. It is my impression that a significant revision has been made compared to the initial version, with additional analysis and figures and revision of text and figures.

Major comments on COI were adressed by removing this analysis. Personally, I would prefer to have some (molecular) measure of transmission (be it COI, proportion of polyclonal infection, diversity, or other), to give more context, but I agree with the authors that it is not essential to answer the research questions, so it is OK like this.
Major comments on IBD were adressed by redoing the IBD analysis using HmmIBD and generating new figures.
Major comments on clonal expansion were adressed by rephrasing to local expansion, which I agree is more appropriate, and a more elaborate extended haplotype analysis. While the new figure 6 shows the overal haplotype composition, and highlights the I164L haplotypes in the table. However, it is still hard to interpret the temporal dynamics of these specific haplotypes as asked by reviewer #1. It might be of interest to add a figure with the proportion of the different haplotypes from the table in fig 6 compared to wt haplotypes over time (for example in a Sankey diagram); overall and by district.

The methods were described with sufficient clarity.

Several figures in the pdf version for the reviewer were low resolution (Fig 2B, 3, 4,5, 6A).

Minor comments
line 209: "showed slightly slow EHH decay than wildtype", should be "... slowER ... than .."
line 293: sentence starts with process, something seems missing here "This process"?

Open Access This Peer Review File is licensed under a Creative Commons Attribution 4.0 International License, which permits use, sharing, adaptation, distribution and reproduction in any medium or format, as long as you give appropriate credit to the original author(s) and the source, provide a link to the Creative Commons license, and indicate if changes were made.
In cases where reviewers are anonymous, credit should be given to 'Anonymous Referee' and the source.
The images or other third party material in this Peer Review File are included in the article's Creative Commons license, unless indicated otherwise in a credit line to the material. If material is not included in the article's Creative Commons license and your intended use is not permitted by statutory regulation or exceeds the permitted use, you will need to obtain permission directly from the copyright holder.
To view a copy of this license, visit https://creativecommons.org/licenses/by/4.0/

Reviewers' comments:

Reviewer #1 (Remarks to the Author):

Asua et al. use genomics to explore the spatiotemporal landscape of P. falciparum antifolate resistance across Uganda over an 8yr period. They focus on changes in PfDHFR I164L prevalence, which confers high-level pyrimethamine resistance. The emergence and changing prevalence of PfDHFR I164L in Uganda has been reported by the same authors in several previous publications (e.g. Tumwebaze et al. [2015] AAC; Tumwebaze et al. [2017] JID; Asua et al. [2019] AAC; Asua et al. [2021] JID); and the dataset used here has been published previously (I believe, the authors should clarify), with a focus on kelch13 markers (Conrad et al. [2023] NEJM). Here, the primary novelty is the presentation of additional genomic analyzes, performed to gain a deeper insight into signals of parasite relatedness and selection that may explain prevalence changes in PfDHFR I164L. Unfortunately, I found the main claims of the paper – i.e. a rapid clonal expansion of I164L – are not clearly and convincingly supported by the data; and there seem to be errors in the figures / text which have made reviewing the manuscript difficult.

Response: *We thank the reviewer for the constructive critique, and we apologise for the mislabeling of some figures. In the revised version of the manuscript, we have included re-analysis and interpretation of the findings. The key message was then revised accordingly, including revising the title from "Clonal transmission and rapid expansion of PfDHFR I164L shape the evolving antifolate resistance landscape in Plasmodium falciparum across Uganda" to "Multiple local expansions of PfDHFR I164L drive evolution of antifolate resistance in Plasmodium falciparum in Uganda". We have also paid keen attention to ensuring figures and texts are correctly labelled.*

Major Comments

1. A main claim of the manuscript is that PfDHFR I164L has increased in prevalence across Uganda. This is highlighted in the Abstract, lines 41-42: "PfDHFR I164L increased from 19% in 2016-17 to 34% in 2022". However, these years used different sample collection sites (10 vs 16, Methods, line 323) and so the trend is confounded by possible spatial heterogeneity. Was this controlled for in some way? If so, please describe this in the manuscript. If not, this should be controlled as, looking at the presented data, there does seem to be substantial spatial heterogeneity which could impact the magnitude/conclusion.

Response: *We are grateful for this important point regarding the key message of the manuscript and the potential for spatial confounding due to the sampling approach. In order to clarify sampling, we have revised the abstract (lines 34-36) and the Methods section (lines 306-314) to provide a more detailed description. We have also provide more detailed description of the prevalence data, and further supplemented the figures with supplementary Table 2.*

2. In addition to claiming that PfDHFR I164L is expanding in Uganda, the authors claim that this expansion is rapid (e.g. in the title). But the data do not seem to support this and, moreover, there are errors in data reporting that complicate interpretation. For example, in the Discussion lines 236-40: "The prevalence of PfDHFR I164L increased from 2016-17 to 2022 (Fig. 3), with particularly pronounced increases (more than threefold) at four sites outside southwestern Uganda (Arua, Amolatar, Jinja, and Lamwo; Supplementary Table 2)." First, Lamwo does not have increasing 164L in Fig 3 or in STable 1 (note STable 2 is also the wrong reference); this seems to be an error. Second, in STable 1, Aura's prevalence change was 10% (2016) to 20% (2022), which is only a two-fold change, and moreover the confidence intervals overlap. Third, for Amolatar, the small sample size in 2022 (8 samples) means the results should be interpreted with caution, as they could be extremely sensitive to sampling

bias. In addition, in Results, line 116: "Amolator 12% (6/50) to 100% (8/8)"; but in Fig. 3, this is not the case: the site is missing in 2022, and in 2021 the prevalence was <20%. For Jinja, the prevalence of PfDHFR I164L seems to fluctuate dramatically, e.g. 56% (2020), 1% (2021), 84% (2022) – there are major decreases and increases; not a consistent rapid increase. Moreover, later in the Discussion, lines 251-253, the authors state: "PfDHFR I164L and PfDHPS 581G have been at high prevalence in southwestern Uganda since the late 1990s35–37, and now show relatively stable prevalence…" Yet two sites with arguably the most steady and convincing increases in PfDHFR I164L are both in SW Uganda (Kabale and Kanungu). Given these points, I am not convinced that PfDHFR I164L is rapidly expanding outside of SW Uganda.

Response: *Thank you, and in response to the comments on over interpretations of the prevalence data, we performed a re-analysis and revised the description of the prevalence trends. We further clarify that, in addition to describing temporal trends, in the revised manuscript, we further examined the genetic architecture underlying the evolution of antifolate resistance. By focusing on the PfDHFR I164L mutation and integrating prevalence surveillance with population genomic analysis, we provide new insights into the evolutionary dynamics of antifolate resistance.*

3. To help address 2., the longitudinal data of PfDHFR I164L across Uganda needs to be much more clearly presented, given its centrality to the paper. Presently data is either in the map in Fig. 3 (which is quite hard to read, uses arbitrary prevalence bins, and doesn't show CIs) or buried in STable 1. To fix this, a plot of longitudinal trends of PfDHFR I164L for different sites and overall should be included in Fig. 3. It would also help to have supplementary tables specifically looking at PfDHFR I164L across years and sites (e.g. columns years, rows sites, values prevalence), since it is the main focus of the paper.

Response: *We thankfully agree with the above suggestion. Accordingly, we revised the presentation of PfDHFR I164L prevalence data to improve clarity. Figure 2 has been updated with a new panel (Figure 2B) that shows longitudinal trends at each site, including confidence intervals. While Supplementary Table 2 provides site-year specific numerical prevalence values. This allows readers to clearly assess temporal changes and site-specific patterns.*

4. In trying to look into comment 1. further, I found several inconsistencies / ambiguities in the presentations of the 164L maps. From Methods, line 323, there should be 10 collection sites in 2016-17, and 16 thereafter. Yet for I164L, Figure 3 displays 14 sites, which are shown across all years (except 2022, which has 12). Perhaps 'Not detected' means 'No data collected this year'? But if so: (i) why is there no bin for zero prevalence and (ii) why do sites disappear from 2021 to 2022? Maybe the Methods are wrong? Fig. 1 does show 16 sampling sites, but the administrative boundaries seem to differ from Fig. 3. This makes it hard to follow some of the Results text, which highlight specific sites (requiring finding the sites in Fig. 1, and then matching them to Fig. 3). These issues should be resolved, and more information should be included in figure legends to add clarity.

Response: *We are grateful for the critical review and the constructive feedback on Fig.1 and Fig. 3. For the maps in the revised manuscript, we generated new maps in Fig.1 and Fig. 2A (previously Fig. 3) with consistent labelling and boundaries. As such, the number of sites per year now accurately reflects the sampling scheme (10 sites in 2016-17, 16 thereafter), and sites with no data are clearly indicated. Figure legends and map annotations now specify zero-prevalence bins as well.*

5. In Results line 112, the claim is also made that the PfDHFR I164L mutation spread across sites from 2016-17 to 2022. This is important and interesting if true. However, looking at Fig. 3 the number of sites with >20% PfDHFR I164L varies, with no clear evidence of spread as far as I can tell. Similar to comment 2., the argument would benefit from numerical analysis supporting that PfDHFR I164L is spreading across Uganda (as distinct from increasing at specific sites).

Response: *We thank the reviewer for this observation. Upon re-analysis, we did not observe statistically significant evidence for geographical spread of PfDHFR I164L across sites. While visual inspection appears to suggest some site-to-site variation, the data do not provide definitive support for our previous claim of spatial spread. Accordingly, in our description of the prevalence patterns in the result section, lines 107-125, we have removed statements implying spread to multiple sites, but rather, we have revised the text to focus on site-specific prevalence trends.*

6. I am not sure that the analysis in Results 'Clonal transmission of the PfDHFR I164L allele' and in Fig. 4 provides evidence of clonal transmission. Given that the 164L mutation seems more geographically restricted than wild-type allele, simple isolation by distance (e.g. more distant populations are more genetically diverse)

would be sufficient to explain these observations. For example, the average distance between two random wild-type pairs will be farther than 164L pairs (I expect). The fact that most of the signal comes from Jinja seems to support this. To disentangle the effects of spatial distribution and clonal transmission, you would need to sample an equal number of wild-type pairs from the same sites as were sampled for PfDHFR I164L.

Response: *Whereas IBD sharing on its own is insufficient to demonstrate clonal expansion, sampling an equal number of wildtype and mutant from the parasite population to characterise it, in practice, is difficult because of high prevalence heterogeneity. However, to gain better insight into the evolution of the PfDHFR I164L mutation, we conducted a series of complementary analyses, including IBD sharing, IBD network, haplotype structure, and extended haplotype homozygosity (EHH) statistics. These provide a clearer assessment of the genetic background shaping the evolution of the PfDHFR I164L mutation.*

7. I am having some issues linking data in Fig. 5 with results. In particular, lines 154-156 state 100% of 164L pairs in Hoima have >0.5 IBD, but I see no 164L pairs in the figure. Similarly, I think there should be pairs for Jinja in 2020, and for Kabale, but none are visible. I am not sure if this is due to overplotting or something else, but it makes it hard for readers to independently assess the data. This data should be visualized more clearly, perhaps by binning IBD intervals and showing proportion of pairs falling within a given IBD bin.

Response: *We thank the reviewer for highlighting these visualisation pitfalls and suggesting an alternative approach. In the revised manuscript, we take into consideration your advice and revised the presentation of IBD sharing by binning them into categories representing different levels of relatedness and genotype Fig. 3 and Fig. 4). In the revised manuscript, we replaced Fig. 5 in the previous version with Fig. 3 and Fig. 5*

8. In the results section 'Differential patterns of PfDHFR I164L …' The high levels of pairwise IBD between I164L samples is used to suggest possible clonal expansion of I164L, with the implication being (in my reading) that it is under selection (e.g. lines 157-159, 168-169). But in general, genome-wide relatedness signals like these are more commonly indicators of demographic changes or structure, rather than selection, which acts on a specific locus. Moreover with P.f. malaria, low transmission alone can lead to clonal propagation of parasites due to a paucity of polyclonal infections, which are required for outcrossing in the mosquito; all in the absence of any sort of selection. Given that (i) several wild-type clusters with high-relatedness were observed, often in the same sites where 164L had high relatedness; and (ii) over half of sites had a decline in COI over the study period (suggesting low / reducing transmission) -- is this not much more likely than selection? On lines 267 the authors state that wild-type clonal propagation is 'paradoxical', but it is completely expected (and has been observed before, e.g. in Senegal), in cases where transmission is low or populations have decreased in size.

Response*: We agree that genome-relatedness (high IBD) can occur as a result of population structure and a reduction in effective population size. Accordingly, in the revised manuscript, our analyses provide a more detailed series of assessments of genetic backgrounds characterising PfDHFR I164L evolution. Figures 4 – 5 present these analyses, with interpretations detailed in the Discussion sections. All the results from the analyses are taken into account while interpreting our results. Specifically, we revised the previous inference of "clonal expansion" to a more general term, "local expansion".*

Minor Comments

• Are the samples and sequencing data used here the same as in Conrad et al. (2023) NEJM? If so, this needs to be clearly stated in the 'Study samples' section of the methods.

Response: *We thank the reviewer for this observation. The manuscript has been updated in the results section, lines 96-98 and 100, to clearly state that part of the results generated from sequencing of these samples was previously published.*

• Abstract, lines 43-44: "haplotype structure suggested multiple origins of PfDHFR I164L" and then Results, lines 186-187, in reference to 164L: "This pattern suggests a single origin several generations ago, …". These two interpretations of the data seem to contradict.

• Abstract 43-44: Why 'Although', aren't these findings consistent with each other?

Response: *We acknowledge that the terms "haplotype structure suggested multiple origins of PfDHFR I164L" and " This pattern suggests a single origin several generations ago,…" did represent an accurate intepretation of the data. Our data can not accurately differentiate multiple haplotype backgrounds due to independent origins from those generated by recombination. Therefore, in the revised manuscript, we have revised this interpretation to "multiple haplotype backgrounds"*

• I am struggling to understand how the genotypes were called. In the Methods lines 341-342, it says mixed genotypes were called if WSAF > 0.05. But in Fig. 2b, it looks by eye that there are mixed genotypes called with <0.05 (some points, esp. for 581G, are very close to 0.0 or 1.0), which would be inconsistent with the methods. Moreover, in Fig. 2b why are there 'mutant' genotypes with such low WSAF (e.g. < 0.9); should these not be mixed? How were homozygous mutant genotypes called? Are you naively using the genotype calls from FreeBayes, and then using WSAF as a post hoc filter? Or are you calling variant sites with FreeBayes, and then genotypes using the WSAF and depth? Overall, it is important the genotyping procedure is described in more detail in the Methods and Fig. 2 figure legend, as it impacts all downstream analyses.

Response: *We thank the reviewer for highlighting the need for clarity on genotype calling. The Methods section has been updated to describe this process in detail. Briefly, Unique molecular Identifier (UMI) based allele calling (lines 332–341) was used to determine allele presence, allowing distinction of true alleles from PCR or sequencing errors. Major alleles were identified using within-sample allele frequency (WSAF, lines 350–358), providing a quantitative measure of allele abundance.*

• Fig. 2 is just sequencing QC and should not be a main results figure; especially given there are already 8 main figures. Moreover, previous work from overlapping authors has shown that WSAF from MIPs are quite unreliable, I am not sure why it is being presented in a main figure (https://www.researchsquare.com/article/rs-5743980/v1).

Response: *Thank you for this suggestion. Figure 2, which primarily presented sequencing QC, has been removed, and quality control approaches (during genotype calling) are now described in the Methods section as stated above.*

• Note that lines 103-104 need rewording, as homozygous reference is also a genotype.

Response: *We thank the reviewers for their constructive feedback. The manuscript has been revised to address all major and minor comments, with clarifications in the Methods, Results, Figures, and Supplementary Materials.*

• The COI tool used (TheRealMcoil) assumes no within-host IBD, yet it seems a lot of these samples have IBD. The authors should remark on this in the manuscript, as the COI estimates may be biased downwards.

Response*: We thank the reviewer for this important observation. Since we did not explicitly assess within-host IBD, the assumptions in TherealMcoil may be violated, potentially biasing COI. Additionally, our analyses utilised UMI-based genotype calls and WSAF based dominant alleles for IBD and haplotype inference, making COI less informative for our questions. To avoid potential misinterpretation and information overload, COI has been excluded from the revised manuscript.*

• In Fig. 5, why does the proportion of IBD seem to hit a maximum at 0.8? What is being used to define the denominator of these proportions? This is confusing because presumably the same data is used to create the IBD network in Fig. 7 which requires IBD > 0.9.

Response: *We thank the reviewer for this important clarification. The apparent plateau at 0.8 in the previous figure 5 resulted from summarising IBD as the proportion of shared segments across pairwise comparisons, with the denominator defined as total comparable genomic segments per pair. However, we acknowledge the limitation of this approach and re-performed the IBD analysis using hmmIBD, and generated the proportion of genome-shared-IBD. All figures and text have been updated to ensure consistency, comparability, and clearer presentation.*

• For many of the IBD analyses, samples are divided into I164L, 581G, and WT; but these categories are not mutually exclusive. Were there 164L and 581G double mutants? How many, and how were they handled for the IBD and haplotype analyses?

Response: *Thank you for this important point. IBD analysis was performed for all samples regardless of their genotype status, and therefore, we have included comparisons for sample pairs with both 164L and 581G mutations and presented the summary result in Fig. 3 (lines 154-158). For haplotype analysis, we have used only isolates with the 164L mutation and a random subset of those wildtype for I164 (lines 197-199).*

• In Fig. 7, for many clusters (e.g. Jinja 2016) it is very hard to see the genotype because the points overlap. Maybe either it is better to use only color, and have three networks, one displaying genotypes? Or can you replot so the points don't overlap?

Response: *We thank the reviewer for this suggestion. The IBD network plots have been revised (Figure 5) and are now separated by site, genotype, and year, reducing point overlap and improving visualisation for clearer interpretation*

• Line 182: the section is called 'Expansion of multiple haplotypes of PfDHFR I164L', but no evidence is presented that multiple haplotypes have expanded. Evidence of 164L increase in prevalence was presented previously, but was this driven by frequency increase of specific haplotypes identified here? If so, can you present that data? If not, I would suggest renaming 'Multiple distinct haplotypes of PfDHFR I164L' or similar.

Response: *We thank the reviewer for this comment. The Results section has been fully revised to improve clarity and flow. Particularly in the results section (lines 197-205) describe haplotypes surrounding the PfDHFR I164L mutation.*

• In Fig. 8 and SFig. 3, can I confirm that the REF and ALT alleles are labelled correctly? As currently labelled, there are a large number of fixed ALT sites, which is atypical.

Response: *We thank the reviewer for raising the possibility of switched REF and ALT labels. After re-examination, we confirm the labels are correct: the REF allele corresponds to the P. falciparum 3D7 reference genome. Note that, in the revised manuscript, Fig. 8 from the previous version has been renamed Fig. 4.*

• Fig 8a: Please provide some labelling of the x-axis to give readers a sense of scale of the conserved region.

Response: *Fig. 8a, now renamed as Fig. 4, has been updated; the suggestions have been incorporated.*

• Lines 128-130: Do you have malaria prevalence or incidence data to support the claim that COI decline is evidence of reduced transmission in these sites? In theory COI correlates with transmission, but often this correlation is weak and confounded by other phenomenon (e.g. migration), and so can't be interpreted in this way so easily.

Response: *We do not have specific prevalence or incidence data to support the claim that a decline in COI directly correlates with reduced transmission at these sites. Further, as stated in our response to Reviewer-1, minor-comment on bullet six, COI has been entirely removed from the revised manuscript.*

• Lines 184-188: The authors claim that a shared core haplotype flanked by diversity is indicative of 'a single origin several generations ago, followed by recombination events that broke down the shared haplotype over time'. Can they clarify how big the core haplotype is in the text or figure? This claim can only be made if there was initially sufficient diversity present across this core haplotype. Otherwise, it is also possible that 164L emerged multiple times on the same core haplotype background. Given that PfDHFR has been under selection for a long time and resistance haplotypes have spread across Africa, this wouldn't be too surprising, no?

Response: *We thank the reviewer for this insightful comment. We apologise that, following re-analyses of our data, we have revised the section to describe the multiple haplotypes surrounding PfDHFR I164L.*

• Line 278: 'sulfamethoxazole-trimethoprim' and then line 295 'Trimethoprim-Sulfamethoxazole', please use consistent nomenclature.

Response: *Thank you for pointing out this inconsistency. The nomenclatures are revised and consistent throughout the manuscript.*

• For the authors awareness, in the PDF version of the manuscript the figures were very low resolution, and the word document was quite large (21Mb) and sometimes laggy.

Response: *We thank the reviewer for bringing this to our attention. The figures have now been replaced with lighter, higher-resolution versions to improve clarity and reduce file size.*

Reviewer #2 (Remarks to the Author):

Summary
Overall, this is an interesting and novel research paper, looking into variants in pfdhfr and pfdhps in >4000 field isolates from 16 sites in Uganda taken during the period of 2016-2022. Through genotyping of 11 SNP positions in pfdhfr/pfdhps, flanking regions surrounding the genes and SNPs from a genome wide panel, the manuscript investigates the prevalence of variants over time in addition to the selective pressures on these variants.
Both PfDHFR I164L and pfdhps 581G have been present in Uganda for a while, with some clonal transmission of PfDHFR I164L in distinct foci and the possibility of this variant arising from multiple origins, and a less clear picture for pfdhps 581G. The manuscript specifically shows clonal expansion of PfDHFR I164L geographically restricted to the area of Jinja, with signals of recent positive selection, which is important when considering malaria interventions in this region.

Overall comments:

1. Whilst documenting the presence of these variants in Uganda is not novel, there has not been a previous study looking into selection dynamics of these alleles at these sites, and this study uses time-series data and a large dataset to enable this investigation. It would be interesting to compare this data to other global sequence data of these genes to determine where variant alleles have likely been spread from.
Previous pfdhfr mutations have been shown to spread to Africa from Southeast Asia (https://www.science.org/doi/10.1126/science.1098876). Could the authors compare sequence data from a pool of Southeast Asian isolates around this locus to also determine geographical spread? Or if this already has been shown, could the authors please refer to this in their manuscript?

Response: *We thank the reviewer for this valuable suggestion. However, in our haplotype analysis, we identified multiple haplotypes surrounding the PfDHFR I164L variant, with no single haplotype being clearly dominant. Comparing these haplotypes to global sequences to trace their origin would be a complex undertaking, without a clear hypothesis or question to address. For the above reason, we respectively, reframed from performing global analysis.*

2. The sequence data used was generated using MIPs. The MIPs targeted drug resistance genes, regions flanking dhfr/dhps and “single-nucleotide polymorphisms (SNPs) distributed throughout the P. falciparum genome” Please can you expand on what these SNPs are? How many additional SNPs are covered? What regions are targeted to sequence these? Why are these chosen? Is it a previously published SNP panel?
I am also struggling to see the importance of these SNPs in the manuscript, and analysis of them specifically, were they used in popgen analysis? Where?

Response: *We thank the reviewer for raising these important questions. Detailed information on the MIP targets, including their importance, is now provided in the methods section, lines 316-331, including reference to key publications.*

3. There are instances in the text when italics are not used for genus-species names. Please can you correct this? *P. falciparum*, not P. falciparum.

Response: *We apologise for the inconsistencies in P. falciparum formatting. These have now been corrected throughout the manuscript.*

4. Sulfadoxine resistance, due to mutations in pfdhps, is known to have emerged multiple different times in Africa (https://pmc.ncbi.nlm.nih.gov/articles/PMC2661256/). Which is different to the triple dhfr mutant in Africa leading to pyrimethamine resistance (https://www.science.org/doi/10.1126/science.1098876) which originated in Southeast Asia and spread to Africa.

Your results have shown that isolates with the pfdhfr 146L mutation share more relatedness than isolates with the pfdhps 581G mutation, indicative of a clonal expansion of the 164L mutation in some regions, and multiple origins of the 581G mutation.
Please can you comment on this to contextualise your results in comparison to previous work looking at the spread of pfdhfr/pfdhps mutations? Do you think that these variants show a similar pattern to the spread of other resistant alleles in these genes?

Response: *We thank the reviewer for this insightful comment. Indeed, previous studies have shown distinct evolutionary histories of antifolate resistance, with sulfadoxine resistance associated with pfdhps mutations arising multiple times independently in Africa, whereas pyrimethamine resistance driven by the pfdhfr triple mutant originated in Southeast Asia and spread to Africa, with possible local emergences. Our results are broadly consistent with these patterns while highlighting local heterogeneity. These dynamics are incorporated in the discussion section.*

Results:
Figure 1:
- It is difficult to read some district names in the map. I would suggest changing the district name to a darker colour, and for small districts such as Kabale or Jinja, it may be useful to annotate the name and number outside of the map, and to label with an arrow to the district?
- It could also be useful to shade the colour of the filled in districts on the map in a gradient based on how many isolates were obtained (this is similar though across most districts so may not change much).

Response: *We thank the reviewer for these helpful suggestions. Figure 1 has been revised to improve readability, including clearer district labelling and adjustments to the map presentation.*

Figure 2:
- The labelling is mixed up on the figure and the legend. Panel a shows the proportion of alternative alleles and the total number sequenced, panel b shows the WSAF at 146L and 581G. You should rewrite the figure legend.

- “The proportion of samples genotypes at each locus” is also incorrect – I think it is the number (not proportion) of samples genotyped with the proportion of variants in that set. Please correct if this is incorrect.

- “Genotypes were assigned to samples carrying alleles with a within-sample allele frequency (WSAF) > 0.05%” does not align with “Mutant alleles with WSAF < 1.0 and supported by <5 reads were excluded.” - What was the WSAF cut off for identifying a genotype? WSAF =1? Or WSAF > 0.05? If the WSAF cut off was 1, you will be missing all alternative alleles in multiclonal samples. Can you explain why this was used as your cut off?

Response*: We apologise for this error. In the revised manuscript, Figure 2 has been removed and replaced with a detailed description in the Methods section explaining how alleles were called. All other figures are carefully checked for errors and omissions.*

Figure 3:
- For each district, at each time point, it would be good to know what number of isolates there is data for whilst interpreting this map. - For example, many districts in 2016-2017 are grey due to ‘not detected’ – if there are 50 samples here instead of 200 samples in the year later, this could just be due to sampling size.
Perhaps only sites with > X amount of data are included in the map, but this needs to be stated in the figure legend if so.
- Data for Kabale is not included in the map for 581G in years 2018-19 and 2022 – why is this?

Response: *We thank the reviewer for this comment. To ease the interpretation of Figure 2 (previously Figure 3), we have included supplementary Table 2, which reports the prevalence for each district and survey period, including the total number of samples analysed, the estimated prevalence, and the corresponding 95% confidence intervals. This allows readers to directly assess how sample size may influence the patterns observed in the maps.*

COI analysis:
“These spatial and temporal trends in poly-genomic infections reflect fluctuating transmission, with evidence of reduced malaria transmission in later years in some sites.”
Is this published evidence? If so, can you site it, or state if this is observations from individuals at the site in health clinics for example?

***Response:** We thank the reviewer for this suggestion. Although additional data could potentially support this statement, to avoid overinterpretation, we have decided to remove the COI-related analysis from the manuscript. Further justification for removing COI in our analysis can also be found in the response to minor comments from Reviewer #1.*

Figure 5/6:
In Figure 5, the colouring system for variants is: 164L – red, 581G – yellow, Wild-type – blue. Yet in Figure 6, the colouring system changes to 164L – yellow, 581G – grey, Wild-type – blue. Please can you keep a uniform colouring system where possible?

***Response:** We thank the reviewer for pointing out the need for proper figure colours and aesthetics. All figures have been regenerated with a consistent colouring scheme.*

Figure 7:
Due to the clustering of the dhfr 164L isolates, it is difficult to decipher the symbol where there are lots of isolates. Could the symbols of this plot be changed to look more distinctive between genotypes? Or could the network maps be larger, with one below the other, to allow for the points in the network to be more spaced apart?

***Response:** We thank you for this suggestion. Different IBD network plots have been regenerated to represent sites, genotypes and sampling time, using only colours for representation.*

Methods:
For IBD analysis, a heavily filtered dataset is used, please state the number of samples remaining in this filtered dataset that are included in IBD analysis.
Additionally, a recent preprint compared IBD softwares on P. falciparum data demonstrated that hmmIBD has a lower false positive rate than isoRelate (https://elifesciences.org/reviewed-preprints/101924).
Could you also run hmmIBD on this dataset to determine if the same patterns are seen with IBD inferred from this software?

***Response:** The Results section has been updated to include the number of samples and SNPs used for IBD analysis (lines 137 – 139). We also repeated the IBD analysis using hmmIBD, as recommended.*

Reviewer #3 (Remarks to the Author):

Asua et al. present a large-scale genomic surveillance analysis of Plasmodium falciparum antifolate resistance in Uganda from 2016–2022, focusing on the dynamics of PfDHFR I164L and pfdhps 581G on a near-fixed quintuple-mutant background. Using targeted MIP-based deep sequencing, the authors generate 4,725 field isolates and analyse antifolate-resistance haplotypes with identity-by-descent (IBD), haplotype networks, and extended haplotype homozygosity (EHH). The manuscript is clearly written, well structured, and supported by detailed visualizations.

The dataset is substantial, geographically and temporally rich, and the laboratory and sequencing components are technically sound. Integrating IBD, haplotype structure, and temporal allele-frequency trajectories provides valuable insight into antifolate-resistance evolution. The study documents regional persistence of pfdhps 581G and increasing prevalence of PfDHFR I164L, and identifies clusters of highly related 164L parasites.

However, several aspects of the interpretation require clearer explanation and additional analysis. The distinction between selection and demographic effects needs further clarification, especially regarding claims of clonal spread that are not fully supported by the IBD values shown. The absence of WT-mutant relatedness comparisons, the consequences of dominant-clone filtering in the context of high polygenicity, and the limited integration of COI and regional transmission patterns leave key conclusions only partially supported. In addition, prevalence trends are tested repeatedly without addressing multiplicity, and some policy statements extend beyond the evidence. These issues require clarification before firm conclusions can be drawn.

Major comments

1) The authors repeatedly interpret the elevated relatedness among PfDHFR I164L parasites as evidence of “clonal spread”. The underlying relatedness signal is indeed strong; PfDHFR I164L pairs show substantially higher IBD than wild-type pairs, and this is clearly visible in the relatedness distributions presented in the Results. The authors use IBD ≥0.9 as the operational definition of clonality. However, IBD ≥0.9 indicates very close

relatedness but is not, on its own, proof of single-clone transmission across space and time. Can the authors comment on this?

Response: *Thank you for raising this important conceptual point. We agree that an IBD threshold of ≥ 0.9 indicates very close relatedness but does not, on its own, demonstrate single-clone transmission across space and time. Accordingly, in the revised manuscript, we avoid using IBD as evidence of clonality and clarified this point in the Results and Discussions section. To better contextualise the elevated relatedness among PfDHFR I164L parasites, we conducted complementary analyses, including IBD-based clustering, local haplotype structure analysis, and extended haplotype homozygosity statistics. Collectively, these analyses enabled us to assess whether the expansion of PfDHFR I164L is driven by selection or whether other demographic factors may play a role.*

Figure 5 shows no pair with IBD = 1 (or very close to 1). Moreover, the proportion IBD data provided in the linked data file (Prop_IBD.csv; https://github.com/victorasua/Antifolate_Analysis/blob/main/AF_Evol/data/Prop_IBD.csv) indicate that the maximum IBD is 0.855419282552218, meaning that no sample pair in the dataset reaches the ≥0.9 threshold. So, I am a bit puzzled as to how Figures 4a/b and 7 were generated if this should show only pairs with IBD ≥0.9? If the dataset contains no pairs in this range, the logic behind the network shown in Figure 7 requires clarification, specifically the implicit claim of clonal transmission across space and time. As the authors mention, selection scans limited evidence of recent selection, maybe except for Jinja.

Response: *We thank you for carefully reviewing the shared data and for highlighting this inconsistency. In the initial analysis, proportions of IBD were calculated manually from shared segments; hence did not reach values >0.9. We acknowldge thos limitation, and in the revised manuscript, we recomputed IBD estimates using the hmmIBD pipeline, which directly estimates IBD probabilities. This change was made in response to both your observation and the second reviewer's recommendation. The updated IBD estimates and corresponding data files have been regenerated and shared, and all downstream analyses and figures have been updated accordingly. Importantly, in the interpretation of IBD networks, we no longer imply strict clonal transmission across space and time. Instead, clusters are interpreted as related lineages rather than fully clonal pairs, consistent with the overall modest evidence for soft selection.*

2) Adding to the above point, Figure 5 shows IBD sharing only for 164L pairs, 581G pairs and for wild-type pairs, but it does not display the IBD sharing for wild-type-mutant pairs. Why was this comparison omitted? Without the WT-mutant IBD sharing, it is difficult to determine whether the elevated relatedness reported among 164L parasites reflects locus-specific structuring driven by selection or instead mirrors the broader demographic background of the parasite population. Supplementary Table 3 shows that several regions with high 164L–164L IBD ≥0.5 also exhibit high WT-WT relatedness, consistent with demographic effects such as lower transmission, reduced recombination, or low COI. WT–mutant relatedness should be reported explicitly, either in Figure 5 or a separate figure/table.

Response: *Thank you for this valuable suggestion. We agree that explicitly reporting wild-type–mutant (WT–mutant) IBD sharing is essential for a complete understanding of the patterns of relatedness in the overall dataset. We have therefore extended our analysis to include WT–mutant pairwise comparison (Fig. 3).*

3) IBD and haplotype analyses are restricted to samples meeting the "dominant clone" threshold (WSAF ≥ 0.9 across 95% of loci), yet polygenomic infections comprise a substantial proportion of samples at many sites, meaning that a large share of the dataset is potentially excluded from population-genomic analysis. This approach risks biasing relatedness and haplotype-diversity estimates toward patterns typical of low-transmission, low-COI settings.

Response: *Thank you for this important point, which has prompted us to carefully consider our initial approach. Hence, we revised our analytical strategy to minimise data loss while limiting the influence of minor clones. Rather than excluding samples based on a genome-wide dominant-clone criterion (WSAF ≥ 0.9 across 95% of loci), we now apply allele-level filtering, retaining alleles with WSAF >75% and coding lower-frequency alleles as missing (lines 350 - 358). This approach allows inclusion of substantially more samples from polygenomic infections while reducing confounding from minor clones, thereby providing more representative estimates of relatedness and haplotype diversity across sites with differing transmission intensity.*

Figure 2 shows that a proportion of PfDHFR I164L samples have WSAF values below 0.9, implying that some 164L infections are polyclonal and therefore omitted from downstream analyses, potentially rendering the analysed subset unrepresentative of the full 164L-carrying population. The authors should report COI distributions by genotype (164L vs wild type), specify how many infections are strictly monoclonal (COI = 1), and assess whether elevated relatedness persists when analyses are limited to monoclonal infections. High-

relatedness regions appear to overlap with low-polyclonality regions (Supplementary Figs. 1–2), reinforcing the need for this assessment. Providing transmission-intensity context for each region would further strengthen interpretation.

Response: *We thank the reviewer for this suggestion. In the revised manuscript, resistance genotypes (including PfDHFR I164L) were determined using UMI-based allele calling rather than within-sample allele frequency (WSAF). By uniquely tagging original DNA molecules, this approach distinguishes true variants from PCR or sequencing artefacts and enables accurate detection of resistance alleles regardless of COI. Importantly, alleles were not excluded based on WSAF or COI, and genotype assignment was independent of COI. Therefore, analyses restricted to monoclonal infections are not required to interpret resistance prevalence. As noted in our response to Reviewer 2 (Minor Comment 7), and to avoid confusion about the role of COI, we have removed COI-related analyses from the revised manuscript.*

4) Prevalence changes are tested separately for sites and years without accounting for multiple comparisons. Several Results paragraphs refer to "significant increases" but do they also hold under correction? The manuscript also does not specify whether comparisons were only between 2016 and 2022 or across all years; in some regions, 164L prevalence decreases mid-series (especially 2021). Given the high proportion of polygenomic infections, the authors may also need to consider COI as a covariate when interpreting prevalence changes.

Response: *We thank the reviewer for raising these important points. In the revised manuscript, we reanalysed temporal changes in prevalence using site-specific regression models with year as a continuous variable, enabling assessment of trends across all sampled years rather than only comparing 2016 and 2022. As described in the Methods (lines 342–349), we used the p-value to test to assess for a significant change in linear trends, applying a Bonferroni correction to account for multiple comparisons.*

Minor comments

Line 4: Affiliations are not listed in increasing numerical order.

Response*: We thank the reviewer for noting this. The affiliations have now been reordered to appear in increasing numerical order.*

Line 42: I would suggest replacing "IBD revealed clonal transmission" with something like "IBD analysis identified closely related parasite clusters." To avoid over-interpretation (clonal would be an IBD of 1).

Response*: We thank the reviewer for this suggestion. In the revised manuscript, we have updated the language from "clonal transmission" to the more general terms, e.g. "local expansion***",** *to more accurately reflect the IBD and other analysis results and avoid over-interpretation.*

Line 43: The Abstract states that the authors find "limited evidence of recent selection," while the Results section later claims "evidence of recent positive selection" in Jinja based on slower EHH decay around 164L compared to wild type. At the same time, the Results paragraphs describing COI and IBD patterns emphasise that high IBD is widespread among wild-type parasites due to declining transmission, which can mimic signatures of selection. Maybe this can be rewritten into a single coherent interpretation or otherwise more clearly separated?

Response*: We thank the reviewer for this comment. In the revised manuscript, following additional data reanalysis, we have updated the text to reflect a cautious interpretation of our observations. Specifically, acknowledged that the absence of a selection signal does not imply that selection is not occurring at all. We argue that the results from our analysis could also mean that selection may be occurring, but its detection is obscured by the breakdown of haplotypes by high recombination rate (lines 247-257).*

Line 77: Missing "and" after "Rwanda,".

Response*: We thank the reviewer for noting this. We have reviewed the text to address errors and omissions.*

Line 104: Clarify whether the WSAF threshold is 0.05% or 0.05 (5%); it is inconsistent with Methods line 346.

***Response**: We thank the reviewer for highlighting this inconsistency. In the revised manuscript, we used a WSAF threshold of > 0.75 to filter dominant alleles for downstream population-genetic analyses. For genotyping, we no longer rely on a WSAF cut-off; instead, UMI-based allele calling is used to determine the presence or absence of alleles, as described in the Methods. This ensures accurate detection of resistance alleles, including in polyclonal infections.*

Line 127: I think it would be interesting to provide some prevalence estimates across the years and regions if available to see if the decline in COI could be, at least in part, due to overall decline in prevalence? Do you have some info on MOI estimates, rather than just binary info on polyclonality?

***Response**: We thank the reviewer for this suggestion. We do not have site-specific malaria prevalence or incidence data to support the claim that a decline in COI reflects reduced transmission. Accordingly, and for the reasons outlined in our responses above, COI-related analyses have been omitted from the manuscript.*

Line 129: Clarify whether prevalence data are available.

***Response**: We apologize that we do not have malaria prevalence data.*

Line 132: Italicize P. falciparum.

***Response**: All species names are italized*

Line 137: Consider reporting overall relatedness by region.

***Response**: We thank the reviewer for this suggestion. However, we think regional aggregation could lead to misinterpretation. Further, during site selection, regional representativeness was taken into consideration. We have retained reporting at the district level, which was chosen to ensure full geographic representativeness across Uganda. We believe this resolution provides sufficient detail for interpretation without introducing regional aggregation.*

Line 138: The origin of the number 94,637 pairs is unclear; 3,168 samples would yield (3,168*3,167)/2 = 5,016,528 pairwise comparisons. Please clarify which samples were analysed and provide some more details on those analyses.

***Response**: We thank the reviewer for pointing this out. Following data re-filtering and reanalysis of IBD, the number of samples included and the total number of pairwise comparisons have changed. The updated sample counts, total number of IBD pairs, and analytical methods are now fully described in the Results section, lines 135-145.*

Line 186: Here you suggest a single origin of the 164L haplotype, but in the Abstract and other sections you claim multiple origins of the 164L haplotype? So, you suggest its multiple independent emergences or a single emergence that then spread?

***Response**: We acknowledge that this interpretation may be accurate, as explicitly differentiating origins based on unique surrounding haplotypes can be confounded by recombination. Therefore, in the revised manuscript, we have revised this interpretation to "multiple haplotype backgrounds"*

Line 191 / Figure 8b: Text describes 72 cluster members; figure shows 73. Harmonize.

***Response**: We thank the reviewer for identifying this discrepancy. The text and Figure 5 (previously Figure 8) have now been harmonised, and the correct number of cluster members is consistently reported throughout the manuscript.*

Line 237: Any explanation as to why there seems to be a drop 164L prevalence in the year 2021 across multiple sites?

***Response**: The apparent decline in PfDHFR 164L prevalence in 2021 across multiple sites was noted, but we were unable to identify a plausible explanation for this pattern. We have therefore described it as an observation without a clear explanation.*

Line 239: The reference appears to be Supplementary Table 1.

***Response**: References to figures have been corrected.*

Line 271 / 295: Use consistent terminology for trimethoprim–sulfamethoxazole.
***Response**: In the revised manuscript, this has been corrected.*

Line 326: Some additional info on the sampling strategy would be good. Why those two months? How were the ~50 – 100 participants selected? Is this a random subset or did sampling continue until this number was reached? How representative are those samples?

***Response**: Details of the sampling strategy have been updated in the Methods section (lines 307–315). Briefly, samples were collected annually during the peak malaria season (June–July). At each facility, 2–5 participants were randomly sampled per day until the target sample size was reached (~50 per facility in 2016–2017 and up to 100 in 2018–2022), providing a representative subset of patients presenting with malaria during the peak transmission period.*

Line 342: How were polyclonal infections handled for prevalence of resistance-associated alleles? Mixed would count as WT and ALT if WSAF >0.05?

***Response**: Genotype calls for resistance-associated alleles were based on UMI counts**,** with alleles called present if supported by > 5 UMIs. In polyclonal or mixed infections, prevalence was calculated as the proportion of samples carrying the mutant allele (both pure mutant and mixed infections). This approach ensures that the presence of the mutant allele is captured regardless of within-sample allele frequency.*

Line 349: State whether COI estimation used the categorical method; provide number of SNPs and number of samples retained.

***Response**: As noted previously, COI estimation has been excluded from the revised manuscript for the reasons outlined above.*

Line 353: "a p-value > 0.05 considered as a significant monotonic change in prevalence". I think this should be < 0.05.

***Response**: We apologise for this typo. It should read: "a p-value < 0.05 is considered a significant monotonic change in prevalence". And these corrections are not effected in the revised manuscript.*

Line 355: Again, provide some more details on how many SNPs and samples were retained after filtering. Why was IBD ≥0.9 chosen for network analysis (or different, why was IBD ≥ 0.9 considered clonal and not "near clonal")?

***Response**: In lines 135 - 139 of the result section, we clarified the total number of samples and SNPs used for IBD analysis. For IBD network analysis, we have included both IBD ≥ 0.9 and IBD ≥ 0.5. Justification for this additional analysis can be found in the method section, lines 379 - 383*

Line 357: Verify whether allele-frequency threshold "≥0.01%" is correct.

***Response**: Filtering based on within-sample allele frequency was updated as reflected in the methods sections, lines 349 – 357.*

Line 372: Clarify inconsistency between 200 kb and 50 kb flanking regions. Above you mention 200 kb regions flanking pfdhfr/pfdhps and here 50kb flanking. So, this means ~50 kb up/down stream for each gene?

***Response**: The 200 kb refers to the genomic window targeted by MIP probes for sequencing regions flanking drug resistance genes. In contrast, the 50 kb refers to the flanking regions considered for haplotype structure and*

sharing analysis specifically around the pfdhfr gene. These distinctions are now described in the methods section (lines 316–323).

Line 565 / Figure 1: Could add the specific sites to the map.

Response: *Thank you for this suggestion. This was adopted into the figure.*

Line 605: Figure 2a/2b descriptions appear swapped; include sample counts for Fig. 2b.

Response*: In the revised manuscript, Figures 2a and 2b have been removed, as the information they contained is now fully described in the Methods section.*

Line 635: Provide sample counts for all panels or state them in the text.

Response: *We thank the reviewer for this suggestion. In the revised manuscript, we have included supplementary Tables 1 and 2 that provide more detailed numerical values, including sample counts.*

Line 652 / Figure 7: I would be curious to see this plot also at lower IBD thresholds? If the spread was purely regional, then I would also expect clustering by region/year at lower thresholds. Do the authors have any demographic data (e.g. household) of the study participants? This could also be an important factor to consider when looking at relatedness, as individuals from the same/nearby households might be more likely to be infected with the same/similar clones.

Response*: Thank you for this valuable suggestion. We have revised Fig. 7 (now Fig. 5) to include IBD networks for thresholds of ≥ 0.5 (Fig. 5A–C) and ≥ 0.9 (Fig. 5D–F). Regarding demographic data, we did not have household-level information for the study participants. However, we recognise that individuals from the same or nearby households might be more likely to be infected with similar clones, and this could be an important factor to consider in future analyses of relatedness.*

Line 656: “First siblings”? Suggest using “full siblings” instead?

Response*: In the revised manuscript, we have removed the term “first siblings” and instead focus on describing the degree of IBD sharing directly.*

Line 677: Why are there nodes that are not connected to any other sample? I the legend you state, that the IBD network shows clonal pairs (IBD ≥ 0.9), which should mean that each node is connected to at least one other node, no (otherwise it’s not a pair)?

Response*: Unconnected nodes in the IBD network represent isolates that do not share IBD above the thresholds with any other isolate in the dataset being plotted. The figure legend now carries this clarification.*

Line 715: Define iR, which is not introduced earlier.

Response*: In the revised manuscript, the term "iR" has been removed, as it is no longer used.*

REVIEWERS' COMMENTS

Reviewer #2 (Remarks to the Author):

Summary
Overall, this is an interesting and novel research paper, looking into variants in pfdhfr and pfdhps in >4000 field isolates from 16 sites in Uganda taken during the period of 2016-2022. Through genotyping of 11 SNP positions in pfdhfr/pfdhps, flanking regions surrounding the genes and SNPs from a genome wide panel, the manuscript investigates the prevalence of variants over time in addition to the selective pressures on these variants. Both PfDHFR I164L and pfdhps 581G have been present in Uganda for a while, with some clonal transmission of PfDHFR I164L in distinct foci and the possibility of this variant arising from multiple origins, and a less clear picture for pfdhps 581G. The manuscript specifically shows clonal expansion of PfDHFR I164L geographically restricted to the area of Jinja, with signals of recent positive selection, which is important when considering malaria interventions in this region.

Overall comments:

1. Whilst documenting the presence of these variants in Uganda is not novel, there has not been a previous study looking into selection dynamics of these alleles at these sites, and this study uses time-series data and a large dataset to enable this investigation. It would be interesting to compare this data to other global sequence data of these genes to determine where variant alleles have likely been spread from.
Previous pfdhfr mutations have been shown to spread to Africa from Southeast Asia (https://www.science.org/doi/10.1126/science.1098876). Could the authors compare sequence data from a pool of Southeast Asian isolates around this locus to also determine geographical spread? Or if this already has been shown, could the authors please refer to this in their manuscript?
Response: We thank the reviewer for this valuable suggestion. However, in our haplotype analysis, we identified multiple haplotypes surrounding the PfDHFR I164L variant, with no single haplotype being clearly dominant. Comparing this complex set of haplotypes to global sequences to trace their origins would be a complex undertaking, without a clear hypothesis or question to address. Therefore, we refrained from performing global analysis.
Reviewer response: Thanks, agreed with the author that comparing the 17 I164L haplotypes described here to Southeast Asian sequence data would be a complex undertaking. However I do think it would be worthwhile to describe these results in the context of where I164L has been seen before e.g. a publication from 2010 (Origins of the Recent Emergence of Plasmodium falciparum Pyrimethamine Resistance Alleles in Madagascar, Andriantsoanirina, V. et al. 2010) has previously described that I164L is likely to have occurred on multiple occasions – has this been shown in southeast asia too? Perhaps this could be expanded in the discussion. I think the discussion hints that in southeast Asia there is one clonal expansion of the I164L allele due to hard selective sweeps, can you directly state if we also see I164L on multiple different genomic backgrounds in southeast asia too, or is there something specific also about this variant that increases the chances of it arising on multiple different genetic backgrounds.

RESPONSE TO SECOND REVIEW COMMENT: THANK YOU FOR THIS SUGGESTION. AN ADDITIONAL ANALYSIS BASED ON A SUGGESTION FROM REVIEWER-4 SHOWED TWO DOMINANT HAPLOTYPES (LINES 224 – 230; TRACKED VERSION). IN ADDITION, A FURTHER LITERATURE SEARCH FOUND EVIDENCE OF MULTIPLE EMERGENCES OF PFDHFR I164L IN KENYA, A LOGICAL COMPARATOR FOR UGANDA. AS SUCH, WE HAVE UPDATED THE DISCUSSION SECTION IN PARAGRAPH STARTING AT LINE 257, WHICH NOW READS "…MORE THAN ONE DOMINANT PFDHFR I164L-ASSOCIATED HAPLOTYPE BACKGROUND, CONSISTENT WITH MULTIPLE ORIGINS OF THE MUTATION. THIS FINDING IS CONSISTENT WITH PREVIOUS EVIDENCE FOR MULTIPLE INDEPENDENT EMERGENCES OF PFDHFR I164L IN KENYA".

The sequence data used was generated using MIPs. The MIPs targeted drug resistance genes, regions flanking dhfr/dhps and "single-nucleotide polymorphisms (SNPs) distributed throughout the P. falciparum genome" Please can you expand on what these SNPs are? How many additional SNPs are covered? What regions are targeted to sequence these? Why are these chosen? Is it a previously published SNP panel? I am also struggling to see the importance of these SNPs in the manuscript, and analysis of them specifically, were they used in popgen analysis? Where?
Response: We thank the reviewer for raising these important questions. Detailed information on the MIP targets, including their importance, is now provided in the methods section, lines 316-331, including reference to key publications.

Reviewer response: Thank you, this comment has been addressed.

2. There are instances in the text when italics are not used for genus-species names. Please can you correct this? P. falciparum, not P. falciparum.
Response: We apologise for the inconsistencies in P. falciparum formatting. These have now been corrected throughout the manuscript.
Reviewer response: Thank you, this comment has been addressed.

3. Sulfadoxine resistance, due to mutations in pfdhps, is known to have emerged multiple different times in Africa (https://pmc.ncbi.nlm.nih.gov/articles/PMC2661256/). Which is different to the triple dhfr mutant in Africa leading to pyrimethamine resistance (https://www.science.org/doi/10.1126/science.1098876) which originated in Southeast Asia and spread to Africa.
Your results have shown that isolates with the pfdhfr 146L mutation share more relatedness than isolates with the pfdhps 581G mutation, indicative of a clonal expansion of the 164L mutation in some regions, and multiple origins of the 581G mutation.
Please can you comment on this to contextualise your results in comparison to previous work looking at the spread of pfdhfr/pfdhps mutations? Do you think that these variants show a similar pattern to the spread of other resistant alleles in these genes?
Response: We thank the reviewer for this insightful comment. Indeed, previous studies have shown distinct evolutionary histories of antifolate resistance, with sulfadoxine resistance associated with pfdhps mutations arising multiple times independently in Africa, whereas pyrimethamine resistance driven by the pfdhfr triple mutant originated in Southeast Asia and spread to Africa, with possible local emergences. Our results are broadly consistent with these patterns while highlighting local heterogeneity. These dynamics are incorporated in the discussion section.

Reviewer response: Thank you for your response, this has been addressed in the discussion.

Results:
Figure 1:
- It is difficult to read some district names in the map. I would suggest changing the district name to a darker colour, and for small districts such as Kabale or Jinja, it may be useful to annotate the name and number outside of the map, and to label with an arrow to the district?
- It could also be useful to shade the colour of the filled in districts on the map in a gradient based on how many isolates were obtained (this is similar though across most districts so may not change much).

Response: We thank the reviewer for these helpful suggestions. Figure 1 has been revised to improve readability, including clearer district labelling and adjustments to the map presentation.

Reviewer response: Thanks, this has now been corrected and Figure 1 is much easier to understand. However, Figure 2B onwards are all extremely pixelated and hard to interpret (Figure 2B – Figure 6). Please can these be amended to high resolution images.

RESPONSE TO SECOND REVIEW COMMENT: *WE HAVE PROVIDED A HIGHER RESOLUTION VERSION OF THE FIGURE.*

Figure 2:
- The labelling is mixed up on the figure and the legend. Panel a shows the proportion of alternative alleles and the total number sequenced, panel b shows the WSAF at 146L and 581G. You should rewrite the figure legend.

- “The proportion of samples genotypes at each locus” is also incorrect – I think it is the number (not proportion) of samples genotyped with the proportion of variants in that set. Please correct if this is incorrect.

- “Genotypes were assigned to samples carrying alleles with a within-sample allele frequency (WSAF) > 0.05%” does not align with “Mutant alleles with WSAF < 1.0 and supported by <5 reads were excluded.” - What was the WSAF cut off for identifying a genotype? WSAF =1? Or WSAF > 0.05? If the WSAF cut off was 1, you will be missing all alternative alleles in multiclonal samples. Can you explain why this was used as your cut off?

Response: We apologise for this error. In the revised manuscript, Figure 2 has been removed and replaced with a detailed description in the Methods section explaining how alleles were called. All other figures are carefully checked for errors and omissions.

Reviewer response: Thanks. This figure is now very pixelated, please amend to a high-resolution image.

RESPONSE TO SECOND REVIEW COMMENT: *WE HAVE PROVIDED A HIGHER RESOLUTION VERSION OF THE FIGURE.*

Figure 3:
- For each district, at each time point, it would be good to know what number of isolates there is data for whilst interpreting this map. - For example, many districts in 2016-2017 are grey due to 'not detected' – if there are 50 samples here instead of 200 samples in the year later, this could just be due to sampling size.
Perhaps only sites with > X amount of data are included in the map, but this needs to be stated in the figure legend if so.
- Data for Kabale is not included in the map for 581G in years 2018-19 and 2022 – why is this?
Response: We thank the reviewer for this comment. To ease the interpretation of Figure 2 (previously Figure 3), we have included supplementary Table 2, which reports the prevalence for each district and survey period, including the total number of samples analysed, the estimated prevalence, and the corresponding 95% confidence intervals. This allows readers to directly assess how sample size may influence the patterns observed in the maps.

Reviewer response: Thanks. This figure is now very pixelated, please amend to a high-resolution image. Please state in the legend that the numbers of isolates sampled in each location can be found in the supplementary table.

RESPONSE TO SECOND REVIEW COMMENT: WE THANK THE REVIEWER FOR THIS COMMENT. WE HAVE PROVIDED A HIGHER RESOLUTION VERSION OF FIGURE 2 (WHICH WAS FIGURE 3 IN THE FIRST MANUSCRIPT VERSION). WE HAVE UPDATED THE FIGURE LEGEND TO INCLUDE THE STATEMENT "THE NUMBER OF ISOLATES SAMPLED PER SITE CAN BE FOUND IN SUPPLEMENTARY TABLE 1".

COI analysis:
"These spatial and temporal trends in poly-genomic infections reflect fluctuating transmission, with evidence of reduced malaria transmission in later years in some sites."
Is this published evidence? If so, can you site it, or state if this is observations from individuals at the site in health clinics for example?
Response: We thank the reviewer for this suggestion. Although additional data could potentially support this statement, to avoid overinterpretation, we have decided to remove the COI-related analysis from the manuscript. Further justification for removing COI in our analysis can be found in the response to minor comments from Reviewer #1.

Reviewer response: Thanks, this comment has now been addressed.

Figure 5/6:
In Figure 5, the colouring system for variants is: 164L – red, 581G – yellow, Wild-type – blue. Yet in Figure 6, the colouring system changes to 164L – yellow, 581G – grey, Wild-type – blue. Please can you keep a uniform colouring system where possible?
Response: We thank the reviewer for pointing out the need for proper figure colours and aesthetics. All figures have been regenerated with a consistent colouring scheme.
Reviewer response: Thanks, this comment has now been addressed.

Figure 7:
Due to the clustering of the dhfr 164L isolates, it is difficult to decipher the symbol where there are lots of isolates. Could the symbols of this plot be changed to look more distinctive between genotypes? Or could the network maps be larger, with one below the other, to allow for the points in the network to be more spaced apart?

Response: We thank you for this suggestion. Different IBD network plots have been regenerated to represent sites, genotypes and sampling time, using only colours for representation.

Reviewer response: Thanks, this comment has now been addressed.

Methods:
For IBD analysis, a heavily filtered dataset is used, please state the number of samples remaining in this filtered dataset that are included in IBD analysis.
Additionally, a recent preprint compared IBD softwares on P. falciparum data demonstrated that hmmIBD has a lower false positive rate than isoRelate (https://elifesciences.org/reviewed-preprints/101924).
Could you also run hmmIBD on this dataset to determine if the same patterns are seen with IBD inferred from this software?

Response: The Results section has been updated to include the number of samples and SNPs used for IBD analysis (lines 137 – 139). We also repeated the IBD analysis using hmmIBD, as recommended.

Reviewer response: Thanks, this comment has now been addressed.

Reviewer #3 (Remarks to the Author):

The authors have made a substantial effort to address the reviews, and the manuscript is clearly improved. In particular, they have moderated the central claim from “clonal transmission and rapid expansion” to a more cautious interpretation centered on multiple local expansions, they have re-run the IBD analysis using hmmIBD-rs, they now include WT-mutant comparisons, and the presentation of PfDHFR I164L prevalence over time is much clearer than in the original submission.

However, I do not think the manuscript is yet ready for publication. The revision is meaningful, but several remaining interpretive issues, incomplete resolution of demographic confounding, and clear residual inconsistencies/errors require further revision. See my comments below.

Major comments

1. The authors say they have moved away from over-interpreting IBD, but the manuscript still leans too strongly toward selection in places where the evidence remains mixed. The revised manuscript is improved in that it no longer equates high IBD with strict clonality, and this is an appropriate correction. However, the text still sometimes moves too quickly from relatedness/local expansion to selection-based interpretation. For example, the site-specific IBD section suggests that the absence of comparable enrichment among PfDHPS A581G mutants and wild-type parasites indicates insufficient selection pressure to influence its evolution. This remains too strong, because the observed patterns may also reflect demographic structure, heterogeneous transmission, recombination, and sampling effects. The Discussion is improved and does now acknowledge recombination, drift, and bottlenecks, but the conclusions should still be framed more cautiously throughout.

RESPONSE TO SECOND REVIEW COMMENT: WE THANK THE REVIEWER FOR THIS CONSTRUCTIVE COMMENT. WE AGREE THAT SOME INTERPRETATIONS OF THE IBD ANALYSES WERE OVERLY DEFINITIVE AND HAVE REVISED OUR INTERPRETATIONS TO USE MORE CAUTIOUS LANGUAGE. FOR EXAMPLE, IN THE PARAGRAPH STARTING IN LINE 248, WE USED STATEMENTS SUCH AS …” CONSISTENT WITH SPATIALLY HETEROGENEOUS EVOLUTIONARY PROCESSES, INCLUDING ONGOING LOW-LEVEL SELECTION”, TO REFLECT THE COMPLEX EVOLUTIONARY DYNAMICS. ALL CHANGES ARE HIGHLIGHTED WITH TRACK CHANGES IN OUR REVISED MANUSCRIPT.

2. The demographic/transmission-confounding issue is not fully resolved. A major concern was that elevated relatedness could reflect demographic processes rather than selection. The authors responded by removing COI-related analyses. This simplifies the manuscript, but it does not fully resolve the underlying concern. In effect, an important source of context has been removed rather than convincingly addressed. I therefore do not think the reviewer(s) concerns was fully answered.

RESPONSE TO SECOND REVIEW COMMENT: WE THANK THE REVIEWER FOR HIGHLIGHTING THE POTENTIAL CONFOUNDING BETWEEN PARASITE RELATEDNESS, DEMOGRAPHIC STRUCTURE, AND TRANSMISSION INTENSITY. WE AGREE THAT ELEVATED RELATEDNESS MAY REFLECT BOTH EVOLUTIONARY PROCESSES AND LOCAL TRANSMISSION DYNAMICS. TO DIRECTLY ADDRESS THIS CONCERN, WE PERFORMED BAYESIAN LOGISTIC MIXED-EFFECTS MODELS INCORPORATING PFDHFR I164L AND PFDHPS A581G GENOTYPE PAIRING AS FIXED EFFECTS, STANDARDISED AVERAGE COMPLEXITY OF INFECTION (AVGCOI) AS A MEASURE OF WITHIN-HOST PARASITE DIVERSITY, AND SAMPLING SITE AS A RANDOM INTERCEPT TO ACCOUNT FOR UNMEASURED SITE-LEVEL DEMOGRAPHIC AND TRANSMISSION HETEROGENEITY (LINES 431–444). AFTER ACCOUNTING FOR COI AND SITE-LEVEL VARIATION, PFDHFR I164L GENOTYPE REMAINED STRONGLY ASSOCIATED WITH HIGH IBD, INDICATING THAT THE OBSERVED ASSOCIATION BETWEEN I164L AND PARASITE RELATEDNESS WAS NOT FULLY EXPLAINED BY DIFFERENCES IN WITHIN-HOST PARASITE DIVERSITY OR SITE-LEVEL POPULATION STRUCTURE. THE CORRESPONDING RESULTS HAVE BEEN ADDED TO THE RESULTS SECTION (LINES 162–179), AND THE DISCUSSION HAS BEEN REVISED TO INCORPORATE THE ROLES OF TRANSMISSION STRUCTURE, SITE-LEVEL HETEROGENEITY, AND COMPLEXITY OF INFECTION IN SHAPING OBSERVED PATTERNS (LINES 264–271; 284–290; 313–317).

3. The addition of WT–mutant IBD comparisons is an improvement and addresses an important omission from the first version. However, the manuscript still does not fully exploit these comparisons to show whether the mutant-mutant enrichment is truly distinct from broader background population structure. How does it look when using the mean/median relatedness to compare between within mutant and between mutant-WT relatedness? Similarly, the revised prevalence analysis is clearer, but the temporal modeling is still fairly simplistic given the visibly non-monotonic trajectories at some sites, especially Jinja.

RESPONSE TO SECOND REVIEW COMMENT: WE THANK THE REVIEWER FOR THIS SUGGESTION. TO FURTHER EVALUATE WHETHER THE OBSERVED MUTANT–MUTANT ENRICHMENT COULD BE

EXPLAINED BY UNDERLYING DEMOGRAPHIC OR TRANSMISSION STRUCTURE, WE INCORPORATED COI AND SITE-LEVEL VARIATION IN BAYESIAN LOGISTIC MIXED-EFFECTS MODELS (LINES 431–444). AFTER ACCOUNTING FOR THESE FACTORS, PFDHFR I164L GENOTYPE PAIRING REMAINED STRONGLY ASSOCIATED WITH HIGH IBD, SUPPORTING A GENOTYPE-ASSOCIATED ENRICHMENT OF RELATED PARASITE LINEAGES.

WE ALSO AGREE THAT PREVALENCE TRAJECTORIES WERE NOT UNIFORMLY MONOTONIC. GIVEN THE LIMITED NUMBER OF SAMPLING TIME POINTS PER SITE, WE RETAINED LINEAR REGRESSION AS A SUMMARY OF OVERALL TEMPORAL TRENDS BUT REVISED THE RESULTS AND DISCUSSION TO EXPLICITLY ACKNOWLEDGE NON-MONOTONIC TRAJECTORIES, PARTICULARLY AT JINJA (LINES 119–120, TRACKED VERSION), AND TO DISCUSS THE POTENTIAL CONTRIBUTIONS OF SELECTION, DEMOGRAPHIC STOCHASTICITY, AND TRANSMISSION BOTTLENECKS.

4. The manuscript reports extremely large chi-square statistics and p-values for pairwise IBD categories; the figure legend then states that these should be interpreted as descriptive differences rather than formal hypothesis tests of independent observations. That caveat is appropriate, but the manuscript should then be more disciplined and consistent in how these results are described in the Results and Discussion.

RESPONSE TO SECOND REVIEW COMMENT: *WE THANK THE REVIEWER FOR HIGHLIGHTING THIS ISSUE. WE HAVE REVISED THE RESULTS AND DISCUSSION TO CONSISTENTLY PRESENT THE IBD CATEGORY COMPARISONS AS DESCRIPTIVE RATHER THAN FORMAL TESTS OF INDEPENDENT OBSERVATIONS. THROUGHOUT THE MANUSCRIPT, RESULTS FROM COMPLEMENTARY ANALYSES ARE INTERPRETED TOGETHER TO PROVIDE A COHERENT PICTURE OF THE EVOLUTIONARY PATTERNS, RATHER THAN AS STANDALONE EVIDENCE (e.g., LINES 255–261).*

Minor comments

Line 118: The manuscript reports for PfDHPS A581G an odds ratio of 0.93 with a 95% CI of 0.97–1.02 and p = 0.161. The point estimate lies outside the confidence interval, which is impossible and must be corrected.

RESPONSE TO SECOND REVIEW COMMENT: *CORRECTED*

Lines 147–168: Please define “Std. resid” at first use. I assume these are standardised residuals from the chi-square analysis, but this should be stated explicitly in the text and/or figure legend.

RESPONSE TO SECOND REVIEW COMMENT: *CORRECTED*

Line 150: “…relatedness was significant (χ^2 = 1,125)…”; the p-value is missing and should be provided for consistency.

RESPONSE TO SECOND REVIEW COMMENT: *CORRECTED (LINE 155, TRACKED VERSION)*

Line 159: The rationale for excluding moderate IBD values due to uncertainty is understandable, but then the manuscript should explain more clearly why the higher IBD range is considered sufficiently robust. At present, the thresholding approach appears somewhat arbitrary and discards potentially informative structure in the continuous data.

RESPONSE TO SECOND REVIEW COMMENT: *CORRECTED (NOW IN LINES 144 - 146, TRACKED VERSION)*

Line 182: Figures 5D–F are discussed before 5A–C in the text. This should be reordered.

RESPONSE TO SECOND REVIEW COMMENT: *CORRECTED (NOW IN LINES 202 - 205, TRACKED VERSION)*

Line 209: “showed slightly slow EHH decay than wild-type” should be “showed slightly slower EHH decay than wild-type.”

RESPONSE TO SECOND REVIEW COMMENT: *THANKS, CORRECTED (LINE 234, TRACKED VERSION)*

Lines 216–223: The phrasing remains somewhat stronger than warranted by the evidence. Terms such as “adaptive evolution” should be used more cautiously unless the authors can more clearly separate selective from demographic explanations.

RESPONSE TO SECOND REVIEW COMMENT: *THROUGHOUT THE MANUSCRIPT, WE HAVE MODERATED INTERPRETATIONS TO AVOID ATTRIBUTING OBSERVED GENOMIC PATTERNS TO SELECTION ALONE, INSTEAD EMPHASISING SPATIALLY HETEROGENEOUS EVOLUTIONARY PROCESSES AND ACKNOWLEDGING CONTRIBUTIONS FROM SELECTION, RECOMBINATION,*

TRANSMISSION HETEROGENEITY, AND DEMOGRAPHY. AN EXAMPLE CAN BE FOUND IN LINES 255 – 257, WHERE WE HAVE USED TERMS SUCH AS "…. CONSISTENT WITH SPATIALLY HETEROGENEOUS EVOLUTIONARY PROCESSES, INCLUDING ONGOING LOW-LEVEL SELECTION". ALL CHANGES ARE HIGHLIGHTED WITH TRACK CHANGES IN OUR REVISED MANUSCRIPT.

Line 293: The sentence beginning "Overall, the data are consistent with a model…" is followed by broken text: "process is likely influenced by recombination…" This appears to be a drafting/editing error and should be corrected.

RESPONSE TO SECOND REVIEW COMMENT: WE THANK THE REVIEWER FOR IDENTIFYING THIS EDITING ERROR. THE SENTENCE IS NOW CORRECTED (LINE 296, TRACKED VERSION).

Lines 319 and 390: Please clarify more explicitly that the 100-kb flanking regions refer to the sequencing design, whereas the 50-kb region refers to the haplotype analysis window. This distinction is present but remains easy to misread.

RESPONSE TO SECOND REVIEW COMMENT: WE HAVE REVISED THE METHODS TO CLEARLY DISTINGUISH THE 100-KB FLANKING REGIONS TARGETED BY THE MIP SEQUENCING DESIGN FROM THE 50-KB WINDOW USED FOR HAPLOTYPE ANALYSIS, TO AVOID ANY AMBIGUITY (LINES 368-370 AND 451-452, TRACKED VERSION).

Line 648 / Figure 5 legend: The legend appears incorrect. Panels B and E are by genotype, whereas C and F are by year. Please correct.

RESPONSE TO SECOND REVIEW COMMENT: THANK YOU. THIS HAS BEEN CORRECTED.

Reviewer #4 (Remarks to the Author):

Asua et al. report a genomic surveillance study of antifolate resistance in Plasmodium falciparum in Uganda from 2016–2022, analysing 4,725 isolates using MIPs sequnecing. Focusing on the PfDHFR I164L and pfdhps 581G mutations on a near-fixed quintuple-mutant background, the authors use identity-by-descent, haplotype networks, and extended haplotype homozygosity to characterize resistance dynamics. The study documents persistent circulation of pfdhps 581G and spatial heterogeneity and an increasing frequency of PfDHFR I164L in specific districts, including clusters of highly related parasites, providing insight into the evolution of antifolate resistance in Uganda. The results indicate a epidemic and geographically structured population, with local expansion of clusters of highly related parasites, including clusters with the I164L mutation. The results presented support the conclusion and discussion in the manuscript.

I reviewed only the 2nd version submitted with the rebuttal to the reviewers comments to the first version. It is my impression that a significant revision has been made compared to the initial version, with additional analysis and figures and revision of text and figures.

Major comments on COI were adressed by removing this analysis. Personally, I would prefer to have some (molecular) measure of transmission (be it COI, proportion of polyclonal infection, diversity, or other), to give more context, but I agree with the authors that it is not essential to answer the research questions, so it is OK like this.

Major comments on IBD were adressed by redoing the IBD analysis using HmmIBD and generating new figures. Major comments on clonal expansion were adressed by rephrasing to local expansion, which I agree is more appropriate, and a more elaborate extended haplotype analysis. While the new figure 6 shows the overal haplotype composition, and highlights the I164L haplotypes in the table. However, it is still hard to interpret the temporal dynamics of these specific haplotypes as asked by reviewer #1. It might be of interest to add a figure with the proportion of the different haplotypes from the table in fig 6 compared to wt haplotypes over time (for example in a Sankey diagram); overall and by district.

RESPONSE TO SECOND REVIEW COMMENT: *WE THANK THE REVIEWER FOR THIS SUGGESTION. TO BETTER ILLUSTRATE THE TEMPORAL AND GEOGRAPHIC DYNAMICS OF PFDHFR I164L-ASSOCIATED HAPLOTYPES, WE HAVE ADDED A NEW FIGURE 7. PANEL (A) SHOWS CHANGES IN HAPLOTYPE FREQUENCIES OVER TIME USING A SANKEY PLOT, AND PANEL (B) SHOWS THEIR DISTRIBUTION ACROSS SAMPLING SITES USING A HEATMAP. THESE ANALYSES COMPLEMENT THE EXTENDED HAPLOTYPE ANALYSIS IN FIGURE 6 AND HAVE BEEN INCORPORATED INTO THE RESULTS (LINES 229–235, TRACKED VERSION) AND DISCUSSION (LINES 267–270, TRACKED VERSION).*

The methods were described with sufficient clarity.

Several figures in the pdf version for the reviewer were low resolution (Fig 2B, 3, 4,5, 6A).

RESPONSE TO SECOND REVIEW COMMENT: *AS NOTED ABOVE, HIGHER RESOLUTION FIGURES HAVE BEEN PROVIDED.*

Minor comments
line 209: "showed slightly slow EHH decay than wildtype", should be "... slowER ... than .."

RESPONSE TO SECOND REVIEW COMMENT: *THANK YOU. THIS HAS BEEN CORRECTED.*

line 293: sentence starts with process, something seems missing here "This process"?

RESPONSE TO SECOND REVIEW COMMENT: *THANK YOU. THIS HAS BEEN CORRECTED.*
